# Boron Nitride-Doped Polyphenylenic Organogels

**DOI:** 10.1021/acs.chemmater.2c01766

**Published:** 2022-11-18

**Authors:** Jacopo Dosso, Hamid Oubaha, Francesco Fasano, Sorin Melinte, Jean-François Gohy, Colan E. Hughes, Kenneth D. M. Harris, Nicola Demitri, Michela Abrami, Mario Grassi, Davide Bonifazi

**Affiliations:** † School of Chemistry, Cardiff University, Park Place, CF10 3AT Cardiff, U.K.; ‡ Institute of Information and Communication Technologies, Electronics and Applied Mathematics, Université catholique de Louvain, 1348 Louvain-la-Neuve, Belgium; § Institute of Condensed Matter and Nanosciences, Université catholique de Louvain, 1348 Louvain-la-Neuve, Belgium; ∥ Elettra—Sincrotrone Trieste, S.S. 14 Km 163.5 in Area Science Park, 34149 Basovizza—Trieste, Italy; ⊥ Department of Engineering and Architecture, University of Trieste, Via Alfonso, Valerio, 6, I-34127 Trieste, Italy; # Institute of Organic Chemistry, University of Vienna, 1090 Vienna, Austria

## Abstract

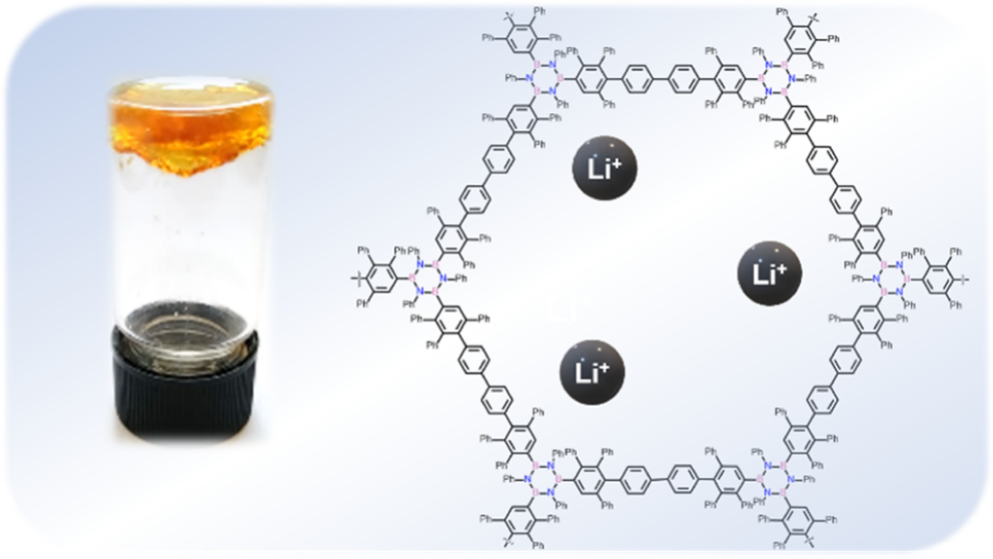

Herein, we describe the synthesis of the first boron nitride-doped polyphenylenic material obtained through a [4 + 2] cycloaddition reaction between a triethynyl borazine unit and a biscyclopentadienone derivative, which undergoes organogel formation in chlorinated solvents (the critical jellification concentration is 4% w/w in CHCl_3_). The polymer has been characterized extensively by Fourier-transform infrared spectroscopy, solid-state ^13^C NMR, solid-state ^11^B NMR, and by comparison with the isolated monomeric unit. Furthermore, the polymer gels formed in chlorinated solvents have been thoroughly characterized and studied, showing rheological properties comparable to those of polyacrylamide gels with a low crosslinker percentage. Given the thermal and chemical stability, the material was studied as a potential support for solid-state electrolytes. showing properties comparable to those of polyethylene glycol-based electrolytes, thus presenting great potential for the application of this new class of material in lithium-ion batteries.

## Introduction

In the last decades, polyphenylene-based materials have been the subject of extensive research efforts.^1,2^ This interest is related to the many different potential applications of these materials, from precursors in graphene synthesis^3,4^ to antennae systems,^5−7^ proton exchange membranes,^8,9^ optoelectronic devices,^10^ and self-assembled systems.^11^ Structural modifications of the polyphenylene-based frameworks by heteroatom-doping^12,13^ or peripheral addition of functional groups^14−16^ are currently the main strategies to enrich the chemical and physical properties of polyphenylenic materials. Polymeric phenylene-based materials are principally prepared by exploiting [4 + 2] cycloaddition reactions with CO extrusion.^17,18^ Particularly, the pioneering work of Müllen and co-workers showed how a large variety of controlled structures, tailored for a given application, can be prepared through an appropriate combination of specifically designed dienes and alkyne precursors.^19^

**Figure 1:**
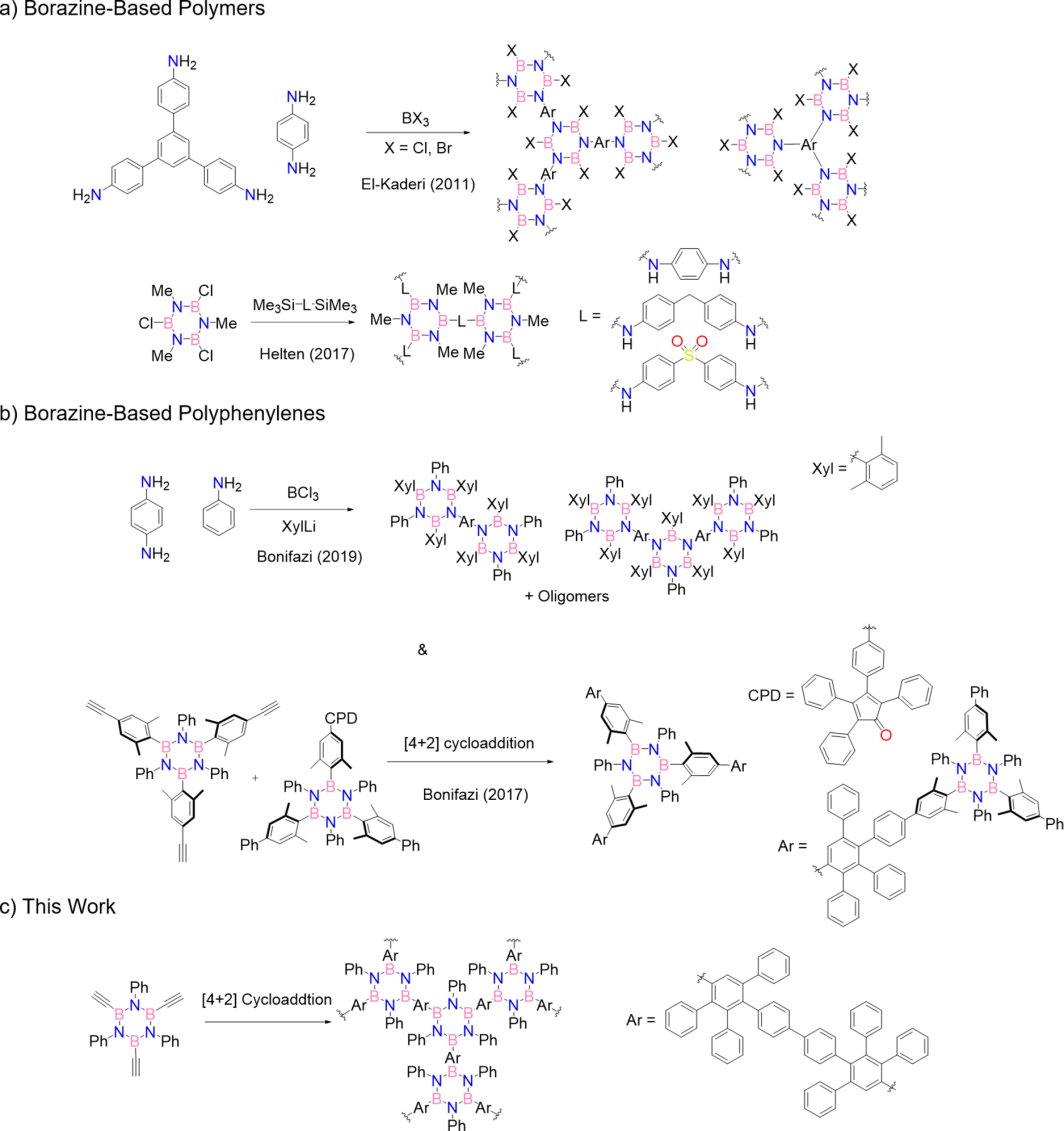
From molecular to macromolecular borazino-phenylenes.

We contributed to the field by preparing discrete borazino-doped oligophenylenes following the [4 + 2] cycloaddition with CO extrusion synthetic route using opportunely functionalized ethynyl- and tetraphenylcyclopentadienone-bearing borazine derivatives (Figure 1b).^20^ The replacement of a phenyl ring with a borazine ring proved to be particularly effective in breaking the π-conjugation, causing a blue shift in the emission envelopes compared to those of the all-carbon congeners.^20,21^ Borazino-doped oligophenylenes can also be prepared by exploiting a condensation reaction approach, either condensing previously formed chloroborazoles^22^ via a silicon/boron exchange (Figure 1a)^23^ or by using a mixture of *para*-phenylendiamine and aniline with BCl_3_, followed by the addition of an aryl lithium derivative (Figure 1b).^21^ When only arylendiamine derivatives are used, boron nitride (BN)-doped polymeric materials can be obtained in which chloro-borazole units are formed (Figure 1a).^24,25^

In this case, the moisture-sensitive nature of the chloro-borazole units limits the manipulation of these materials under normal ambient conditions and consequently limits the versatility of their applications. The presence of BN (or BO) bonds proved to increase the affinity of the materials toward polar or charged species,^24,26^ which is particularly important when engineering materials for gas adsorption/storage applications^24,25^ and solid-state electrolytes (SSEs).^27,28^ Moreover, the insertion of the thermally stable BN bonds has frequently led to the use of BN polymers as pre-ceramic/hybrid materials.^29−31^ With the objective of preparing porous functional borazine-doped materials, this paper tackles the challenge of engineering borazino-bearing polyphenylene-based polymeric structures. When devising the preparation and structure of these materials, we must consider the susceptibility of the BN core toward moisture.^32,33^ Up to now, we have circumvented this problem by preparing borazine precursors bearing 2,6-disubstituted aryl moieties at the B-position, with the 2,6-substituents sitting atop the boron atoms, shielding them from possible hydrolytic reaction (Figure 1b).^32,33^ On the one hand, although this approach has been effective in providing robust borazine linkers for preparing porous metal–organic frameworks,^34^ the preparation of sterically protected borazine precursors is synthetically demanding and could be a limiting factor in expanding the chemical space of these BN precursors. On the other hand, the preparation of borazino-doped polyphenylenic materials by exploiting borazine formation as the polymerization reaction would require the use of strongly reactive BCl_3_ and BBr_3_ boron sources.^24,25^ Moreover, it would be very difficult to functionalize the B-atoms with protecting aryl substituents in a later step due to the generally low solubility of the final materials and the high steric hindrance surrounding the B-atoms.^21^ Based on these considerations, we concluded that a desirable synthetic approach would involve a borazine precursor that, through a high-yielding reaction, could polymerize and simultaneously place sterically shielding moieties atop the boron centers. This line of thought led us to conjecture that the *B*,*B*′,*B*″-triethynyl-*N*,*N*′,*N*″ triphenyl borazine scaffold,^35^ known to be relatively stable under normal ambient conditions,^36^ could serve as a suitable precursor to undergo polymerization through [4 + 2] cycloaddition with CO extrusion when reacted with a suitable biscyclopentadienone (Figure 1c). If a tetraphenylcyclopentadienone scaffold is used, formation of the tetra-substituted B-aryl moiety would place a phenyl ring atop the boron centers, making the borazine ring inert toward hydrolysis reaction. Embracing this synthetic strategy, we prepared a *B*,*B*′,*B*″-triethynyl-*N*,*N*′,*N*″ triphenyl borazine precursor and explored its potential use to prepare porous materials through [4 + 2] cycloaddition with CO extrusion as the polymerization reaction.

## Results and Discussion

### Synthesis and Structural Characterization of the Borazine-Based Monomeric Units and Polymeric Materials

**Scheme 1:**
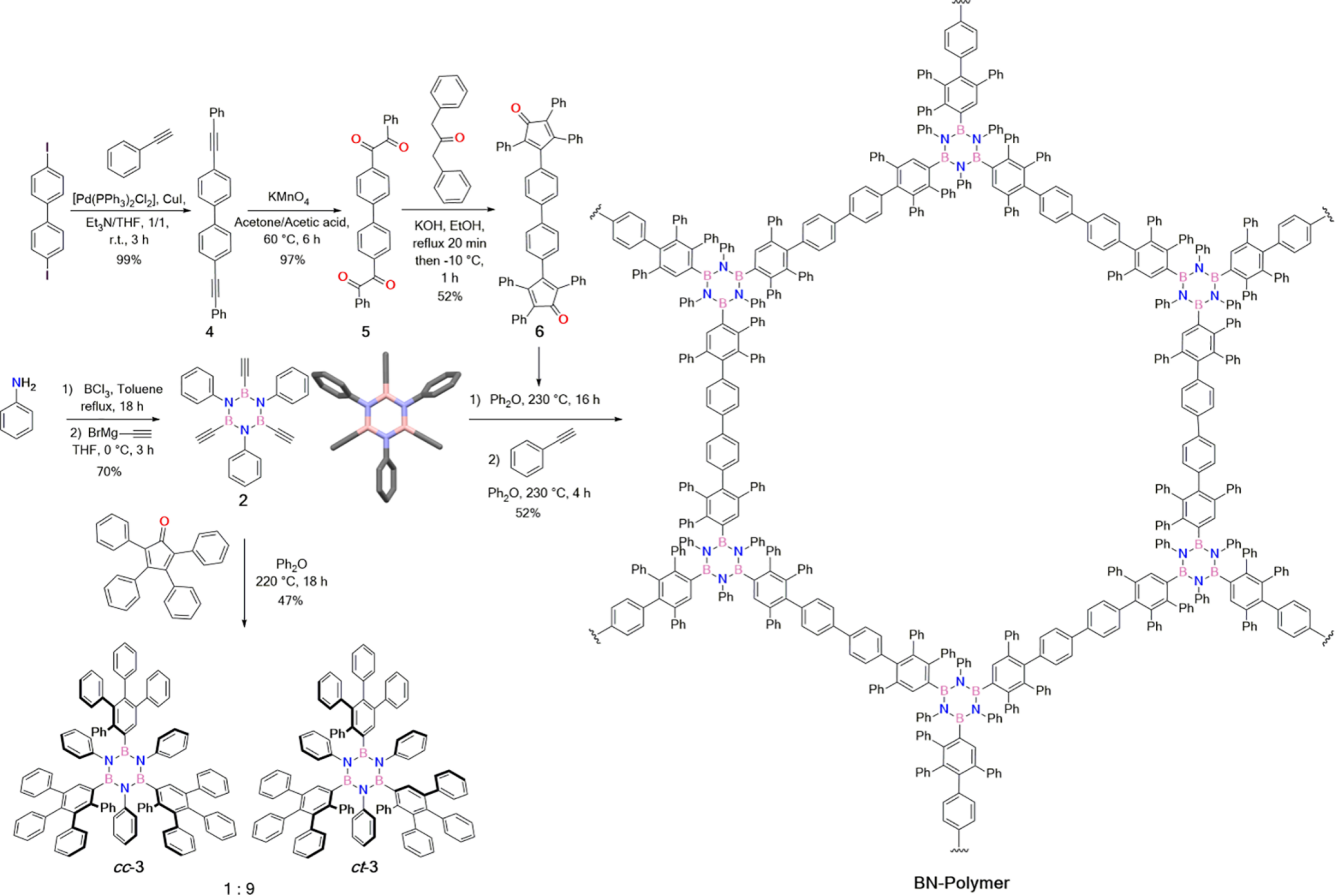
Synthesis of BN-Doped Polymer Starting with Aniline: Monomeric Cycloadduct **3**, Double Dienophile **6**, and **BN-Polymer** Crystal structure of derivative **2** is also shown; space group: *R*3̅.

Our synthetic efforts commenced with the preparation of *B*,*B*′,*B*″-triethynyl-*N*,*N*′,*N*″-triphenyl borazine **2**, which was prepared following a modified procedure^37,38^ from those originally developed by Groszos and Stafiej^39^ and subsequently by Yamaguchi.^40^ Reaction of aniline with BCl_3_ in refluxing toluene gave the corresponding chloro-borazole intermediate which, on reaction with a solution of HC≡CMgBr at 0 °C, gave **2** in 70% yield (Scheme 1). The product proved to be moisture-sensitive but sufficiently stable when stored under anhydrous conditions.^36^ We first studied the [4 + 2] cycloadditions on *B*,*B*′,*B*″-triethynyl borazines using commercially available tetraphenylcyclopentadienone in dry and degassed Ph_2_O at 220 °C (Scheme 1). As expected, product **3** could be obtained in a 47% yield (78% yield for a single reaction) as a mixture of diastereomers (*i.e.*, ***cc*-3** and ***ct*-3** atropoisomers). To estimate the isomeric excess of the cycloaddition reaction, a multidimensional nuclear magnetic resonance (NMR) analysis using ^13^C-DEPTq, ^1^H-^13^C HSQC, and HMBC experiments was carried out in C_6_D_6_ (Figures S17–S20). In the ^1^H NMR spectrum of the atropoisomeric mixture, three singlets were observed at 7.52, 7.45, and 7.32 ppm, integrating as 1, 2, and 0.3 protons, respectively (Figure S20). As the only proton resonances present as singlets are those on the aryl group bonded on the boron atom (H_ct_ and H_cc_), it is reasonable to assume that the proton resonances at 7.52 and 7.45 ppm (in 1:2 ratio) are those of ***ct*-3**, whereas the peak at 7.32 ppm is assigned to ***cc*-3**. From the HSQC experiment, the proton resonances at 7.52 and 7.45 ppm correlate with the ^13^C signals at 136.4 and 135.8 ppm, whereas the signal at 7.32 ppm correlates with the ^13^C peak centered at 135.7 ppm (Figures S19 and S20). As the proton resonances correlate only with quaternary carbons, the correct assignment for the singlet signals was confirmed beyond doubt. In fact, the only protons showing an exclusive correlation with quaternary carbons are those on the B-aryl ring (Figure S20). Integrating the peaks areas for the H_***ct***_ and H_***cc***_ resonances, we estimate that reference cycloadduct **3** was obtained with a 90% ***ct***-atropoisomeric excess.

**Figure 2:**
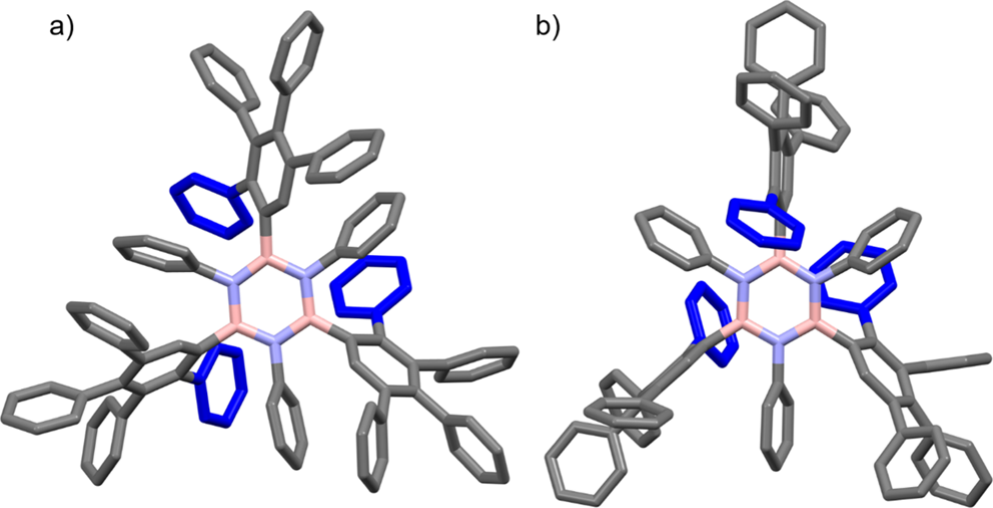
Molecular structures of cycloadduct isomers ***cc*-3** (a) and ***ct*-3** (b) in their crystal structures determined from single-crystal XRD, with space groups *P*6_3_ and *P*1̅, respectively.

Further confirmation of the structures of both isomers was obtained by single-crystal X-ray diffraction (XRD) analysis of crystals grown by slow diffusion of MeOH vapor into a CH_2_Br_2_ solution of a mixture of ***cc*-3** and ***ct*-3** (Figure 2). Two types of crystal were obtained, each containing one isomer (specifically with space group *P*6_3_ for isomer ***cc*-3** and space group *P*1̅ for isomer ***ct*-3**). While ***cc*-3** features all three in-phenyl moieties on the same side of the B_3_N_3_ core, ***ct*-3** has one in-phenyl ring on the opposite side. As discussed in Section S2.1 of the Supporting Information, a powder XRD study of the bulk sample of cycloadduct **3** prepared directly from the chemical reactions shown in Scheme 1, followed by crystallization from CH_2_Br_2_, indicates that the only detectable crystalline phase is the *P*1̅ phase of the CH_2_Br_2_ solvate of **3**, which contains the ***ct*** isomer. We note that other crystalline phases (*e.g.*, containing the ***cc*** isomer) may also be present but are below the detection limit, which is estimated to be several percent given the relatively poor signal-to-noise level of the powder XRD data. This study confirms that the bulk sample of cycloadduct **3** prepared directly from the chemical reactions shown in Scheme 1 contains predominantly the ***ct*** isomer, fully consistent with the evidence from the multidimensional NMR analysis discussed above.

To exploit the cycloaddition reaction to prepare borazine-doped polyphenylenic polymers, we have designed a suitable dimeric diene unit, terminating with two tetraphenylcyclopentadienone moieties (molecule **6**, Scheme 1). The first step of the synthesis involved a double Sonogashira-type cross-coupling reaction between phenylacetylene and 4,4′-diiodobiphenyl to yield bis-alkyne derivative **4** in quantitative yield. Oxidation of molecule **4** in the presence of KMnO_4_ gave tetracarbonyl derivative **5** in 97% yield, which was in turn reacted with 1,3-diphenylacetone to produce the desired dimeric cyclopentadienone **6** in good yield (52%). The structure of biscyclopentadienone **6** was confirmed via NMR spectroscopy and high resolution mass spectroscopy (HRMS) (Figures S27–S29).

**Figure 3:**
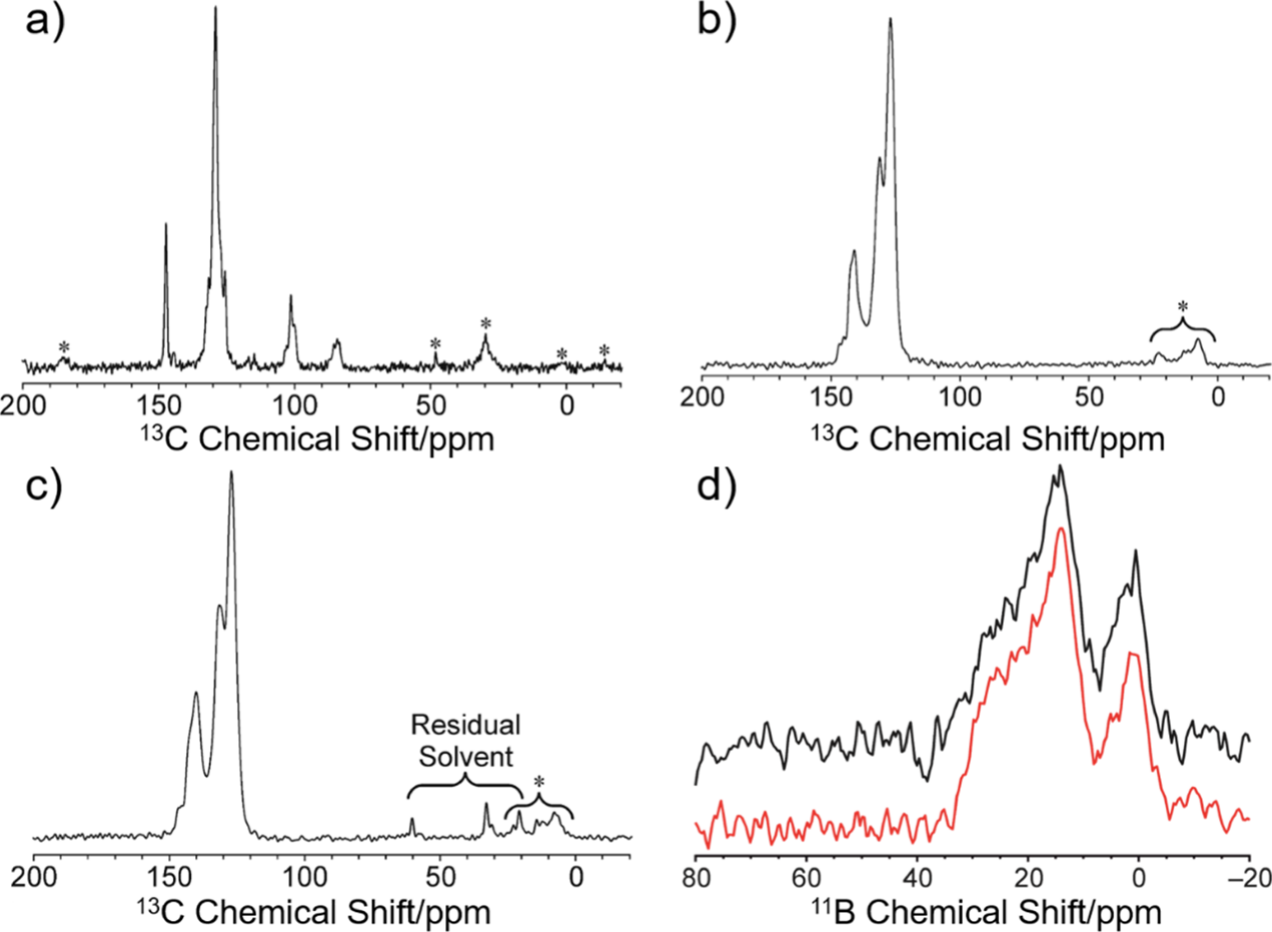
Solid-state ^1^H → ^13^C CPMAS NMR spectra recorded for (a) borazine precursor **2**, (b) reference cycloadduct **3**, and (c) **BN-polymer**. Peaks marked with asterisks are spinning sidebands. (d) One-dimensional projections of the solid-state ^11^B MQMAS NMR spectra onto the direct dimension for cycloadduct **3** (red) and **BN-polymer** (black).

The cycloaddition reaction between borazine **2** and biscyclopentadienone **6** in dry and degassed Ph_2_O at 220 °C yielded a red gel. After precipitation and purification of the solid, the resulting reddish material was reacted with phenylacetylene in Ph_2_O at 230 °C to end-cap the unreacted cyclopentadienone units. Purification of the resulting pale-yellow solid with various sonication–centrifugation cycles using different solvents (petroleum ether/CH_2_Cl_2_, acetone, MeOH, and Et_2_O) gave the desired **BN-polymer** in 52% yield. Given the insolubility of the product in common organic solvents, characterization was carried out by thermogravimetric analysis (TGA), attenuated total reflectance infrared spectroscopy (ATR−IR), and solid-state NMR techniques. TGA measurements showed that the solid is thermally stable up to 400 °C (Figure S30), trapping ca. 10–20% of the solvent (see mass loss at around 100 °C). ATR–IR analysis of both cycloadduct **3** and **BN-polymer** (Figure S31) showed strong signals at ca. 1355 and 1321 cm^–1^ assigned to BN bond stretching. Notably, no signals related to C=O bonds were detected, suggesting the full conversion of the end-capping reaction. High-resolution solid-state ^13^C NMR spectra recorded using the ^1^H → ^13^C CPMAS technique for precursor **2**, reference cycloadduct **3**, and the **BN-polymer** material are shown in Figure 3. The solid-state ^13^C NMR spectrum of precursor **2** has isotropic peaks at 84 and 101 ppm due to the ethynyl moieties and isotropic peaks in the range 125–150 ppm due to aromatic environments. The fact that the ethynyl peaks are absent from the solid-state ^13^C NMR spectra of the cycloadduct **3** and **BN-polymer** samples is consistent with our hypothesis of complete conversion of the cycloaddition reaction. The solid-state ^13^C NMR spectra of the cycloadduct **3** and **BN-polymer** materials contain multiple overlapped peaks between 120 and 150 ppm, corresponding to the aromatic ^13^C environments in these materials. The fact that the solid-state ^13^C NMR spectra of cycloadduct **3** and **BN-polymer** are virtually identical in this region (see the overlay of the spectra in Figure S36) suggests that the local structure around the central aromatic ring (including the conformations of the substituents) is very similar in these materials. Low-intensity peaks between ca. −15 and 60 ppm are assigned to spinning sidebands and, in the case of **BN-polymer**, also to the residual solvent (assigned as Et_2_O and MeOH), as annotated on the spectra in Figure 3b,c.

Solid-state ^11^B MQMAS NMR spectra^41^ recorded for samples of cycloadduct **3** and the **BN-polymer** are shown in Figure S37, and one-dimensional projections of these spectra onto the direct dimension are shown in Figure 3d. The one-dimensional projections in Figure 3d were obtained from the ^11^B MQMAS NMR spectra by summation of all data between 39 and 49 ppm along the indirect dimension (vertical in Figure S37; see details in the Supporting Information). In the one-dimensional projections in Figure 3d, the shapes of the peaks are very similar for cycloadduct **3** and the **BN-polymer**, strongly suggesting that cycloadduct **3** and the **BN-polymer** have similar values of the quadrupolar parameters and isotropic chemical shifts for the ^11^B environments. Again, we may infer from this observation that the local structures in the vicinity of the central B_3_N_3_ rings (including the conformations of the substituents bonded to the B atoms) are very similar in cycloadduct **3** and the **BN-polymer**.

### Gel Formation and Rheological Studies

As observed during the preparation of the polymer in Ph_2_O, **BN-polymer** shows a tendency to form gels. By suspending **BN-polymer** in different solvents, it was observed that chlorinated solvents (such as CHCl_3_ and CH_2_Cl_2_) give gels upon sonication with a critical jellification concentration of 4 wt % for CHCl_3_. Low-field ^1^H NMR (LF-NMR) studies (Table 1) were then carried out to study the jellification behavior at three different concentrations to obtain data within the sub-gelated (2%) to super-gelated (8%) systems. In this analysis, the relaxation time *T*_2_ was considered since it depends not only on the magnetic field strength (*B*_0_) and temperature but also on the presence of solid surfaces such as those typical of a polymeric gel. The relaxation process is faster with increasing concentration of the “solid” component, which is reflected in a smaller *T*_2_. In the case of non-homogeneous systems, where meshes of different sizes can exist, the average *T*_2_ depends on the relaxation time arising from each different mesh size present in the gel. In general, the ^1^H relaxation is faster with decreasing mesh size.

**Table 1:** Values of *T*_2_ from LF-NMR for Gels Formed with Various Concentrations of **BN-Polymer** in CHCl_3_

**BN-polymer** wt %	*T*_21_ (ms)	*A*_1_ (%)	*T*_22_ (ms)	*A*_2_ (%)	*T*_23_ (ms)	*A*_3_ (%)	*T*_24_ (ms)	*A*_4_ (%)
0%	2531	100						
2%	2373	81	1350	19				
4%	1927	73	981	18	108	9		
8%	1537	51	635	20	133	11	23	17

As expected, *T*_2_ is observed to decrease as the polymer concentration increases (Table 1). When moving from 2 to 8% concentration, an increasing number of relaxation times (*T*_2i_ values) are detected, reflecting a higher heterogeneity of the jellifying network and suggesting the presence of meshes of different sizes (with small *T*_2i_ times corresponding to small cavity size). From the rheological point of view, we analyzed the 4 wt % gel by short stress sweep (SSS) and frequency sweep (FS) measurements. From SSS measurements (Figure S39), the presence of an elastic modulus *G*′ much greater than the viscous modulus *G*″ further confirms the formation of a jellified material.

**Figure 4:**
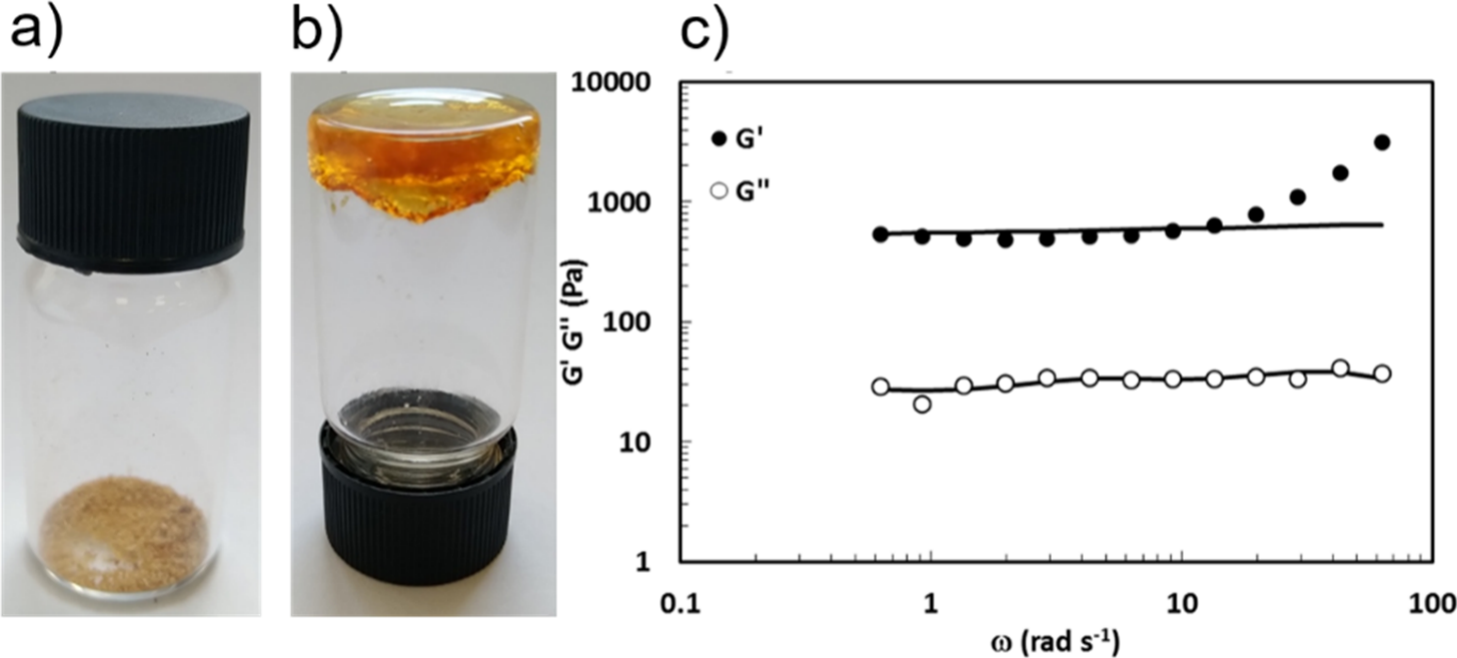
(a) Xerogel and (b) organogel of **BN-polymer**; (c) FS plot (circles) and Maxwell fitting (solid lines) for the 4 wt % gel in CHCl_3_.

FS measurements (Figure 4) along with Maxwell best fitting (solid lines) allowed an estimation of the shear modulus (*G*), the cross linking (ρ_*x*_), and the mesh size (ξ_RHEO_) of the gel, giving *G* = 667 ± 54 Pa, ρ_*x*_ = 2.7 × 10^–7^ ± 2.2 × 10^–8^, and ξ_RHEO_ = 23.0 ± 0.6 nm. The value of *G* is comparable to that of a polyacrylamide gel with a low crosslinking percentage,^42^ which is in line with the low cross-linking value obtained from the measurement. The mesh size is consistent with a mesoporous material; however, the isotherm of N_2_ adsorption at 77 K, measured on the xerogel, indicates a macroporous/non-porous material (Figure S45) with a calculated apparent Brunauer–Emmett–Teller surface area of 62 m^2^/g, which suggests that the porous structure collapses upon drying.

### **BN-Polymer** as Support in SSEs

**Figure 5:**
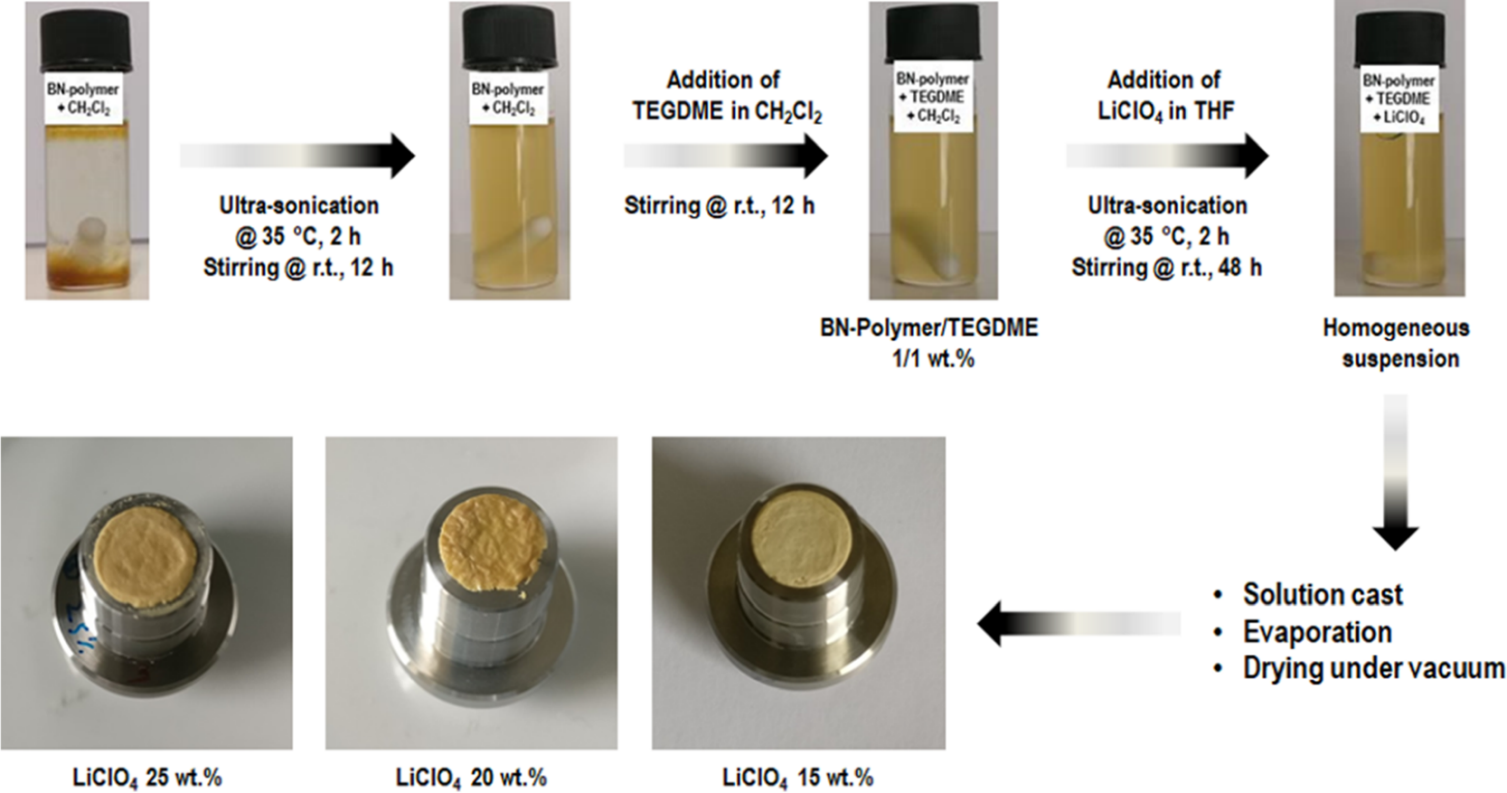
Process to prepare the **BN-polymer**/TEGDME/LiClO_4_-based SSEs.

The ability to form gels, the thermal stability, and the presence of polar BN bonds (see, *e.g.*, the case for boroxines^27,28^) make this material a good candidate as the support component in Li^+^-containing SSEs. Thus, **BN-polymer**/TEGDME (tetraethylene glycol dimethyl ether)/LiClO_4_-based SSEs were prepared following the process shown in Figure 5 and tested (the technical details are reported in the Supporting Information). **BN-polymer** was first suspended in CH_2_Cl_2_ (2 mL) under ultra-sonication at 35 °C for 2 h followed by vigorous stirring at r.t. for 12 h. Then, the TEGDME solution in CH_2_Cl_2_ (0.5 mL) was added and the obtained mixtures stirred at r.t. for 12 h. Finally, the LiClO_4_ solutions at different concentrations (15, 20, and 25 wt %) in THF (0.5 mL) were added to the **BN-polymer**/TEGDME suspensions and ultra-sonicated at 35 °C for 2 h followed by vigorous stirring at r.t. for 48 h so that homogeneous suspensions were obtained. The SSEs were deposited by solution cast on stainless steel disks adapted for Swagelok cells and dried at r.t. for 18 h followed by drying under vacuum at 70 °C for 2 h.

**Figure 6:**
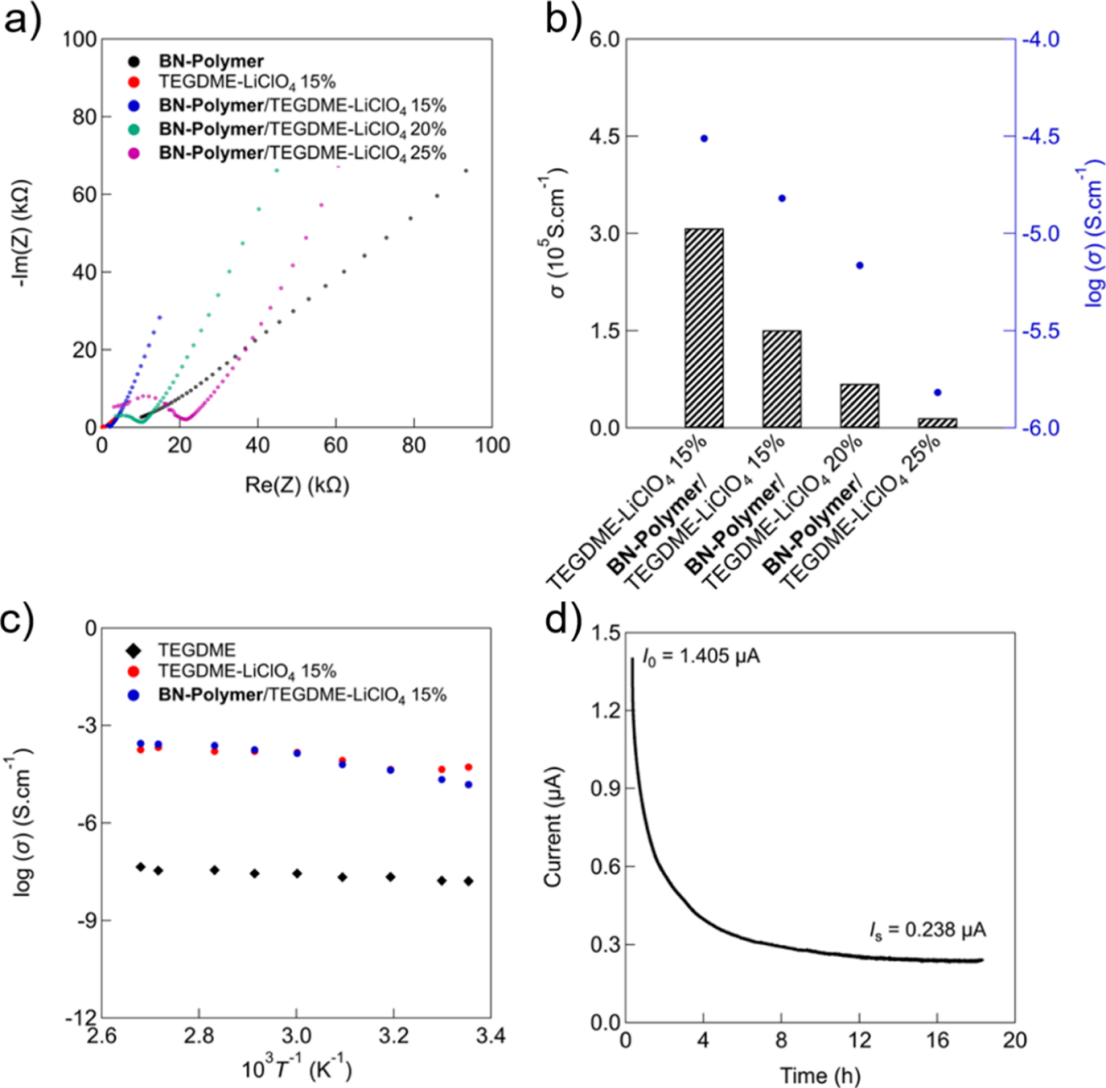
(a) Nyquist plots of pure **BN-polymer**, TEGDME/15 wt % LiClO_4_, and **BN-polymer**/TEGDME/*x* LiClO_4_ (*x* = 15, 20, or 25 wt % of LiClO_4_) at r.t. (b) Ionic conductivity at r.t. of TEGDME/15 wt % LiClO_4_ reference and **BN-polymer**-based SSEs as a function of LiClO_4_ content. (c) Arrhenius plots of ionic conductivity vs reciprocal absolute temperature for TEGDME, liquid TEGDME/15 wt % LiClO_4_, and **BN-polymer**/TEGDME/15 wt % LiClO_4_ electrolytes. (d) Current vs time profile of a symmetrical Li/**BN-polymer**/TEGDME/15 wt % LiClO_4_/Li cell configuration after applying a DC voltage of 10 mV on the cell for determining the Li^+^ transfer number.

The ion transport behavior of the SSEs prepared in this way was investigated by electrochemical impedance spectroscopy at r.t., using TEGDME/15 wt % LiClO_4_ liquid electrolyte as the reference (Figures 6a, S44 and Table 2). Solid electrolytes containing **BN-polymer** showed a decrease in ionic conductivity with increasing salt content, with the highest ionic conductivity of about 1.51 × 10^–5^ S cm^–1^ for the sample containing 15 wt % of LiClO_4_ (Figure 6b and Table 2). The bulk electrolyte resistance value (*R*_b_) increases with increasing amount of LiClO_4_ incorporated into the **BN-polymer**/TEGDME matrix due to a decrease in the available coordination sites of TEGDME by ion pairing, which is consistent with the ether crown ion chelation mechanism.^43−45^ This could be due to the distance between dissociated ions becoming too small and thus enabling the recombination into neutral ion pairs. This in turn leads to the formation of large aggregates of ions that reduce the segmental motion of ethylene oxide sites in the network, thus hampering the polymer motion (which is essential for fast ion conduction).^46−51^

**Table 2:** Characteristic Properties of the Prepared SSEs at r.t. as a Function of Lithium Salt Content; *t*: Thickness, *A*: Surface Area of the Specimen, *R*_b_: Bulk Electrolyte Resistance, and σ: Ionic Conductivity

	wt %						
sample	**BN**-**polymer**	TEGDME	LiClO_4_	*t* (cm)	*A* (cm^2^)	*R*_b_ (Ω)	σ (S cm^–1^)
0	100			0.018	1.33		
1		85	15	0.02159	1.33	526	3.08 × 10^–5^
2	42.5	42.5	15	0.02451	0.79	2060	1.51 × 10^–5^
3	40	40	20	0.03	0.79	5580	6.80 × 10^–6^
4	37.5	37.5	25	0.036	0.79	20586	1.50 × 10^–6^

The estimated values of *E*_a_ are listed in Table 3.^52^ The lower the value of activation energy in the system, the higher the ionic conductivity at r.t. and vice versa.^53^ At low temperatures, the **BN-polymer**-based electrolyte exhibits a lower ionic conductivity than that of the liquid TEGDME/15 % wt. LiClO_4_ system. However, it presents a similar trend compared to the liquid TEGDME/15 % wt. LiClO_4_ system at high temperatures. The lower activation energy for ion migration at high temperatures is probably due to the amorphous nature of the polymer, which promotes more free volume in the polymer electrolyte system upon increasing temperature.^54,55^ Furthermore, the wettability of **BN-polymer** pores by TEGDME increases at high temperatures, leading to a softer network, and TEGDME chains act as a transport medium.^56^ In addition, the interaction of lithium salts with TEGDME and the presence of BN polar bonds help to weaken the ionic interaction in the salt and thus improve the charge carriers mobility.^52,57^ Since conductivity is influenced by temperature, σ was studied as a function of *T* for pure TEGDME, liquid TEGDME/15 wt % LiClO_4_, and **BN-polymer**/TEGDME/15 wt % LiClO_4_ (Figure 6c). The ionic conductivity of the **BN-polymer**/TEGDME/15 wt % LiClO_4_ electrolyte increases up to 2.7 × 10^–4^ S cm^–1^ at 373 K. Figure 6c shows a linear variation in plots of log (σ) versus 10^3^/*T*, suggesting an Arrhenius-type thermally activated process:^58^ σ = σ_0_ exp(−*E*_a_/*kT*) where σ is the conductivity, *E*_a_ is the activation energy associated with conduction, *k* is the Boltzmann constant, *T* is the temperature, and σ_0_ is a preexponential term.

**Table 3:** Room Temperature (25 °C) and 100 °C Values of Ionic Conductivity as Well as Activation Energy Values for the TEGDME, Liquid TEGDME/15 wt % LiClO_4_, **BN-Polymer**/TEGDME/15 wt % LiClO_4_ Electrolytes

sample	σ (S cm^–1^) 25 °C	σ (S cm^–1^) 100 °C	*E*_a_ (eV) 25–100 °C
pure TEGDME	1.62 × 10^–8^	4.43 × 10^–8^	0.12
liquid TEGDME/15 wt % LiClO_4_	3.08 × 10^–5^	1.18 × 10^–4^	0.20
**BN-polymer**/TEGDME/15 wt % LiClO_4_	1.51 × 10^–5^	2.75 × 10^–4^	0.38

To investigate how the morphological changes of the **BN-polymer**/TEGDME/LiClO_4_-based electrolytes could affect the performance, the films were subjected to scanning electron microscopy (SEM) analysis (Figure S41). Pure **BN-polymer** (see Figure S41a) has a “sponge-like” structure containing cavities with an average diameter in the micrometer range (1–20 μm), whereas no pores are visible in the **BN-polymer**/TEGDME/LiClO_4_ composite (Figure S41b–d). For the 15 wt % LiClO_4_ sample, the higher conductivity could be attributed to the relatively homogenous structure, implying a good dispersion of all components in the electrolyte film (Figure S41b). However, the 20 wt % LiClO_4_ sample presents spherical aggregates with an average diameter in the micrometer range, which could be ascribed to the lithium salt not completely dissolved in the polymer matrix (Figure S41c). Increasing the amount of the Li^+^ salt to 25 wt % leads to a further decrease in the conductivity of the SSE. This could be attributed to the bigger aggregates formed in the polymer matrix leading to a microstructure that is interspersed with big pores (Figure S41d).

The thermal stability of the **BN-polymer**/TEGDME/LiClO_4_ SSEs was studied by TGA (measured under a N_2_ atmosphere at a heating rate of 10 °C min^–1^ from r.t. to 1000 °C, Figure S42a) and differential scanning calorimetry (DSC), (measured under a N_2_ flow at a ramp rate of 10 °C min^–1^, Figure S42b) on both the single components and the films. As expected, **BN-polymer**/TEGDME/LiClO_4_ samples display the same thermal behavior as that of the single components. A gradual weight loss of about 5% is observed during the initial heating up to 100 °C, which can be attributed to the loss of moisture present in the electrolytes. The first significant weight loss (about 45–55%) is due to the evaporation or degradation of TEGDME at 150 °C, followed by a gradual loss of about 5% between 300 and 450 °C due to LiClO_4_. The complete decomposition of the sample takes place between 500 and 600 °C with a weight loss of about 75%, in line with the TGA data on the pristine **BN-polymer** (Figure S30). The DSC results (Figure S42b) for **BN-polymer**/TEGDME/LiClO_4_ in the range 30–250 °C show no endothermic peak associated with melting, which is a further demonstration of the amorphous nature of the polymer and the polymer–salt systems. The absence of a temperature of glass transition suggests the high rigidity of **BN-polymer**, possibly related to the blocking of segmental motion, which would be consistent with the low conductivity. The electrochemical stability of the highest conducting SSE system was also studied with linear sweep voltammetry (LSV) and cyclic voltammetry (CV) using a stainless steel working electrode and Li metal as the reference and counter electrodes. The SSE system proved to be electrochemically stable between 1.84 and 3.70 V versus Li/Li^+^ with no decomposition of any of the components in this potential region (Figure S43a). The CV responses for seven cycles (Figure S43b) present redox peaks around 1.55 and at 3.86 V corresponding to anodic oxidation and cathodic reduction, respectively. While no changes in the redox peak voltages are observed during the cycles, the overlapping of the sweeps indicates that the charge-transfer reaction at the interface between the solid electrolyte and Li metal is reversible. From the CV study, the range of electrochemical stability goes from 2.00 to 3.60 V, which is in very good accordance with LSV results (Figure S43a). Finally, the Li-ion transference number (*t*_Li^+^_) was evaluated following the potentiostatic polarization method. A symmetric Li/**BN-polymer**/TEGDME/15 wt % LiClO_4_/Li cell configuration was used in this study, and a DC polarization with 10 mV potential was applied until the current reached a steady state (Figure 6d). The initial total current decreases with time from 1.405 μA and reaches a steady state value of 0.238 μA after 18 h. The interfacial bulk resistance increases from 2260 to 21,713 Ω through polarization. Thus, the **BN-polymer**/TEGDME/15 wt % LiClO_4_ electrolyte presents a transference number of about 0.2, which is in the range of those observed for polyethylene glycol (PEG)-based electrolytes.^56^

## Conclusions

In conclusion, we have reported on a unique BN-doped gel material obtained through a polymerization reaction exploiting the [4 + 2] cycloaddition with CO extrusion between B-ethynyl-substituted borazine and a suitable biscyclopentadienone derivative. Considering that only a few examples of [4 + 2] cycloadditions are reported on alkynes directly bonded to boron atoms,^59−61^ to the best of our knowledge, this is the first example of a Diels Alder cycloaddition for a B-alkynyl functionalized borazine. Solid-state ^13^C NMR, solid-state ^11^B NMR, and Fourier-transform infrared (FT-IR) spectroscopic characterization were used to confirm the formation of a BN-doped polymeric material, which was characterized by comparison with the monomeric unit. The new BN-doped polyphenylenic material efficiently forms organogels in chlorinated solvents, producing the only example to date of a borazine-doped gel. Rheological investigations confirmed the formation of the gel and allowed us to estimate the shear modulus, the mesh size, and the crosslinking density in the gel. Since the organogel material has high thermal and chemical stability, it was integrated as a support component in a SSE for lithium-ion batteries. In particular, the SSE with **BN-polymer**/TEGDME/15 wt % LiClO_4_ composition exhibits an Arrhenius behavior and a r.t. ionic conductivity of 1.51 × 10^–5^ S cm^–1^ with a Li^+^ transference number of about 0.2, which is comparable to that of PEG-based electrolytes. Furthermore, the electrical conductivity studies suggest an enhanced performance of the 15 wt % SSE at higher temperatures. This result represents a stepping stone toward future potential applications of BNC materials in the field of lithium-ion batteries.

## Experimental Section

### Instrumentation

Thin-layer chromatography was conducted on pre-coated aluminum sheets with 0.20 mm Merk Millipore silica gel 60 with fluorescent indicator F254. Column chromatography was carried out using Merck Gerduran silica gel 60 (particle size 40–63 μm). Melting points were measured using a Gallenkamp apparatus in open capillary tubes and have not been corrected. NMR spectra were recorded with a Bruker Fourier 300 MHz spectrometer equipped with a dual (^13^C, ^1^H) probe, a Bruker AVANCE III HD 400 MHz NMR spectrometer equipped with a Broadband multinuclear (BBFO) SmartProbe, or a Bruker AVANCE III HD 500 MHz spectrometer equipped with Broadband multinuclear (BBO) Prodigy CryoProbe. ^1^H spectra were obtained at 500, 400, or 300 MHz, ^13^C spectra were obtained at 75, 100, or 126 MHz, and ^11^B were obtained at 128 or 224 MHz; all spectra were obtained at r.t. if not otherwise stated. Chemical shifts were reported in ppm according to tetramethylsilane using the solvent residual signal as an internal reference (CDCl_3_: δ_H_ = 7.26 ppm, δ_C_ = 77.16 ppm; CD_2_Cl_2_: δ_H_ = 5.32, δ_C_ = 54.00, C_6_D_6_ δ_H_ = 7.16 ppm, δ_C_ = 128.6). Coupling constants (*J*) were given in hertz. Resonance multiplicity was described as s (singlet), d (doublet), t (triplet), dd (doublet of doublets), dt (doublet of triplets), td (triplet of doublets), q (quartet), m (multiplet), and bs (broad signal). Carbon spectra were acquired with a complete decoupling for the proton. LF NMR was performed at 20 °C with a Bruker Minispec mq 20 (0.47 T, Germany). The determination of the average water protons transverse (spin–spin) relaxation time (*T*_2m_) was performed according to the CPMG sequence (Carr–Purcell–Meiboom–Gill) {90°[−τ – 180° – τ(echo)]_*n*_ – *T*_R_} with a 8.36 μs wide 90° pulse, τ = 250 μs, and *T*_R_ (sequences repetition rate) equal to 5 s. Solid-state NMR ^1^H → ^13^C CPMAS NMR spectra were recorded with a Bruker Avance III HD spectrometer at 9.4 T [Larmor frequencies: 400.2 MHz (^1^H), 100.6 MHz (^13^C)] using ramped CP for the borazine precursor **2**, the reference **3**, and the **BN-polymer** material, with magic angle spinning frequencies of 10, 12, and 12 kHz, respectively. ^11^B MQMAS NMR spectra were recorded with a Bruker Avance III HD spectrometer at 9.4 T [Larmor frequency: 128.4 MHz (^11^B)] using a four-pulse split-*t*_1_ sequence with the indirect dimension scaled to have the same contribution from the isotropic shift as the direct dimension (please refer to the Supporting Information for more details). Powder XRD data for the crystallized sample of 3 (prepared by diffusion of MeOH into a solution of 3 in CH2Br2) were recorded at 21 °C on a Bruker D8 diffractometer (Ge-monochromated CuKα1 radiation; transmission mode; Våntec detector covering 3° in 2θ; 2θ range, 4° to 50°; step size, 0.016°; data collection time, 119.5 hr). IR spectra were recorded with a Shimadzu IR Affinity 1S FTIR spectrometer in ATR mode with a diamond mono-crystal. Mass spectrometry: (i) high-resolution ESI mass spectra were obtained with a Waters LCT HR TOF mass spectrometer in the positive or negative ion mode. (ii) High-resolution MALDI mass spectra were obtained with a Bruker Autoflex speed MALDI-TOF instrument; the sample was prepared with a 1:1 ratio of sample to the matrix DCTB (15 mg/mL) in CH_2_Cl_2_; all these analyses were carried out at Cardiff University. Rheological measurements were performed using a stress-controlled rotational rheometer (Haake Mars Rheometer, 379–0200 Thermo Electron GmbH, Karlsruhe, Germany) equipped with parallel plate geometry (PP35, ϕ = 35 mm, serrated surfaces to avoid slippage at the wall). The measuring device was kept at 10 °C inside a glass bell at saturated conditions to avoid evaporation effects. *SSEs*. TGA was performed using a TGA/SDTA-851 instrument from METTLER TOLEDO at a heating rate of 10 °C min^–1^ from room temperature to 1000 °C under N_2_ flow. DSC was performed with a METTLER TOLEDO DSC-1 STAR^e^ system instrument under a N_2_ flow at a ramp rate of 10 °C min^–1^ in the temperature range of 30–250 °C. Powder XRD was performed with a STOE StadiP X-ray diffractometer with Cu Kα_1_ radiation (λ = 1.5418 Å) from 2θ = 1.5 up to 60° with a 0.02° increment using an operation voltage and current of 40 kV and 40 mA, respectively. SEM images were obtained with a FEI XL30 FEG equipped with a Raith laser interferometer-controlled stage operated at 10 kV and the Elphy Plus software. Electrochemical measurements of the optimized films of **BN-polymer**/TEGDME/LiClO_4_-based SSEs were studied using a Biologic Science Instruments VMP-300 potentiostat analyzer.

### Materials and Methods

#### Synthesis

Chemicals were purchased from Sigma-Aldrich, Acros Organics, TCI, Apollo Scientific, Alfa Aesar, and Fluorochem and were used as received. Solvents were purchased from Fisher Scientific, while deuterated solvents were from Eurisotop and Sigma-Aldrich. THF and toluene were dried on a Braun MB SPS-800 solvent purification system and further dried over activated 4 Å molecular sieves. Diphenyl ether (Ph_2_O) was dried over 4 Å molecular sieves. Aniline was distilled from CaH_2_ under reduced pressure and stored away from light in a N_2_ atmosphere. Aniline was left on CaH_2_ overnight prior to distillation. Low-temperature baths were prepared using different solvent mixtures depending on the desired temperature: −84 °C with ethyl acetate/liq. N_2_, −10 °C with ice/NaCl, and 0 °C with ice/H_2_O. Anhydrous conditions were achieved by drying Schlenk tubes or two-neck flasks by flaming with a heat gun under vacuum and purging with N_2_. The inert atmosphere was maintained using N_2_-filled balloons equipped with a syringe and needle that was used to penetrate the silicon stoppers used to close the flask’s necks. Additions of liquid reagents were performed using plastic syringes. Degassing of solutions was performed using the freeze–pump–thaw procedure: solutions were frozen using liquid N_2_ and kept under vacuum for 10 min before thawing. Alternatively, degassing was performed by bubbling N_2_ in the reaction solution under sonication for at least 10 min. Molecular sieves (4 Å) were activated by heating at 165 °C under vacuum overnight and by further heating with heat gun under vacuum immediately before use. All reactions were performed in dry conditions and under inert atmosphere unless otherwise stated. All procedures for the synthesis are reported in the Supporting Information. *SSE*. Lithium perchlorate LiClO_4_ (battery grade) was obtained from Acros and used without further purification. TEGDME from Sigma-Aldrich was dried under vacuum at 70 °C for 48 h before use. CH_2_Cl_2_ and THF (spectroscopy grade) were purchased from VWR and were used as received.

## Supporting Information Available

Details of experimental methodologies and NMR, crystallographic, electrochemical, and rheological characterization of compounds (PDF)
Crystal structure of 2 (CIF)
Crystal structure of ***ct*-3** (CIF)
Crystal structure of ***cc*-3** (CIF)
Crystal structure of **7** (CIF)

## Supplementary Material

cm2c01766_si_001.pdf

cm2c01766_si_002.cif

cm2c01766_si_003.cif

cm2c01766_si_004.cif

cm2c01766_si_005.cif
