# Supplementary material for: Boron Nitride-Doped Polyphenylenic Organogels
Source: Chem Mater. 2022 Nov 18;34(23):10670–80. doi: 10.1021/acs.chemmater.2c01766 (PMC9753561; doi:10.1021/acs.chemmater.2c01766)
Supplement: Supplementary file 1 — cm2c01766_si_001.pdf [file cm2c01766_si_001.pdf]

Supporting Information

Boron-nitride-doped polyphenylenic organogels

Jacopo Dosso,[a,b] Hamid Oubaha,[c] Francesco Fasano,[a] Sorin Melinte,[c] Jean-François Gohy,[d] Colan E. Hughes,[a] Kenneth D. M. Harris,[a] Nicola Demitri,[e] Michela Abrami,[f] Mario Grassi[f], and Davide Bonifazi*[a,g]

Table of contents

1 General Remarks 3

1.1 Instrumentation 3

1.2 Materials and methods 4

2 Single-crystal X-ray Diffraction measurements 4

2.1 Powder XRD Results on the Crystallized 3 Sample 7

3 Synthetic procedures and spectral data 13

Synthesis of *B,B',B"*-Triethynyl-*N,N',N"*-tri(phenyl)-borazine (2) 13

Synthesis of *B,B',B"*-Tri(3',4'-diphenyl-1,1':2',1''-terphenyl)-*N,N',N"*-tri(phenyl)-borazine (3, *cc* and *ct* isomers). 14

Synthesis of 4,4'-Bis(phenylethynyl)-1,1'-biphenyl (4) 15

Synthesis of 2,2'-([1,1'-Biphenyl]-4,4'-diyl)bis(1-phenylethane-1,2-dione) (5) 15

Synthesis of 4,4'-([1,1'-Biphenyl]-4,4'-diyl)bis(2,3,5-triphenylcyclopenta-2,4-dien-1-one) (6) 16

Synthesis of BN-Polymer* 16

Synthesis of *B,B',B''*-tripropynyl-*N,N',N''*-triphenyl-borazine (7) 17

4 NMR-HRMS spectroscopic characterization (^{1}H, ^{13}C, ^{11}B, HRMS) 18

4.1 Characterization of *B,B',B"*-Triethynyl-*N,N',N"*-tri(phenyl)-borazine (2) 18

4.2 Characterization of *B,B',B"*-Tri(3',4'-diphenyl-1,1':2',1''-terphenyl)-*N,N',N"*-tri(phenyl)-borazine (3, *cc* and *ct* isomers). 20

4.3 Characterization of 1,4-Bis(phenylethynyl)benzene (4) 25

4.4 Characterization of 2,2'-(1,4-Phenylene)bis(1-phenylethane-1,2-dione) (5) 27

4.5 Characterization of 4,4'-([1,1'-Biphenyl]-4,4'-diyl)bis(2,3,5-triphenylcyclopenta-2,4-dien-1-one) (6) 29

4.6 Characterization of BN-Polymer 30

4.7 Characterization of *B,B',B''*-tripropynyl-*N,N',N''*-triphenyl-borazine (7) 31

5 Characterization of BN-Polymer 34

5.1 Solid-State $^{1}H \rightarrow ^{13}C$ CPMAS NMR and Solid-State ^{11}B-MQMAS NMR 34

5.2 BN gel studies 35

6 Solid-state electrolyte characterization 39

6.1 Characterization 39

6.2 XRD analysis and SEM analysis 41

6.3 Thermal analysis 42

6.4 Electrochemical study 43
7 Brunauer-Emmett-Teller measurements on BN-Polymer 46
8 References 46

1 General Remarks

1.1 Instrumentation

Thin layer chromatography (TLC) was conducted on pre-coated aluminum sheets with 0.20 mm *Merk Millipore* Silica gel 60 with fluorescent indicator F254. ***Column chromatography*** was carried out using *Merck Gerduran* silica gel 60 (particle size 40-63 μm). ***Melting points*** (mp) were measured on a *Gallenkamp* apparatus in open capillary tubes and have not been corrected. ***Nuclear magnetic resonance:*** (NMR) spectra were recorded on a Bruker Fourier 300 MHz spectrometer equipped with a dual (^{13}C, ^{1}H) probe, a Bruker AVANCE III HD 400MHz NMR spectrometer equipped with a Broadband multinuclear (BBFO) SmartProbe™ or a Bruker AVANCE III HD 500MHz Spectrometer equipped with Broadband multinuclear (BBO) Prodigy CryoProbe. ^{1}H spectra were obtained at 500, 400 or 300 MHz, ^{13}C spectra were obtained at 75, 100 or 125 MHz NMR, and ^{11}B were obtained at 128 or 224 MHz all spectra were obtained at r.t. if not otherwise stated. Chemical shifts were reported in ppm according to tetramethylsilane using the solvent residual signal as an internal reference ($CDCl_3$: δ_H = 7.26 ppm, δ_C = 77.16 ppm; CD_2Cl_2: δ_H = 5.32, δ_C = 54.00, C_6D_6 δ_H = 7.16 ppm, δ_C = 128.6). Coupling constants (*J*) were given in Hz. Resonance multiplicity was described as *s* (singlet), *d* (doublet), *t* (triplet), *dd* (doublet of doublets), *dt* (doublet of triplets), *td* (triplet of doublets), *q* (quartet), *m* (multiplet) and *bs* (broad signal). Carbon spectra were acquired with a complete decoupling for the proton. ***LF NMR*** was performed at 20 °C by means of a Bruker Minispec mq 20 (0.47 T, Germany). The determination of the average water protons transverse (spin-spin) relaxation time (T_{2m}) was performed according to the CPMG sequence (Carr–Purcell–Meiboom–Gill)[1] $\{90°[-\tau-180°-\tau(echo)]_n-T_R\}$ with a 8.36 μs wide 90° pulse, τ = 250 μs, and T_R (sequences repetition rate) equal to 5 s. ***Infrared spectra*** (IR) were recorded on a Shimadzu IR Affinity 1S FTIR spectrometer in ATR mode with a diamond mono-crystal. ***Mass spectrometry:*** (i) High-resolution ESI mass spectra (HRMS) were performed on a Waters LCT HR TOF mass spectrometer in the positive or negative ion mode. (ii) High-resolution MALDI mass spectra (HRMS) were performed on a Bruker Autoflex speed MALDI-Tof, the sample was prepared with a 1:1 ratio of sample to the matrix DCTB (15 mg/mL) in CH_2Cl_2, all these analyses were carried out at Cardiff University. ***Rheological measurements*** were performed by a stress controlled rotational rheometer (Haake Mars Rheometer, 379-0200 Thermo Electron GmbH, Karlsruhe, Germany) equipped by parallel plate

geometry (PP35, ϕ= 35 mm, serrated surfaces to avoid slippage at the wall). The measuring device was kept at 10 °C inside a glass bell at saturated conditions to avoid evaporation effects.

1.2 Materials and methods

Synthesis: Chemicals were purchased from *Sigma Aldrich*, *Acros Organics*, *TCI, Apollo Scientific, Alfa Aesar* and *Fluorochem* and were used as received. Solvents were purchased from *Fisher scientific*, while deuterated solvents from *Eurisotop* and *Sigma Aldrich*. THF and toluene, were dried on a Braun MB SPS-800 solvent purification system and further dried over activated 4 Å molecular sieves. Diphenyl ether (Ph_2O) was dried over 4 Å molecular sieves. Aniline was distilled from CaH_2 under reduced pressure and stored away from light in a N_2 atmosphere. Aniline was left on CaH_2 overnight prior distillation. Low temperature baths were prepared using different solvent mixtures depending on the desired temperature: -84 °C with Ethyl acetate/liq. N_2, -10 °C with ice/NaCl and 0 °C with ice/H_2O. Anhydrous conditions were achieved by drying Schlenk tubes or 2-neck flasks by flaming with a heat gun under vacuum and purging with N_2. The inert atmosphere was maintained using N_2-filled balloons equipped with a syringe and needle that was used to penetrate the silicon stoppers used to close the flask's necks. Additions of liquid reagents were performed using plastic syringes. Degassing of solutions was performed using *Freeze-pump-thaw* (*fpt*) procedure: solutions were frozen using liquid N_2 and kept under vacuum for 10 min before thawing. Alternatively, degassing of solutions was carried out by bubbling N_2 gas under sonication for at least 10 min. Molecular sieves (4 Å) were activated by heating at 165 °C under vacuum overnight and by further heating with heat gun under vacuum immediately before use. All reactions were performed in dry conditions and under inert atmosphere unless otherwise stated. ***Solid state electrolyte:*** Lithium perchlorate $LiClO_4$ (battery grade) was obtained from *Acros* and used without further purification. TEGDME from *Sigma-Aldrich* was dried under vacuum at 70 °C for 48 h before use. CH_2Cl_2 and THF (spectroscopy grade) were purchased from *VWR* and were used as received.

2 Single-crystal X-ray Diffraction measurements

CCDC **2168896**, **2168897**, **2168899** and **2168898** contain the supplementary crystallographic data for compounds **2**, **7**, ***cc*-3** and ***ct*-3**. These data can be obtained free of charge from The Cambridge Crystallographic Data Centre via https://www.ccdc.cam.ac.uk/structures.

Single-crystal X-ray diffraction analysis - Structural characterization of 2, 7, *cc*-3 and *ct*-3.

Data collections were performed at the X-ray diffraction beamline (XRD1) of the Elettra Synchrotron, Trieste (Italy)[2]. The crystals were dipped in NHV oil (Jena Bioscience, Jena, Germany) and mounted on the goniometer head with kapton loops (MiTeGen, Ithaca, USA). Complete datasets were collected at 100 K (nitrogen stream supplied through an Oxford Cryostream 700 - Oxford Cryosystems Ltd., Oxford, United Kingdom) through the rotating crystal method. Data were acquired using a monochromatic wavelength of 0.700 Å on a Pilatus 2M hybrid-pixel area detector (DECTRIS Ltd., Baden-Daettwil, Switzerland). The diffraction data were indexed and integrated using XDS.[3] Multiple data collections had to be merged, for triclinic crystals (**7** and ***ct*-3**) to obtain complete sets, using CCP4-Aimless[4,5] code. The structures were solved by the dual space algorithm implemented in the SHELXT code.[6] Fourier analysis and refinement were performed by the full-matrix least-squares methods based on F^2 implemented in SHELXL (Version 2018/3).[7] The Coot program was used for modeling.[8] Anisotropic thermal motion refinement have been used for all atoms with full occupancies. Geometric and thermal motion parameters restraints (DFIX, DANG, SIMU) have been applied on disordered solvent molecules. Hydrogen atoms were included at calculated positions with isotropic $U_{factors} = 1.2 \cdot U_{eq}$ or $U_{factors} = 1.5 \cdot U_{eq}$ for methyl and hydroxyl groups (U_{eq} being the equivalent isotropic thermal factor of the bonded non hydrogen atom). Disordered solvent molecules have been modeled in **2** (0.16 dichloromethane/ASU), ***cc*-3** (1.75 MeOH/ASU + 0.25 water/ASU) and ***ct*-3** (0.35 CH_2Br_2/ASU) structures. An additional disordered solvent contribution has been identified in ***cc*-3**, where MeOH and H_2O fill inner parts of crystal channels without specific intermolecular contacts with the ordered borazine packing scaffold. The corresponding, not construable residual electron density, couldn't be modeled and has been removed with Platon SQUEEZE[9] routine (120 e^-/cell squeezed, corresponding to additional ~5.5 MeOH and ~1.5 H_2O molecules/cell in 420 $Å^3$ volume voids). Crystals of **2** show merohedral twinning (direct lattice two-fold twinning axis [100]), with a twin fraction of 45%, identified using Platon TWINROTMAT algorithm.[10] Pictures were prepared using Ortep3[11] and Pymol[12] software. Essential crystal and refinement data are reported below (Table S1).

7 and ***ct*-3** form triclinic *P* -1 crystals with one crystallographically independent molecule in the asymmetric units (ASUs). **2** and ***cc*-3** crystallize in trigonal *R*-3 and hexagonal $P6_3$ space groups respectively, matching crystallographic and molecular threefold axis. Their ASUs therefore contain $^1/_3$ of a full borazine; for **2** an additional complete moiety is present (Figure S2A).

All the borazine cores are almost perfectly flat (R.M.S.D. of B_3N_3 ring from the core mean plane is 0.03(3) Å). Phenyl groups attached to nitrogen adopt a conformation perpendicular to the borazine core to minimize steric effects, in all the molecules. Equivalent substituents adopt practically identical conformations (R.M.S.D. between overlapped models of **2** and **7** is 0.37 Å) with minor rearrangements on peripheral phenyl substituents (R.M.S.D. between common branches of ***cc*-3** and ***ct*-3** is 1.47 Å - Figure S3).

All the crystals show weak hydrophobic packing interactions with limited π···π stacking (Figure S4-S7); CH···π interactions are more frequently found, with remarkably short and well oriented contacts among neighbor **2** molecules ($d_{C\equiv CH\cdots Phenyl}$ = 3.433(4) or 3.727(3) Å). Alkynyl substituents are not highly sterically demanding therefore **7** crystal packing doesn't show any void (Packing Index = 68.1%[9]). Small voids, filled with solvent molecules, are available in **2** (Packing Index = 62.7%[9]) and ***ct*-3** (Packing Index = 64.5%[9]) structures, while huge solvent channels are available in ***cc*-3** crystal packing (Packing Index = 48.7%[9]). ***cc*-3** channels occupy 33% of the unit cell volume, are aligned with crystallographic *c* axis and are filled with partially ordered H_2O and MeOH molecules (Figure S6).

2.1 Powder XRD Results on the Crystallized 3 Sample

Experimental method. Crystallization of **3** was carried out by diffusion of MeOH into a solution of **3** in CH_2Br_2. Powder XRD data were recorded for the crystallized sample at 21 °C on a Bruker D8 diffractometer (Ge-monochromated CuKα_1 radiation; transmission mode; Våntec detector covering 3° in 2θ; 2θ range, 4° to 50°; step size, 0.016°; data collection time, 119.5 h).

Results. Powder XRD data recorded for samples of **3** and the **BN-polymer** material prepared directly from the chemical reactions shown in Scheme 1 indicate that both materials are amorphous. To gain insights into the stereochemistry of the chemical reaction (for example, to establish whether the spatial distribution of the substituents bonded to the boron atoms of the B_3N_3 rings corresponds to the ***cc*** isomer, the ***ct*** isomer, or a mixture of the ***cc*** and ***ct*** isomers), the sample of **3** was crystallized from CH_2Br_2, as described above. The structural properties of the bulk crystallized sample of **3** were then investigated by analysis of the powder XRD data. Figure S1 shows the results from Le Bail fitting[13] of the powder XRD data for the crystallized sample of **3**, starting from the unit cell of the triclinic form of **3** determined from single-crystal XRD data, which contains the ***ct*** isomer as a solvate with CH_2Br_2 (see Table S1). The Le Bail fitting procedure led to a good-quality fit, with final refined unit cell parameters: a = 12.4760(12) Å, b = 16.3471(15) Å, c = 21.5362(20) Å, α = 71.848(5)°, β = 88.747(7)°, γ = 83.658(7)°, V = 4147.7(9) Å^3. The unit cell volume is larger by *ca*. 0.7% than the unit cell determined for the same material from single-crystal XRD data, which is explained by the fact that the single-crystal XRD data collection was carried out at 100 K whereas the powder XRD data were recorded at 294 K). From the good quality of the Le Bail fit (Figure S1), we conclude that the only detectable crystalline phase is the $P\bar{1}$ phase of the CH_2Br_2 solvate of **3**, which contains the ***ct*** isomer of **3**. The minor discrepancies evident in the difference plot in Figure S1 may be attributed to small errors in the baseline and lineshape. We note that other crystalline phases may also be present (such as the $P6_3$ phase, which contains the ***cc*** isomer of **3**), but are below the detection limit, which is estimated to be several percent given the relatively poor signal-to-noise level in the powder XRD data.

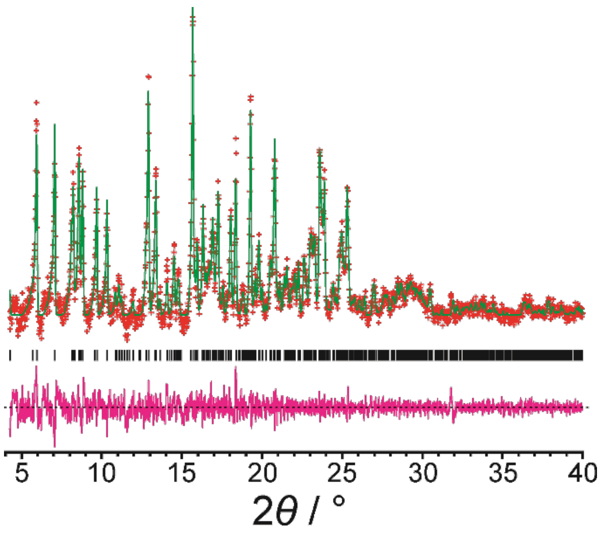

Figure S1. Le Bail fitting of the powder XRD data (background subtracted) recorded for the sample of **3** crystallized from CH_2Br_2 (red + marks, experimental data; green line, calculated data; magenta line, difference plot; black tick marks, predicted peak positions).

Table S1. Crystallographic data and refinement details for **2**, **7**, ***cc*-3** and ***ct*-3**.

	**2**·1/8CH_2Cl_2 [$C_{24}H_{18}B_3N_3$·1/8CH_2Cl_2]	**7** [$C_{27}H_{24}B_3N_3$]	***cc*-3**·8CH_3OH·1/2H_2O [$C_{108}H_{78}B_3N_3$·8CH_3OH·1/2H_2O]	***ct*-3**·1/3CH_2Br_2 [$C_{108}H_{78}B_3N_3$·1/3CH_2Br_2]
CCDC Number	**2168896**	**2168897**	**2168899**	**2168898**
Chemical Formula	$C_{24.12}H_{18.25}B_3Cl_{0.25}N_3$	$C_{27}H_{24}B_3N_3$	$C_{116}H_{113}B_3N_3O_{9.5}$	$C_{108.35}H_{78.7}B_3Br_{0.7}N_3$
Formula weight (g/mol)	391.46	422.92	1733.52	1511.01
Temperature (K)	100(2)	100(2)	100(2)	100(2)
Wavelength (Å)	0.700	0.700	0.700	0.700
Crystal system	Trigonal	Triclinic	Hexagonal	Triclinic
Space Group	*R* -3	*P* -1	*P* 6_3	*P* -1
Unit cell dimensions	*a* = 33.662(5) Å	*a* = 9.992(2) Å	*a* = 25.694(4) Å	*a* = 12.429(2) Å
	b = 33.662(5) Å	*b* = 11.382(2) Å	*b* = 25.694(4) Å	*b* = 16.259(3) Å
	c = 13.762(3) Å	*c* = 11.726(2) Å	*c* = 9.446(2) Å	*c* = 21.464(4) Å
	α = 90°	α = 62.25(3)°	α = 90°	α = 71.24(3)°
	β = 90°	β = 84.02(3)°	β = 90°	β = 88.86(3)°
	γ = 120°	γ = 86.29(3)°	γ = 120°	γ = 83.77(3)°
Volume (Å^3)	13505(5)	1173.6(5)	5400.6(19)	4082.2(16)
Z	24	2	2	2
Density (calculated) (g·cm^{-3})	1.155	1.197	1.066	1.229
Absorption coefficient (mm^{-1})	0.091	0.066	0.064	0.395
F(000)	4878	444	1842	1579
Theta range for data collection	1.19° to 29.97°	2.02° to 30.97°	2.31° to 30.96°	0.99° to 30.00°
Index ranges	$-45 \le h \le 46$	$-14 \le h \le 14$	$-37 \le h \le 37$	$-17 \le h \le 16$
	$-47 \le k \le 47$	$-16 \le k \le 16$	$-37 \le k \le 34$	$-23 \le k \le 23$
	$-17 \le l \le 17$	$-16 \le l \le 16$	$-13 \le l \le 13$	$-30 \le l \le 30$
Resolution (Å)	0.70	0.70	0.68	0.70
Reflections collected	44602	66640	61077	82600
Independent reflections (data with I>2σ(I))	8874 (6292)	7130 (7017)	11870 (6997)	23937 (18973)
Data multiplicity (max resltn)	4.88 (4.01)	9.31 (8.44)	9.70 (8.32)	3.31 (2.96)
I/σ(I) (max resltn)	12.89 (3.02)	59.41 (50.44)	10.41 (2.25)	7.00 (4.76)
R_{merge} (max resltn)	0.0604 (0.3447)	0.0264 (0.0286)	0.1111 (0.6042)	0.0454 (0.1633)
Data completeness (max resltn)	97.2% (92.8%)	99.9% (99.9%)	99.9% (99.6%)	96.0% (91.9%)
Refinement method	Full-matrix least-squares on F^2	Full-matrix least-squares on F^2	Full-matrix least-squares on F^2	Full-matrix least-squares on F^2
Data / restraints / parameters	8874 / 5 / 328	7130 / 0 / 301	11870 / 23 / 397	23937 / 17 / 1050
Goodness-of-fit on F^2	1.014	1.036	1.000	1.021
Δ/σ_{max}	0.001	0.005	0.006	0.000
Final R indices [I>2σ(I)]	R_1 = 0.0597, wR_2 = 0.1407	R_1 = 0.0417, wR_2 = 0.1138	R_1 = 0.0683, wR_2 = 0.1581	R_1 = 0.0765, wR_2 = 0.2063
R indices (all data)	R_1 = 0. 0927, wR_2 = 0.1616	R_1 = 0.0420, wR_2 = 0.1141	R_1 = 0.1237, wR_2 = 0.1861	R_1 = 0.0932, wR_2 = 0.2229
Largest diff. peak and hole (eÅ^{-3})	0.336 and -0.239	0.398 and -0.364	0.264 and -0.242	1.105 and -1.140
R.M.S. deviation from mean (eÅ^{-3})	0.049	0.049	0.049	0.075

$$R_1 = \Sigma \,||Fo|-|Fc|| \,/\, \Sigma\, |Fo|;\ wR_2 = \{\Sigma\, [w(Fo^2 - Fc^2)^2] \,/\, \Sigma\, [w(Fo^2)^2]\}^{1/2}$$

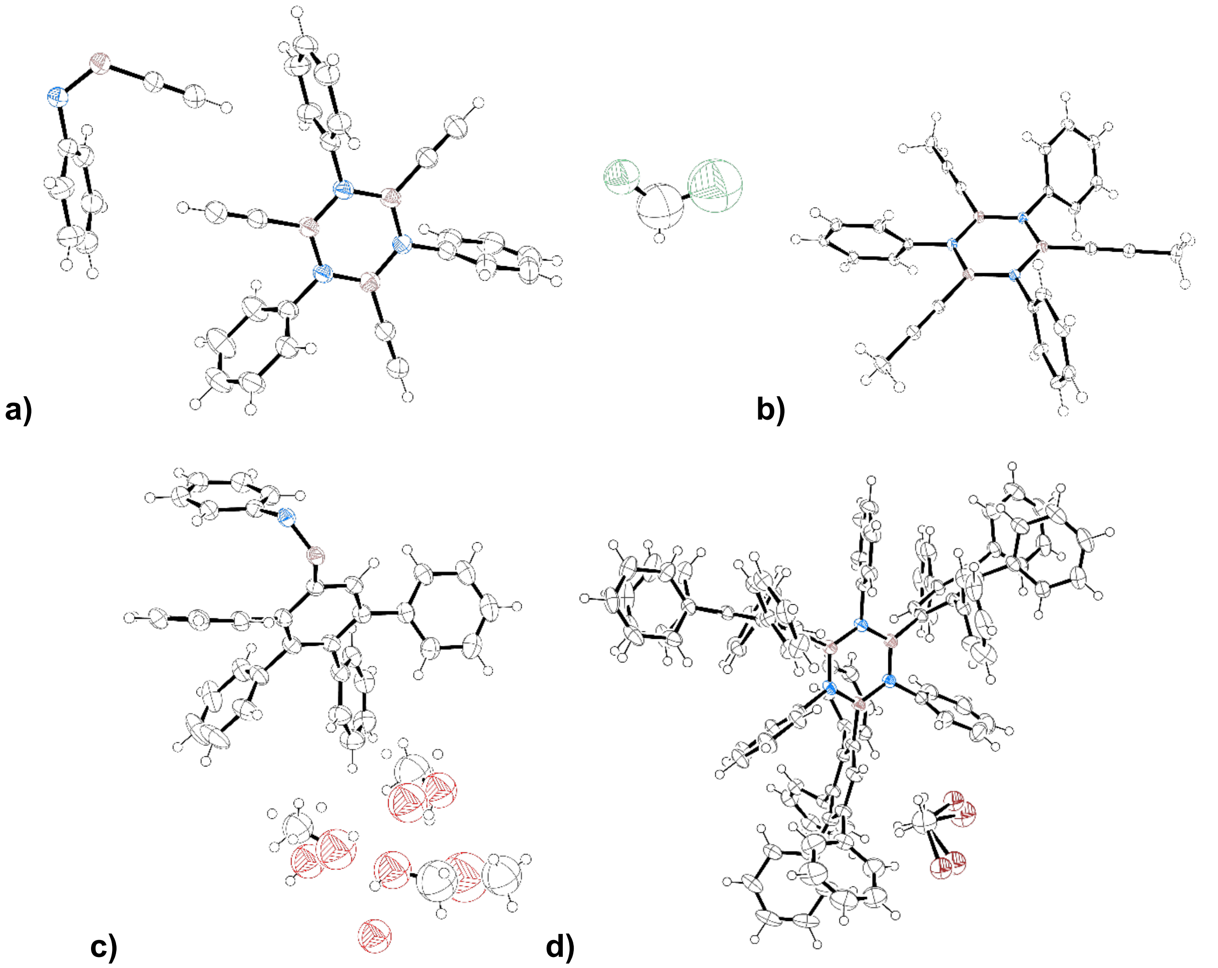

Figure S2. Ellipsoids representation of ASU contents (50% probability) for **2** (A), **7** (B), ***cc*-3** (C) and ***ct*-3** (D).

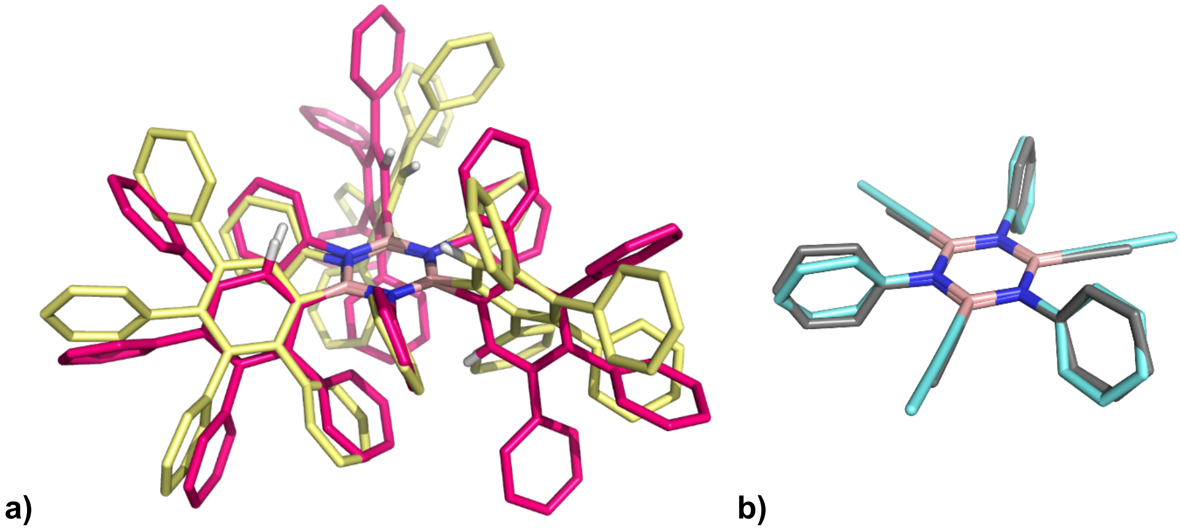

Figure S3. Overlapped models of A) ***cc*-3** (yellow sticks) with ***ct*-3** (hot pink sticks) or B) **2** (grey sticks) and **7** (cyan sticks). Hydrogens representative of the stereochemistry are shown with white sticks – all the others are omitted for clarity. R.M.S.D. between BN core and equivalent substituents is 1.47 Å in A) and 0.37 Å in B).

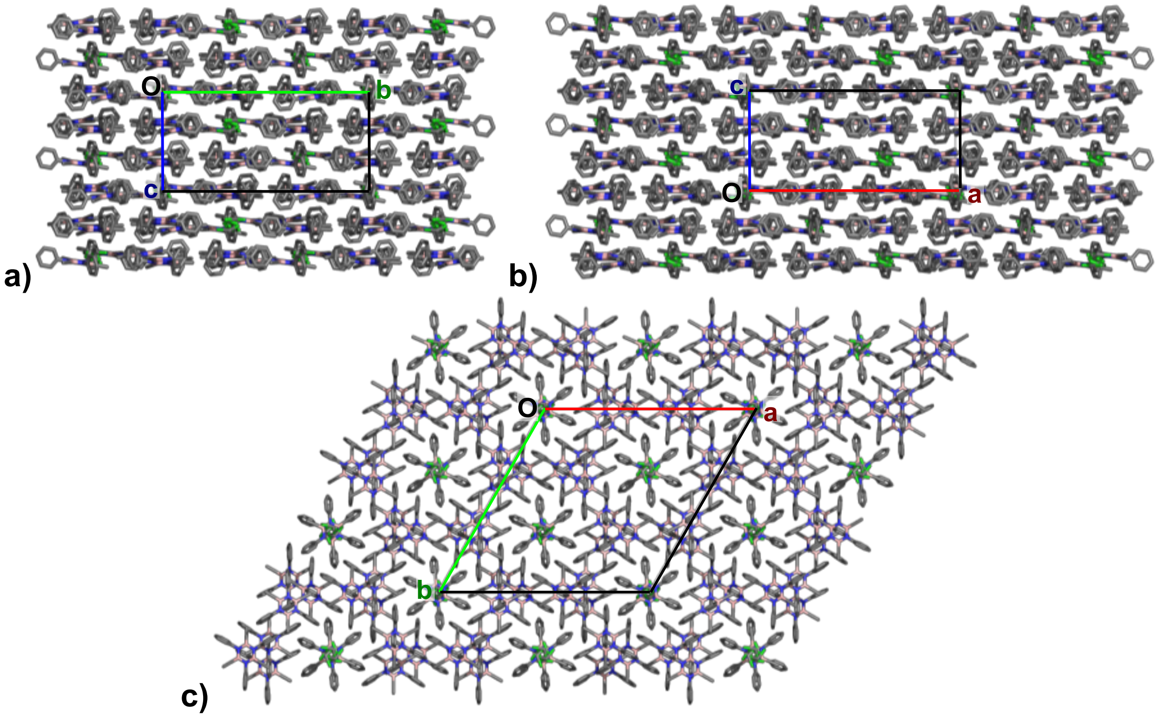

Figure S4. 2 crystal packing views along crystallographic *a*, *b* and *c* axis. Hydrogens omitted for clarity.

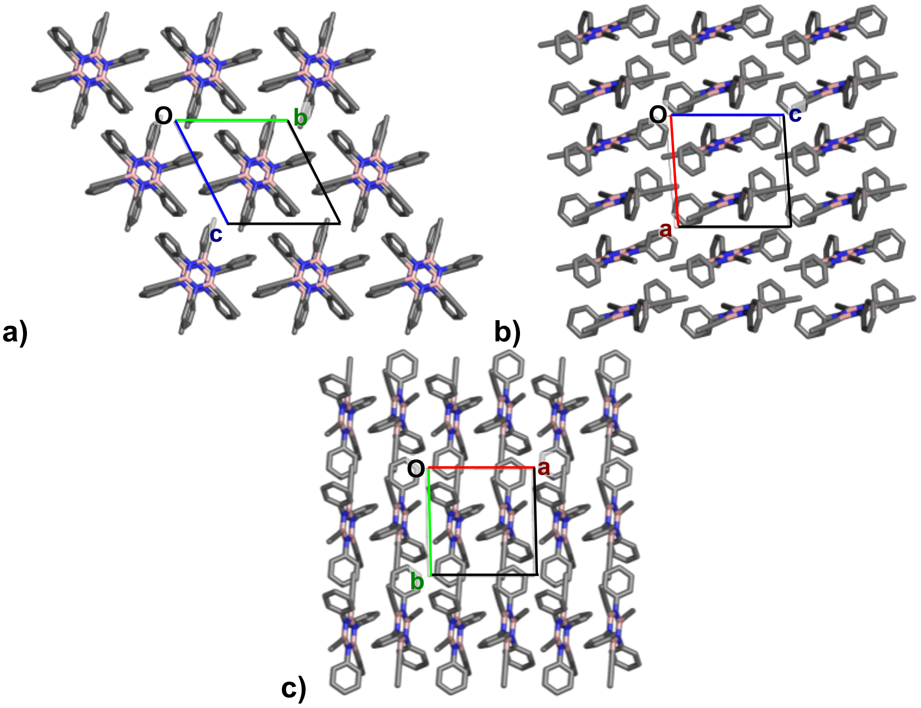

Figure S5. 7 crystal packing views along crystallographic *a*, *b* and *c* axis. Hydrogens omitted for clarity.

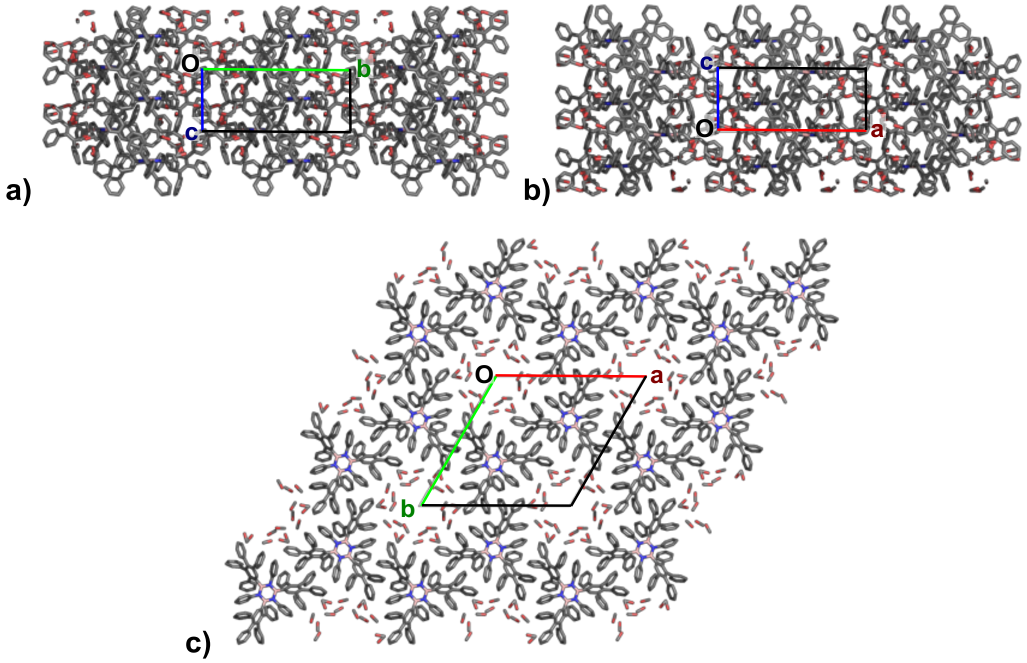

Figure S6. ***cc*-3** crystal packing views along crystallographic *a*, *b* and *c* axis. Hydrogens omitted for clarity.

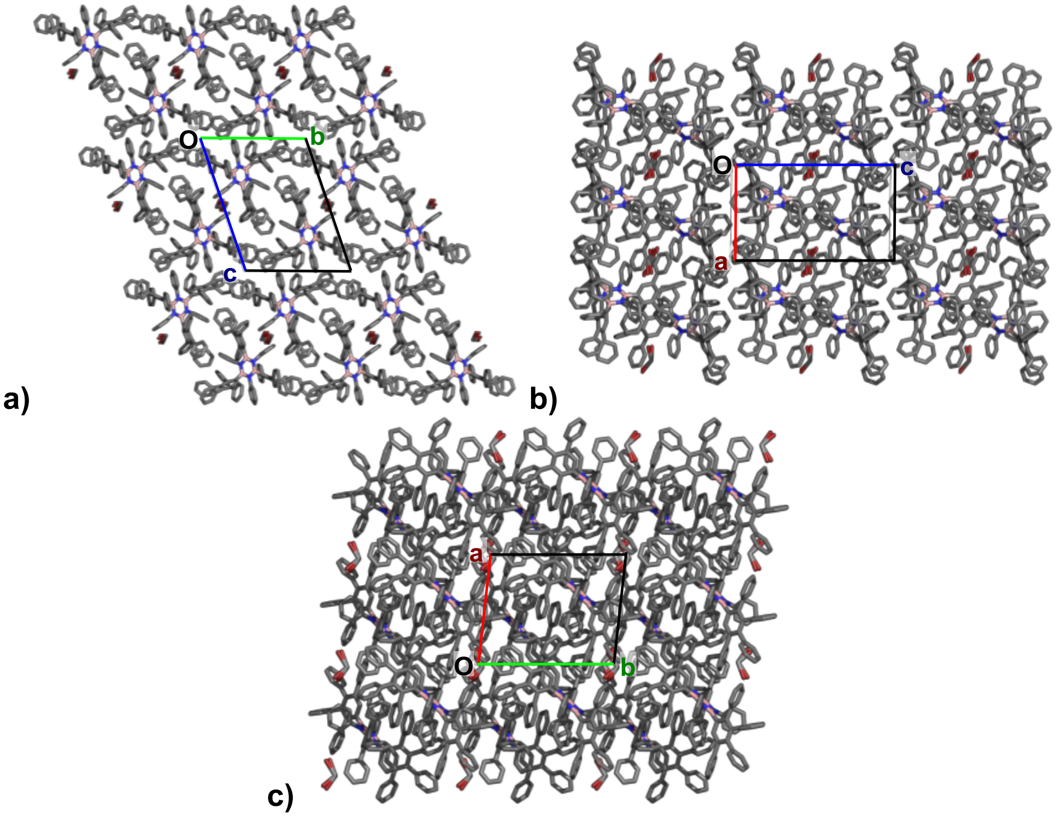

Figure S7. ***ct*-3** crystal packing views along crystallographic *a*, *b* and *c* axis. Hydrogens omitted for clarity.

3 Synthetic procedures and spectral data

Synthesis of *B,B',B"*-Triethynyl-*N,N',N"*-tri(phenyl)-borazine (2)

The product is moisture sensitive

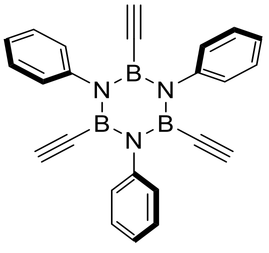

In a flame dried 20 mL Schlenk flask, freshly distilled aniline (0.49 mL, 5.37 mmol) and dry toluene (7 mL) were added. The solution was then cooled to 0 °C, BCl_3 (1 M in heptane, 6.44 mL) added dropwise, and the resulting suspension stirred for 10 min. The Schlenk flask was then equipped with a condenser connected to a $CaCl_2$ trap and the reaction refluxed for 18 h. The condenser was then exchanged with a septum and the white suspension degassed 5 times following the *freeze-pump-thaw* procedure to remove residual HCl. The degassed mixture was cooled to 0°C and HC≡CMgBr (0.5 M in THF, 12.0 mL) added dropwise. The resulting pale-yellow solution was allowed to reach r.t. and stirred for 3 h, dry CH_2Cl_2 (50 mL) added, and the reaction mixture filtered on a silica plug washing thoroughly with dry CH_2Cl_2. Evaporation of the solvent gave **2** as a pale-yellow powder (478 mg, 70%). mp: 260-262 °C. ^1H NMR: (300 MHz, CD_2Cl_2) δ: 7.38-7.33 (*m*, 6 H), 7.28-7.22 (*m*, 9 H), 2.47 (*s*, 3 H). $^{13}C\{^1H\}$ NMR (75 MHz, $CDCl_3$) δ: 147.00, 128.75, 126.26, 98.31. Signal at 128.75 consists of 2C overlapped, 1 ethynyl signal missing due to ^{11}B quadrupolar relaxation. $^{11}B\{^1H\}$ NMR (128 MHz, $CDCl_3$) δ: 26.26. IR (ATR) ν (cm^{-1}): 3056, 3032, 2355, 1420, 1204, 1129, 900, 754, 694, 411, 408, 404. EI^+ HRMS: $[M]^+$ calc. for $[C_{24}H_{18}B_3N_3]^+$: 381.1780; found: 381.1756. Crystals suitable for X-Ray diffraction analysis were obtained from slow evaporation of a **2** solution in 1/1 petroleum ether (PE)/CH_2Cl_2.

Synthesis of *B,B',B"*-Tri(3',4'-diphenyl-1,1':2',1"-terphenyl)-*N,N',N"*-tri(phenyl)-borazine (3, *cc* and *ct* isomers).

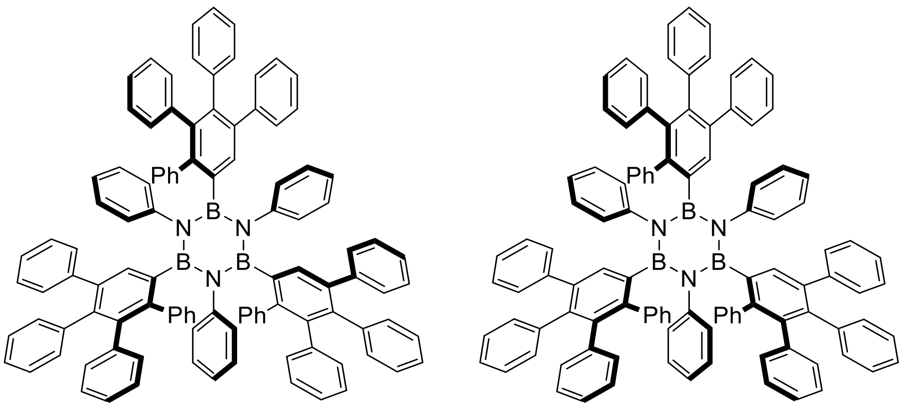

In a flame dried two necked flask, borazine **2** (50 mg, 0.13 mmol) was added along with 2,3,4,5-tetraphenylcyclopenta-2,4-dien-1-one (201 mg, 0.52 mmol). The solids were suspended in dry Ph_2O (1 mL) and the resulting black suspension degassed with N_2 bubbling under sonication for 30 min. The mixture was heated under N_2 at 220 °C for 18 h. The resulting black solution was precipitated from cold petrol and filtered to give a light purple solid. Silica gel column chromatography (eluent: PE/CH_2Cl_2 80:20 to 60:40) gave **3** as a white fluffy powder (91 mg, 47%). mp: >300 °C. ^{1}H NMR: (500 MHz, $CDCl_3$) δ: 7.17-7.05 (*m*, 22 H), 6.92-6.65 (*m*, 43 H), 6.65-5.97 (*m*, 13 H). ^{13}C{^{1}H} NMR (126 MHz, $CDCl_3$) δ: 146.18, 146.10, 145.10, 142.84, 142.65, 142.45, 142.19, 142.11, 142.06, 141.74, 141.64, 141.54, 141.10, 140.79, 140.56, 140.44, 140.42, 140.34, 139.51, 139.20, 138.96, 138.89, 138.59, 138.42, 138.26, 137.46, 135.63, 135.19, 135.02, 132.01, 131.68, 131.65, 131.59, 131.47, 131.41, 131.34, 131.14, 131.10, 130.18, 129.96, 129.87, 128.74, 128.53, 127.35, 127.31, 127.09, 126.97, 126.77, 126.56, 126.50, 126.42, 126.34, 126.30, 126.10, 125.88, 125.76, 125.68, 125.28, 125.23, 125.02, 124.97, 124.34, 124.04, 123.71. ^{11}B{^{1}H} NMR (224 MHz, C_6D_6 δ: 37.71. IR (ATR) ν (cm^{-1}): 3055, 3024, 1597, 1577, 1491, 1358, 1321, 1072, 1027, 908, 756, 725, 694, 533, 419, 406. AP$^+$ HRMS: [M+H]$^+$ calc. for [$C_{108}H_{79}B_3N_3$]$^+$: 1450.6548; found: 1450.6578. Crystals suitable for X-Ray diffraction analysis were obtained from slow diffusion of MeOH in a CH_2Br_2 solution of **3**.

Synthesis of 4,4'-Bis(phenylethynyl)-1,1'-biphenyl (4)

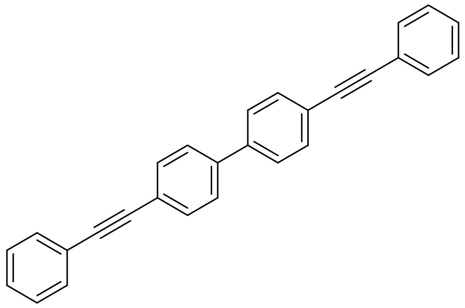

4,4'-diiodo-1,1'-biphenyl (4.0 g, 9.85 mmol) was added in a Schlenk flask along with [Pd(PPh_3)$_2$$Cl_2$] (138 mg, 0.2 mmol) and CuI (38 mg, 0.2 mmol). The solids were then suspended in 50 mL of a 1/1 THF/triethylamine mixture and the resulting suspension degassed 3 times using *freeze-pump-thaw* procedure. Phenylacetylene (2.4 mL, 21.7 mmol) was then added, and the reaction stirred at r.t. for 3 h. The resulting suspension was filtered on a glass frit and washed with MeOH to give **4** as a grey powder (3.45 g, 99 %). mp: 246-248 °C. ^{1}H NMR: (300 MHz, $CDCl_3$) δ: 7.61 (*s*, 8 H), 7.58-7.54 (*m*, 4 H), 7.39-7.35 (*m*, 6 H). ^{13}C{^{1}H} NMR (100 MHz, $CDCl_3$) δ: 140.19, 132.27, 131.79, 128.53, 128.50, 127.04, 123.38, 122.74, 90.53, 89.37. IR (ATR) ν (cm^{-1}): 3086, 3051, 2965, 2359, 2218, 1593, 1499, 1439, 1069, 1024, 1001, 918, 822, 750, 718, 689, 538, 515, 471. ASAP$^+$ HRMS: [M+H]$^+$ calc. for [$C_{28}H_{19}$]$^+$: 355.1481; found: 355.1481.

Synthesis of 2,2'-([1,1'-Biphenyl]-4,4'-diyl)bis(1-phenylethane-1,2-dione) (5)

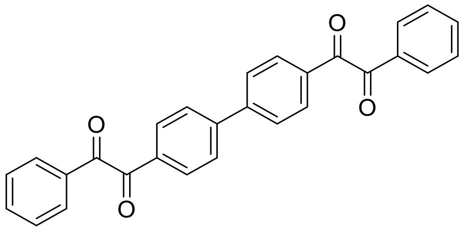

In a 500 mL round bottom flask **4** (1.0 g, 2.82 mmol) was added and suspended in an acetone/AcOH (200 mL/2 mL) mixture. $KMnO_4$ (2.2 g, 14.12 mmol) was then added in one portion. The reaction was then equipped with a condenser and stirred at 60 °C for 6 h. The solvent was removed under reduced pressure and the resulting solid filtered on a silica plug (eluent: CH_2Cl_2 100%). Evaporation of the solvent gives **5** (1.14 g, 97 %) as a bright yellow solid. mp: 196-198 °C. ^{1}H NMR: (300 MHz, $CDCl_3$) δ: 8.09 (*d*, J = 8.5 Hz, 4 H), 8.01 (*d*, J = 8.5 Hz, 4 H), 7.76 (*d*, J = 8.5 Hz, 4 H), 7.68 (*t*, J = 7.5 Hz, 2 H), 7.53 (*t*, J = 7.5 Hz, 4 H). ^{13}C{^{1}H} NMR (75 MHz, $CDCl_3$) δ: 194.37, 193.98, 145.83, 135.18, 133.03, 132.85, 130.77, 130.12, 129.23, 128.14. IR (ATR) ν (cm^{-1}): 3084, 3067, 1661, 1593, 1553, 1447, 1400, 1317, 1300, 1209, 1169, 1001, 872,

824, 793, 748, 719, 704, 685, 648 634, 490. ASAP$^+$ HRMS: $[M+H]^+$ calc. for $[C_{28}H_{19}O_4]^+$: 419.1278; found: 419.1284.

Synthesis of 4,4'-([1,1'-Biphenyl]-4,4'-diyl)bis(2,3,5-triphenylcyclopenta-2,4-dien-1-one) (6)

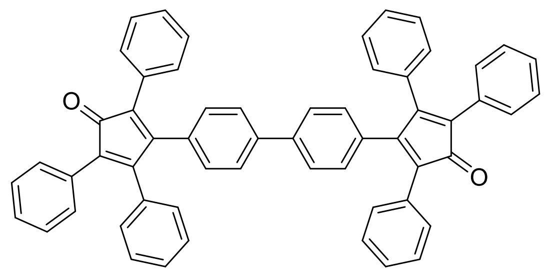

In a 50 mL round bottom flask equipped with a condenser, **5** (1.0 g, 2.4 mmol) was added and dissolved in refluxing EtOH (10 mL). 1,3-Diphenylacetone (1.1 g, 5.0 mmol) was then added portion-wise to the yellow solution at reflux. A KOH solution (135 mg in 2 mL of EtOH) was then added and after 10 min a brownish suspension was formed. The flask was cooled to -10 °C for 1 h and the reaction mixture filtered on a glass frit and washed with cold MeOH to give the desired product **6** as a brown solid (960 mg, 52 %). mp: >300 °C. ^{1}H NMR: (300 MHz, $CDCl_3$) δ: 7.43 (*d*, J = 8.4 Hz, 4 H), 7.29-7.27 (*m*, 20 H), 7.25-7.20 (*m*, 6 H), 7.01-6.98 (*m*, 8 H). $^{13}C\{^1H\}$ NMR (126 MHz, $CDCl_3$) δ: 200.27, 154.39, 153.98, 140.07, 133.28, 132.50, 130.94, 130.82, 130.32, 130.29, 130.21, 129.49, 128.69, 128.26, 128.24, 128.18, 127.69, 127.65, 126.35, 125.70, 125.67. IR (ATR) ν (cm^{-1}): 3055, 3030, 2911, 1707, 1601, 1490, 1443, 1354, 1304, 693, 419, 412. AP$^+$ HRMS: $[M+H]^+$ calc. for $[C_{58}H_{39}O_2]^+$: 767.2945; found: 767.2959.

Synthesis of BN-Polymer*

* The quality of the starting borazine and dry conditions are fundamental to form the polymeric material, if the borazine is partially degraded or moisture is present only soluble material is formed.

In a flame dried 3 necked flask **2** (300 mg, 0.79 mmol) was added along with **6** (905 mg, 1.18 mmol) and the flask purged 3 times with N_2. Anhydrous Ph_2O (12 mL) was then added, and the resulting brown suspension degassed with N_2 bubbling under sonication for 20 min. The reaction was then stirred under N_2 at 230 °C (as soon as the reaction reaches 220 °C a strong gas evolution takes place). After approximately 1 h the reaction turns into a red gel and is stirred for further 16 h. The gelified solution is then allowed to reach room temperature, dropped into cold PE, and filtered. The resulting material was transferred to a falcon tube, suspended in CH_2Cl_2, and sonicated for 10 min., followed by addition of PE and centrifugation at 5000 rpm for 5 min. This procedure was repeated for further 2 times. The reddish powder was then added in a flame dried microwave vial and suspended in Ph_2O (7 mL). The vial was sealed, and the solution degassed with N_2 bubbling under sonication for 10 min (forms a gel). Phenylacetylene (1.15 mL, 10.4 mmol) was then added, and the reaction heated under N_2 at 230 °C for 4 h (red colour disappears). After this time the reaction was

again poured into cold PE and washed 3 times by centrifugation with CH_2Cl_2 (as reported in the previous step) and 1 time each with acetone, MeOH and Et_2O. After drying under vacuum, **BN-Polymer** was isolated as a beige solid (600 mg, 52%). IR (ATR) ν (cm^{-1}): 3211, 3051, 3022, 1597, 1489, 1356, 1319, 1155, 1109, 1072, 1026, 1007, 910, 831, 785, 756, 694, 532, 469, 457, 447, 417, 407, 403.

Synthesis of *B,B',B''*-tripropynyl-*N,N',N''*-triphenyl-borazine (7)

The product is moisture sensitive

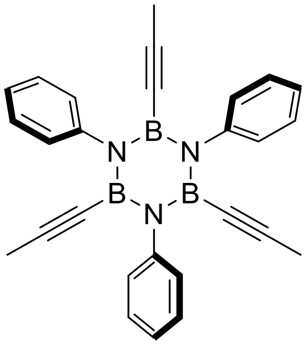

In a flame dried 20 mL Schlenk-type flask, aniline (0.49 mL, 5.37 mmol) and dry toluene (7 mL), were added. The solution was then cooled to 0 °C and BCl_3 (1 M in heptane, 6.44 mL) added dropwise. The resulting suspension was stirred under refluxing conditions for 18 h, then cooled down to r.t. and subjected to 5 *freeze-pump-thaw* cycles to remove the excess HCl. The reaction mixture was then cooled down to 0 °C and propynyl magnesium bromide (0.5 M in THF, 12 mL) added dropwise. The resulting pale-yellow solution was allowed to reach r.t. and stirred for 3 h, dry CH_2Cl_2 (50 mL) added, and the reaction filtered on a silica plug washing thoroughly with dry CH_2Cl_2. Evaporation of the solvent gives **7** as a pale-yellow powder (507 mg, 67%). mp: 244-246 °C. ^{1}H NMR: (300 MHz, $CDCl_3$) δ: 7.32-7.27 (*m*, 6 H), 7.20-7.14 (*m*, 9 H), 1.43 (*s*, 9 H). ^{13}C{^{1}H} NMR (126 MHz, $CDCl_3$) δ: 147.67, 128.53, 127.83, 124.96, 108.08, 4.76. 1 ethynyl signal missing due to ^{11}B quadrupolar relaxation. ^{11}B NMR (160 MHz, $CDCl_3$) δ: 26.17. IR (ATR) ν (cm^{-1}): 3062, 3033, 2918, 2197, 1597, 1494, 1377, 1026, 765, 713, 694, 616, 560. AP^+-HRMS: $[M+H]^+$ calc. for $[C_{27}H_{25}{}^{10}B_2{}^{11}BN_3]^+$: 422.2395; found: 422.2406. Crystals suitable for diffraction were obtained from slow evaporation of a $CHCl_3$ solution of **7**.

4 NMR-HRMS spectroscopic characterization (^{1}H, ^{13}C, ^{11}B, HRMS)

4.1 **Characterization of *B,B',B"*-Triethynyl-*N,N',N"*-tri(phenyl)-borazine (2)**

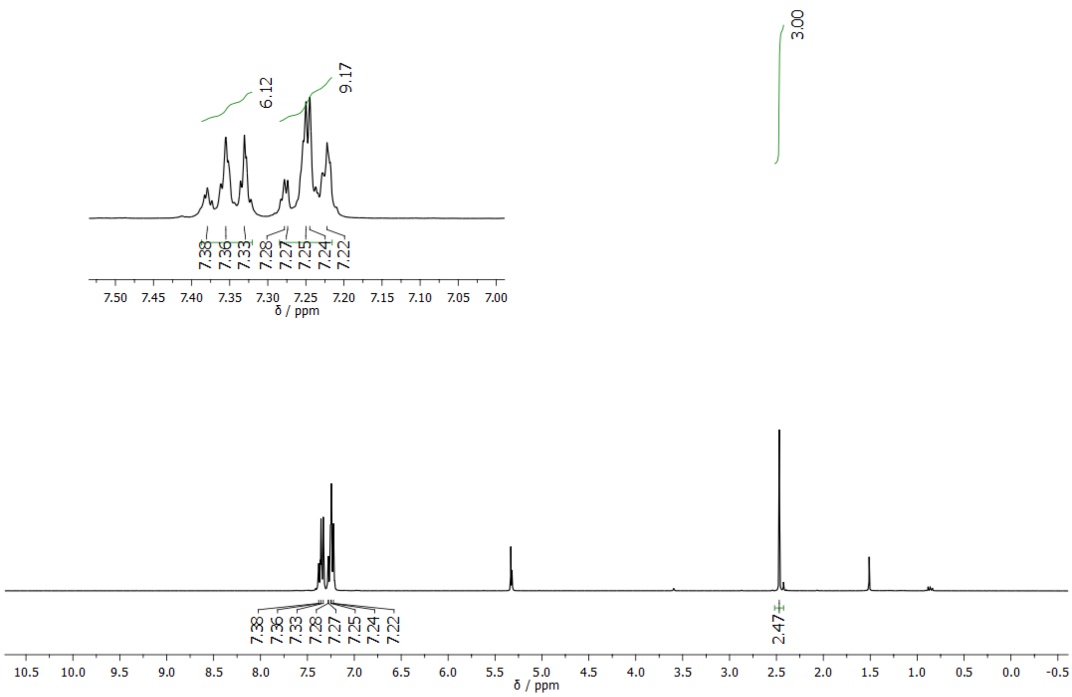

Figure S8. 300 MHz ^{1}H-NMR of **2** in CD_2Cl_2.

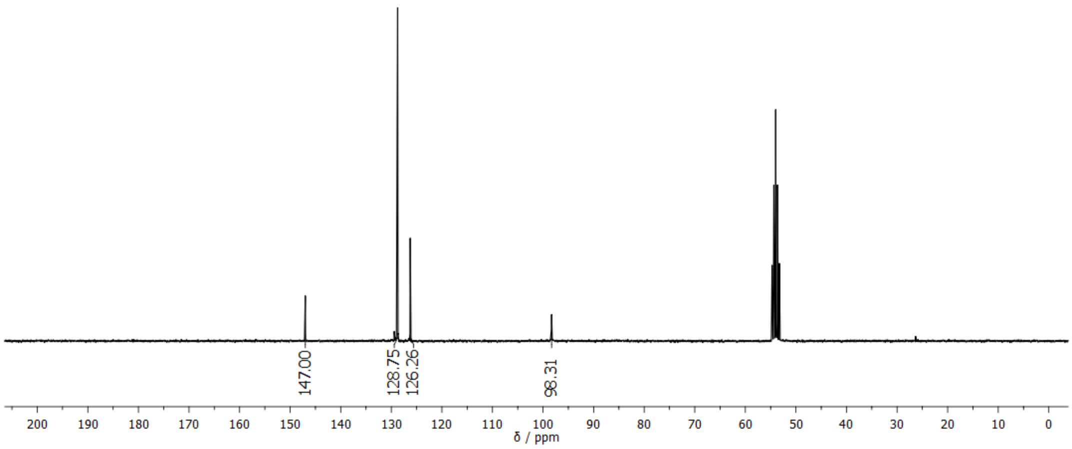

Figure S9. 75 MHz ^{13}C-NMR of **2** in CD_2Cl_2.

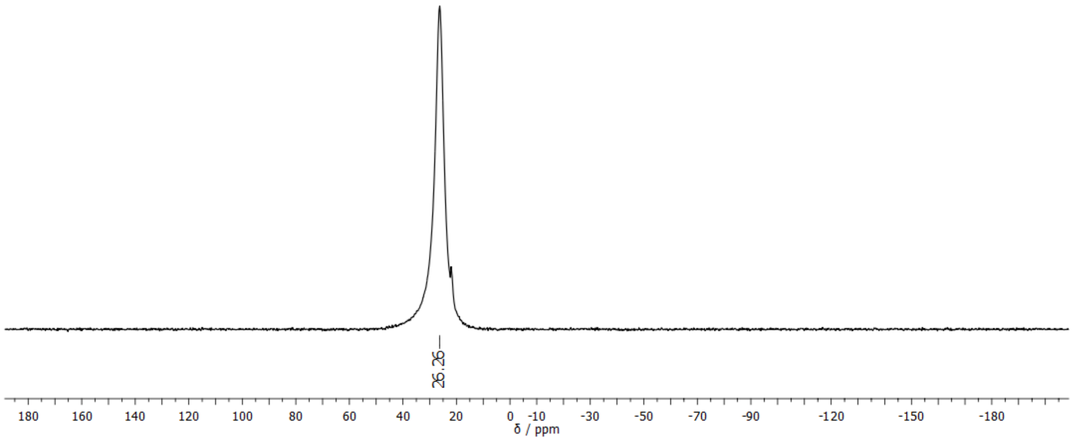

Figure S10. 160 MHz ^{11}B-NMR of **2** in $CDCl_3$.

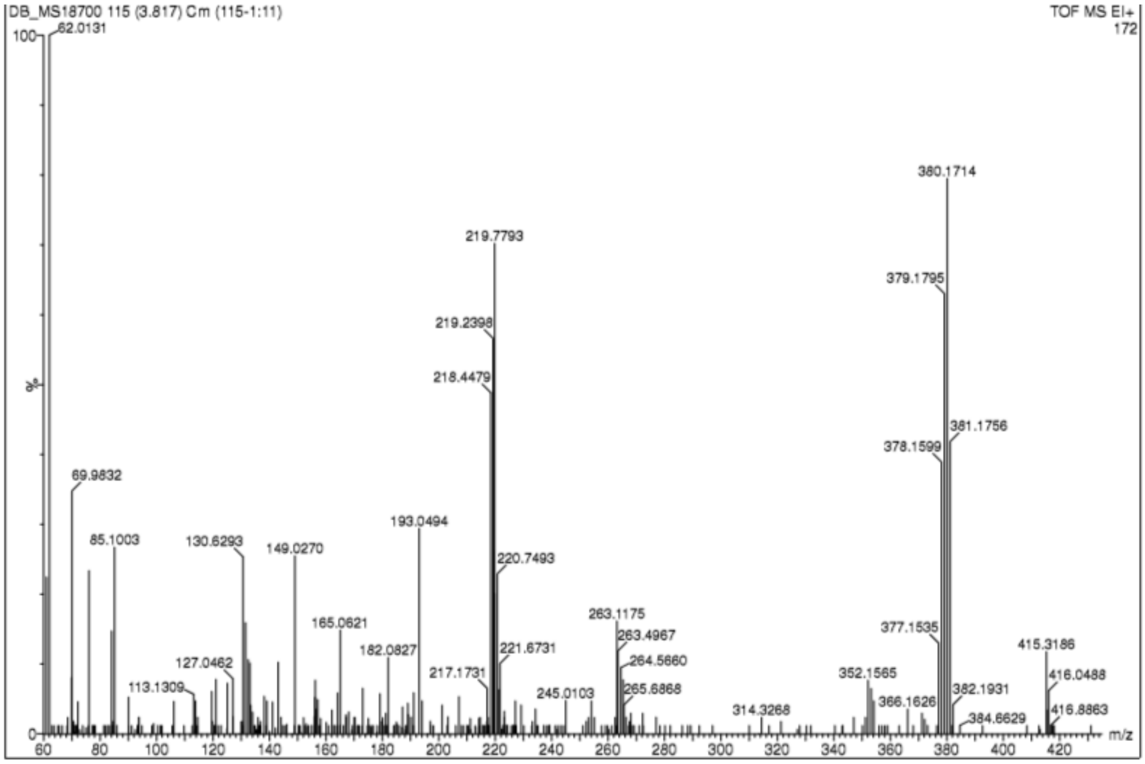

Figure S11. EI^+ HR-MS of **2**.

4.2 **Characterization of *B,B',B"*-Tri(3',4'-diphenyl-1,1':2',1''-terphenyl)-*N,N',N"*-tri(phenyl)-borazine (3, *cc* and *ct* isomers).**

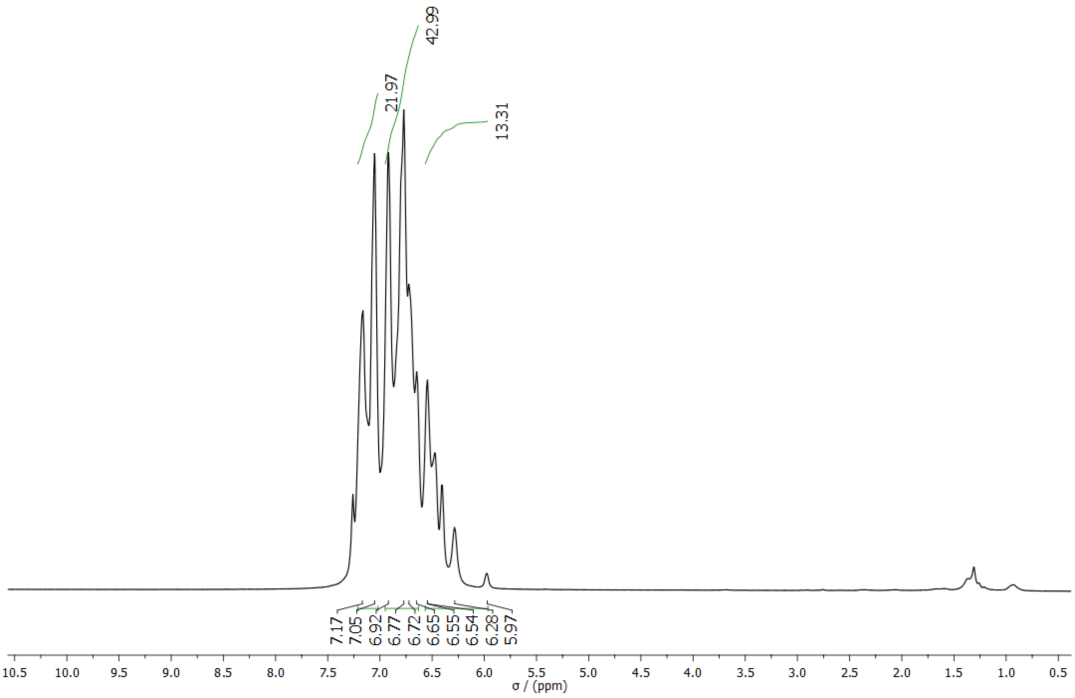

Figure S12. 500 MHz ^{1}H-NMR of **3** in $CDCl_3$.

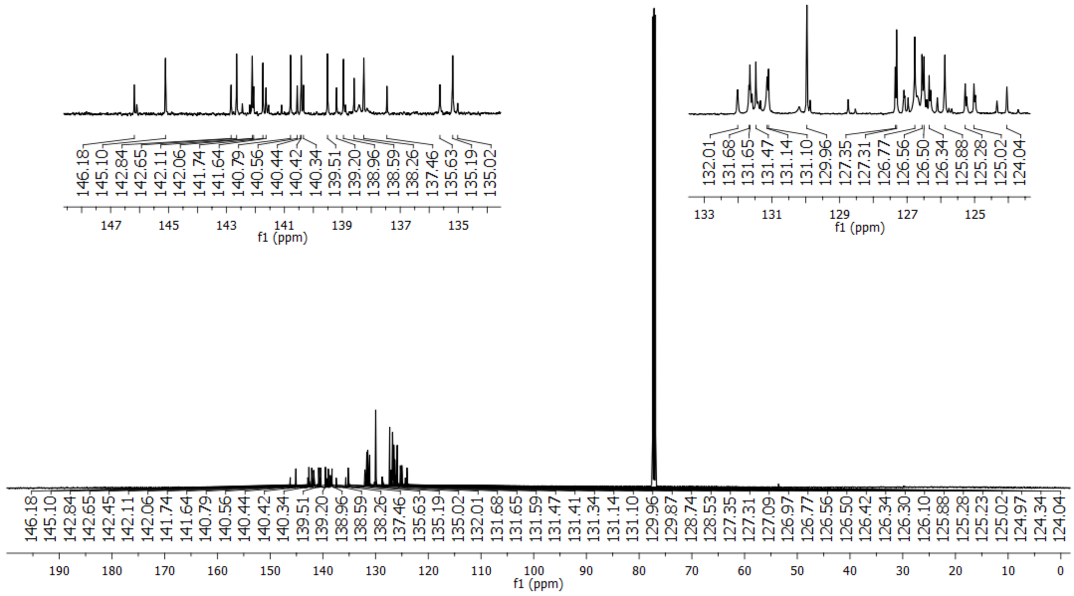

Figure S13. 126 MHz ^{13}C-NMR of **3** in $CDCl_3$.

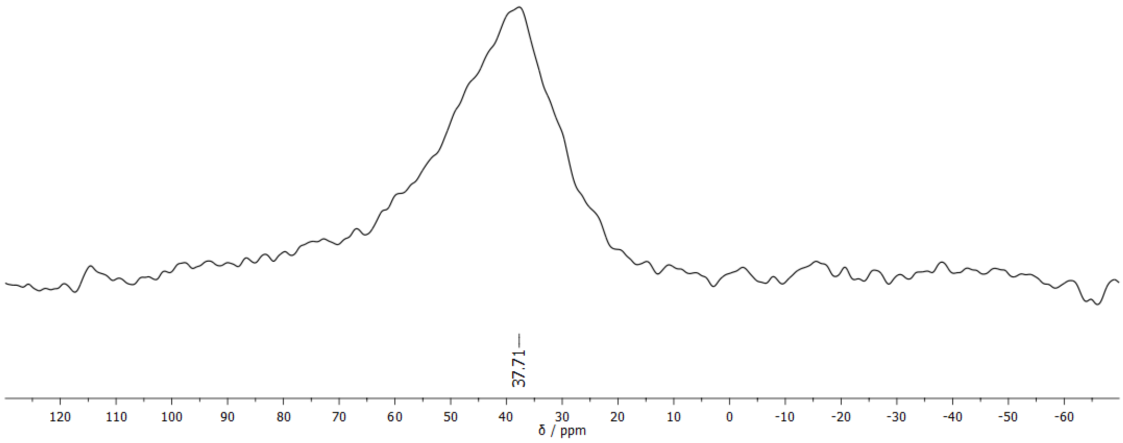

Figure S14. 224 MHz ^{11}B-NMR of **3** in C_6D_6.

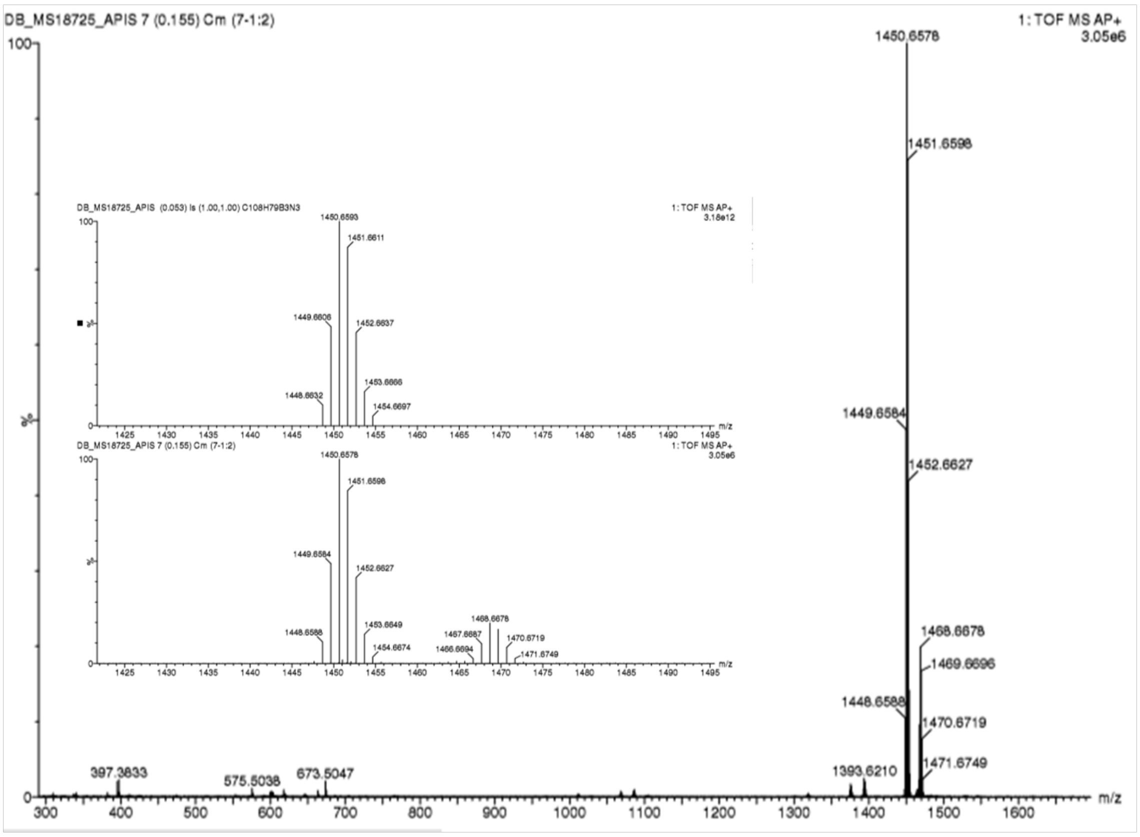

Figure S15. AP$^+$ HR-MS of **3**.

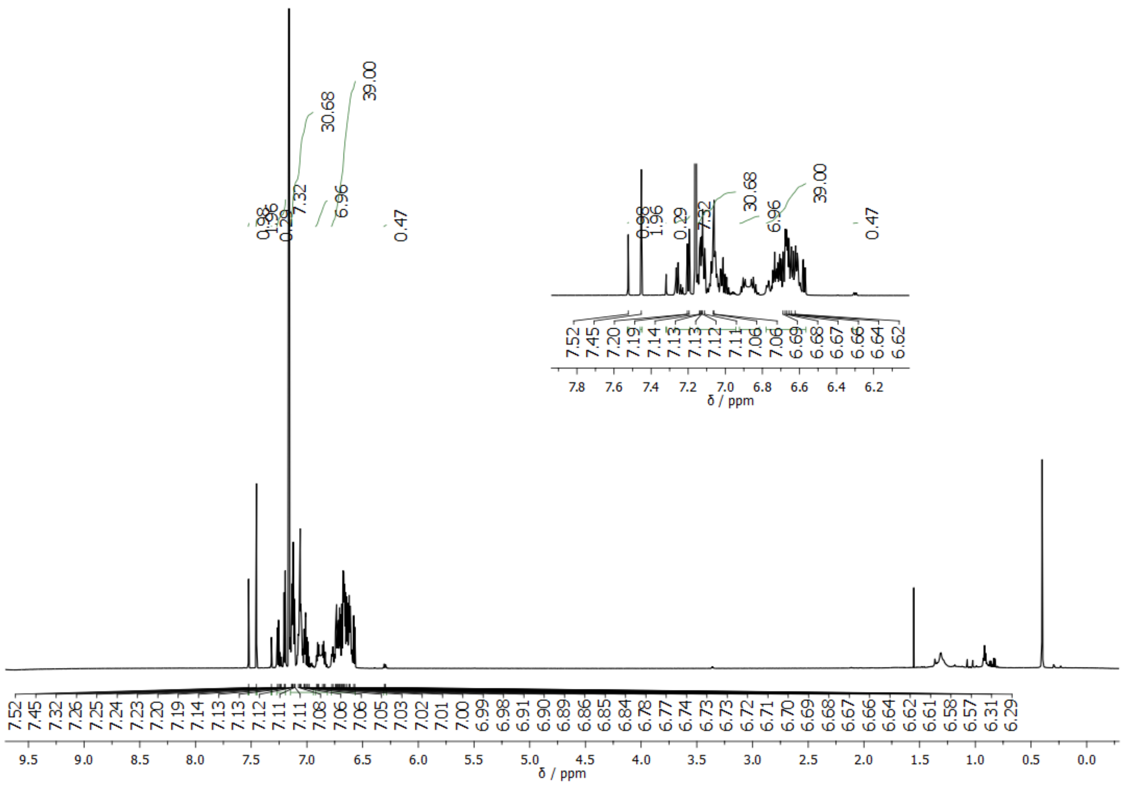

Figure S16. 700 MHz ^{1}H-NMR of **3** in C_6D_6.

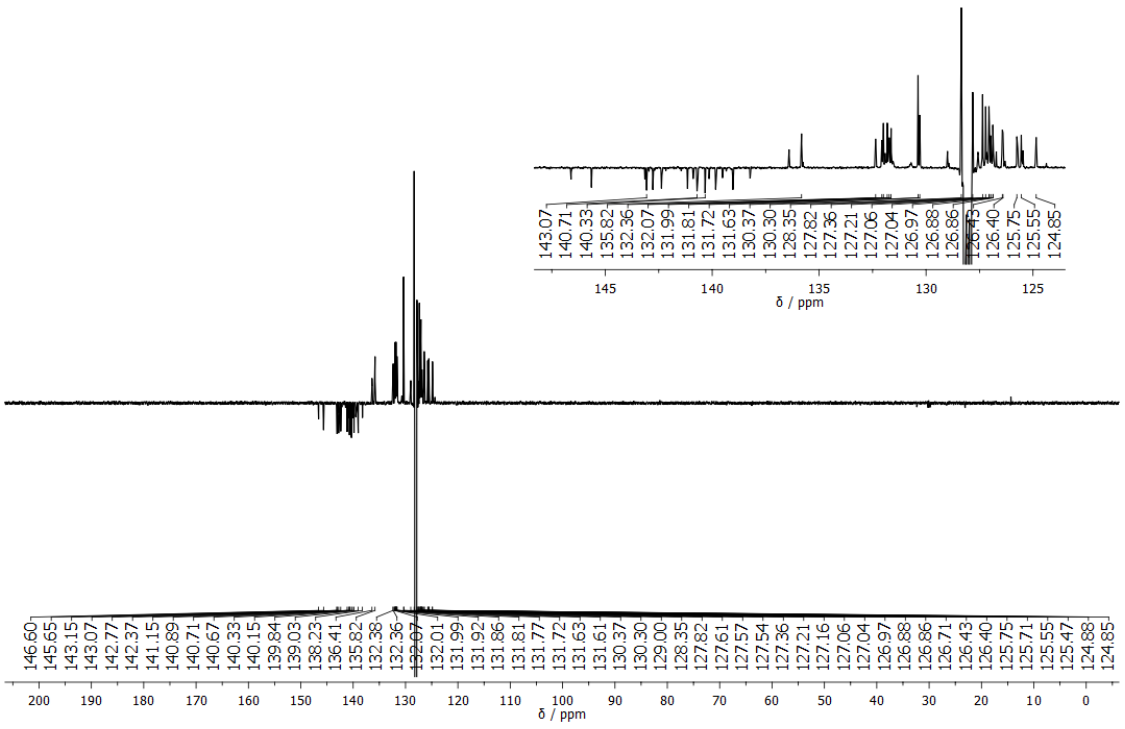

Figure S17. 176 MHz ^{13}C DEPTq-NMR of **3** in C_6D_6.

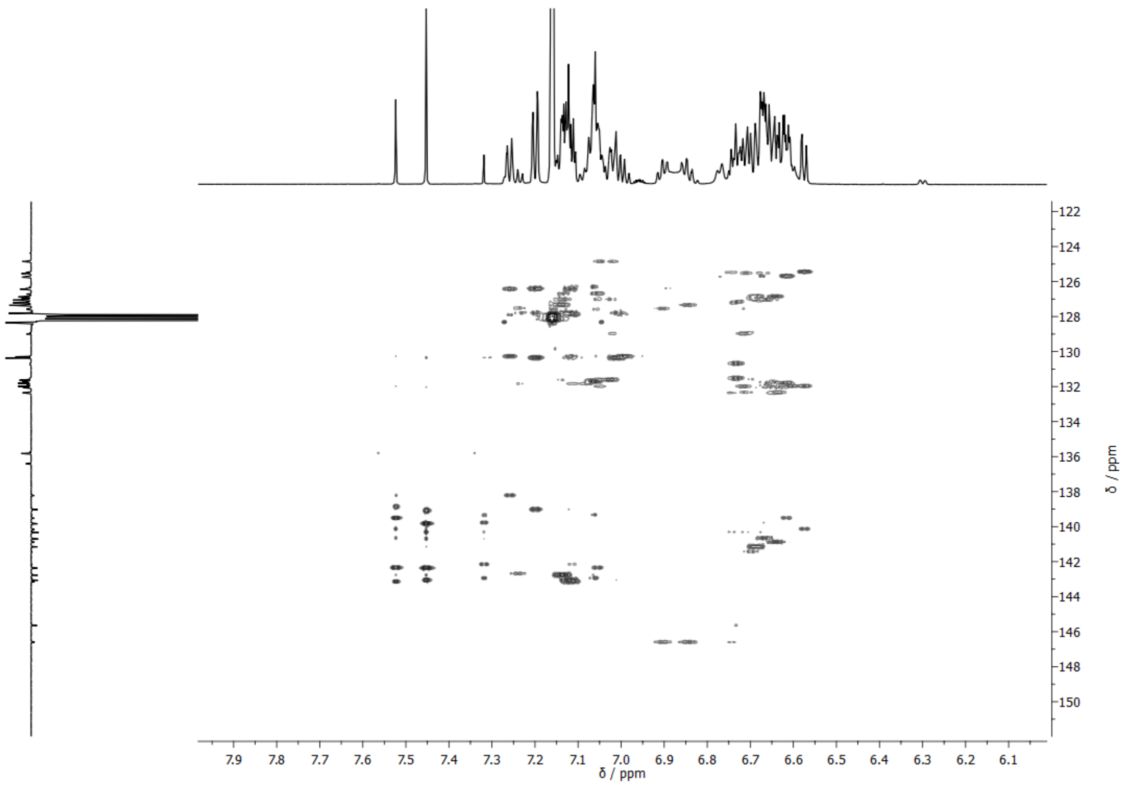

Figure S18. ^{1}H-^{13}C HMBC-NMR of **3** in C_6D_6.

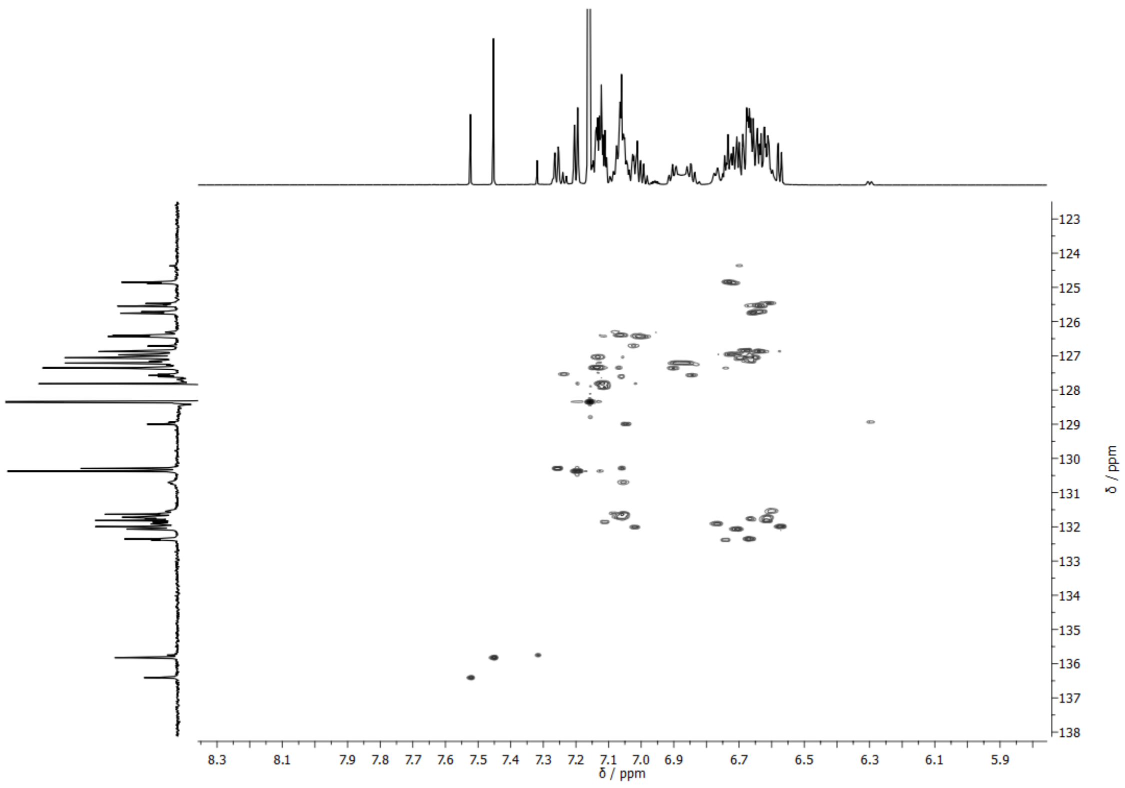

Figure S19. ^{1}H-^{13}C HSQC-NMR of **3** in C_6D_6.

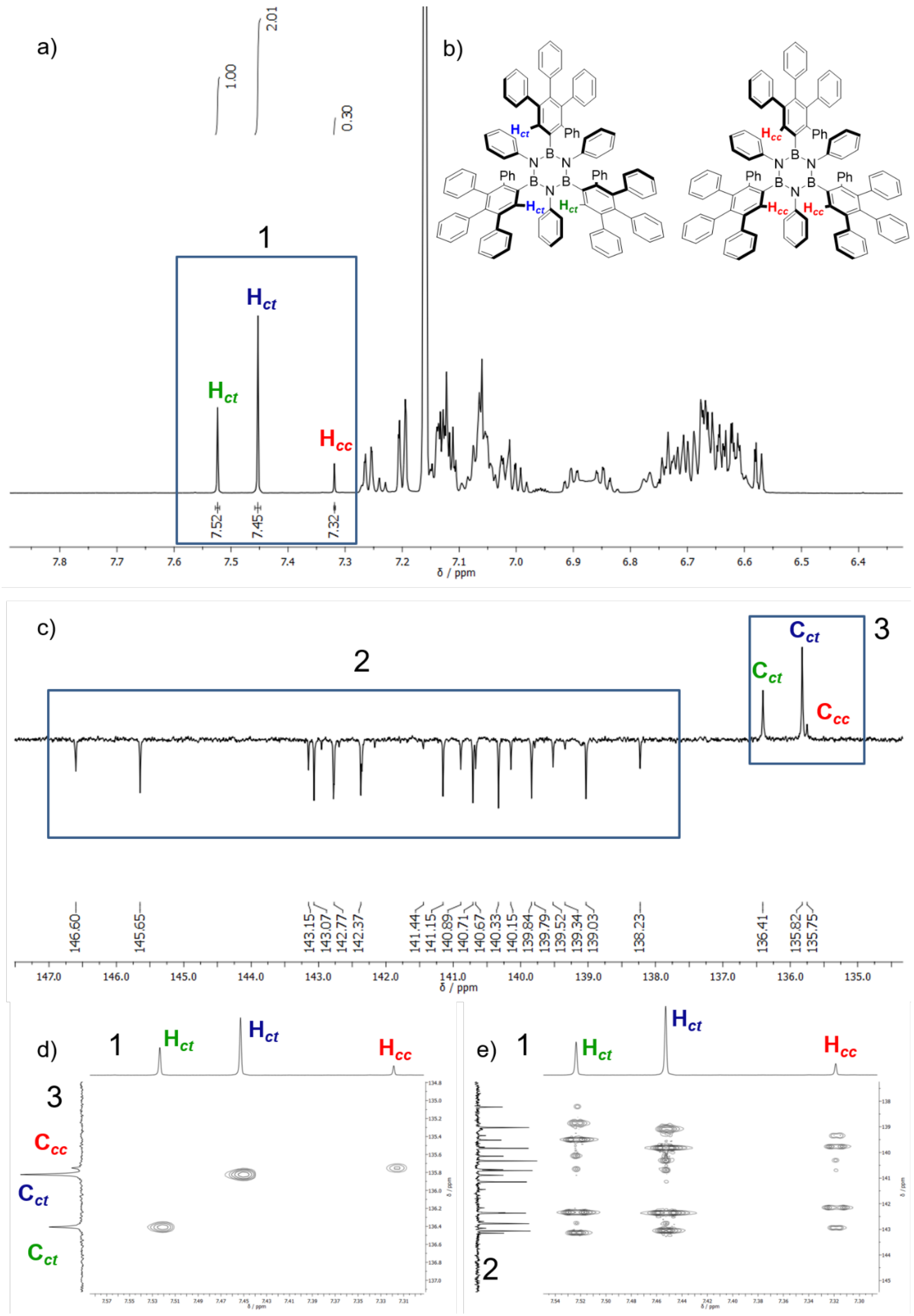

Figure S20. Complete interpretation of spectra from S15 to S18 with significant zooms. a) Zoom of the aromatic signal of 700 MHz ^{1}H-NMR of **3**; b) Chemical structure of atropoisomers *ct* and *cc* of **3** with diagnostic protons highlighted; c) 176 MHz ^{13}C DEPTq-NMR of **3**; d) Detail of ^{1}H-^{13}C HSQC-NMR for protons H_{ct} and H_{cc}; e) Detail of ^{1}H-^{13}C HMBC-NMR for protons H_{ct} and H_{cc}; All spectra were performed in C_6D_6.

4.3 **Characterization of 1,4-Bis(phenylethynyl)benzene (4)**

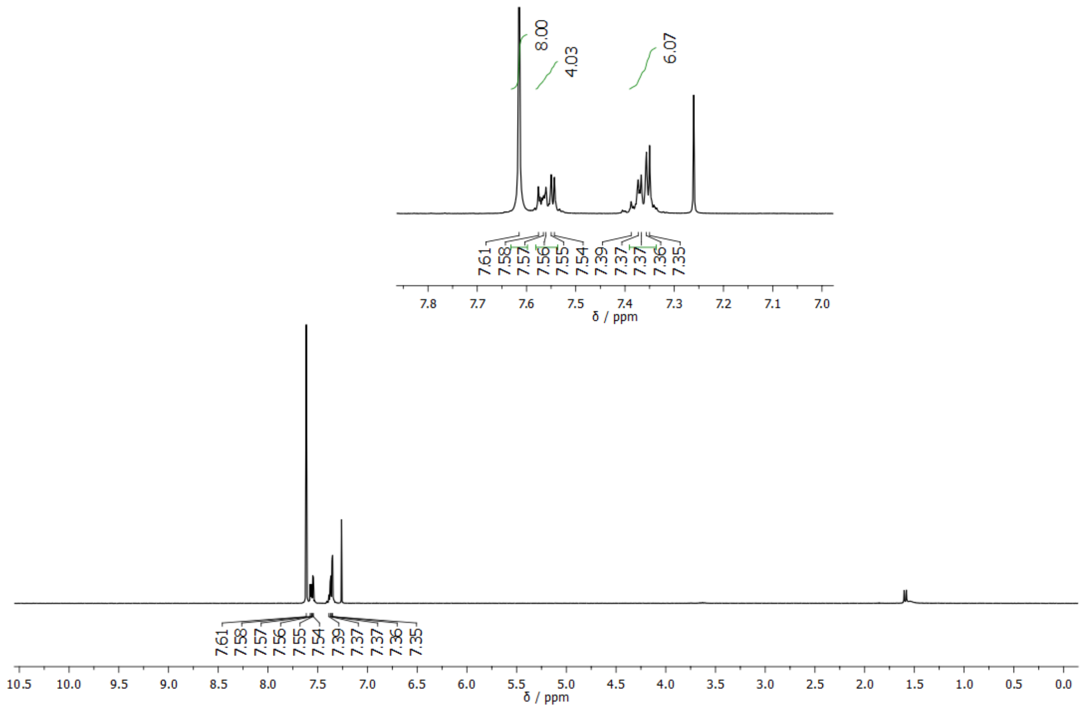

Figure S21. 300 MHz ^{1}H-NMR of **4** in $CDCl_3$.

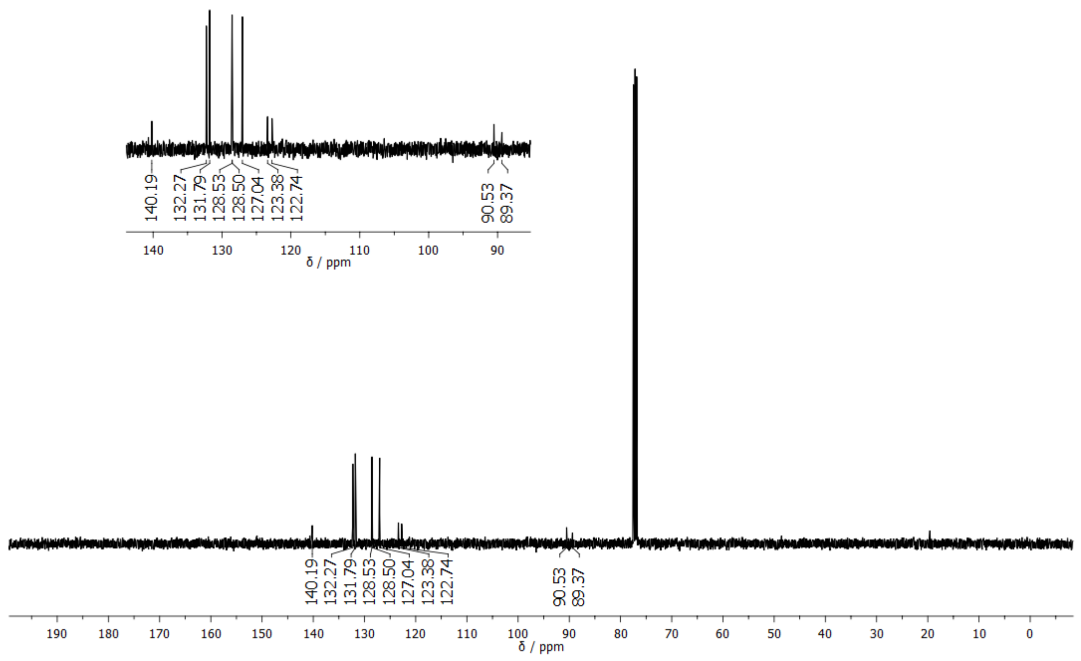

Figure S22. 100 MHz ^{1}H-NMR of **4** in $CDCl_3$.

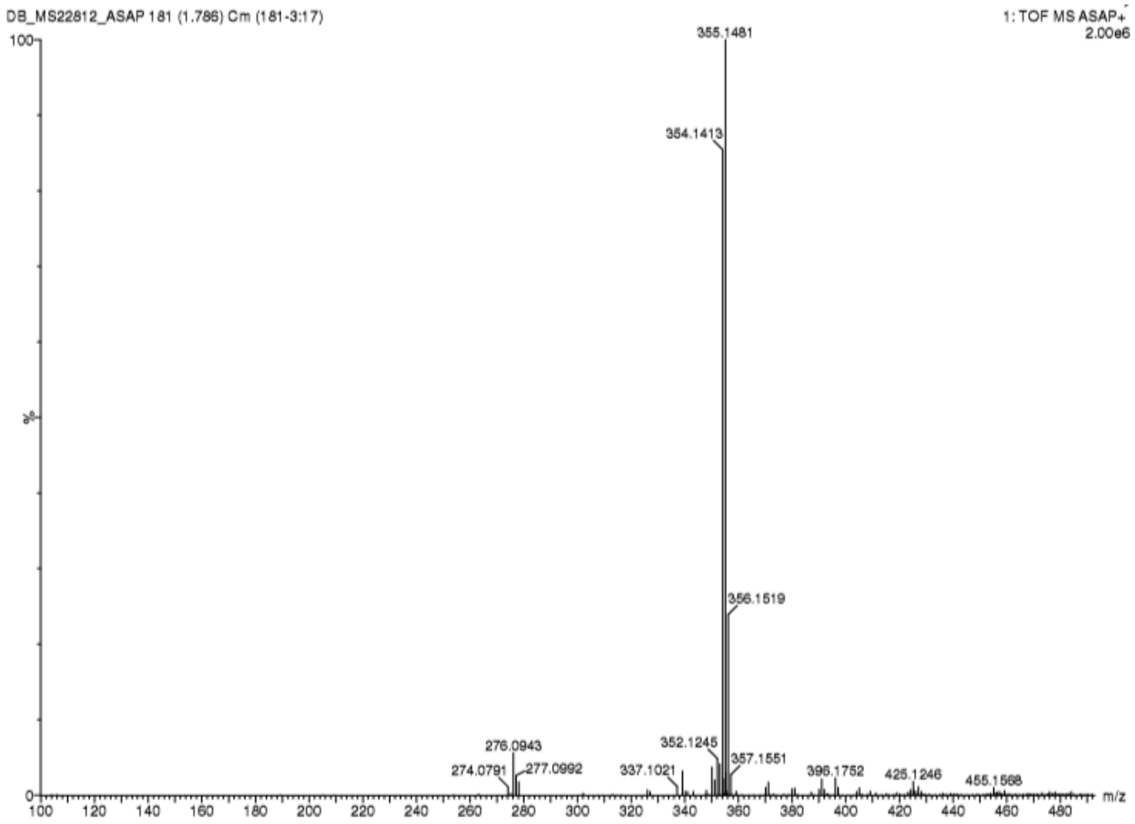

Figure S23. ASAP$^+$ HR-MS of **4**.

4.4 **Characterization of 2,2'-(1,4-Phenylene)bis(1-phenylethane-1,2-dione) (5)**

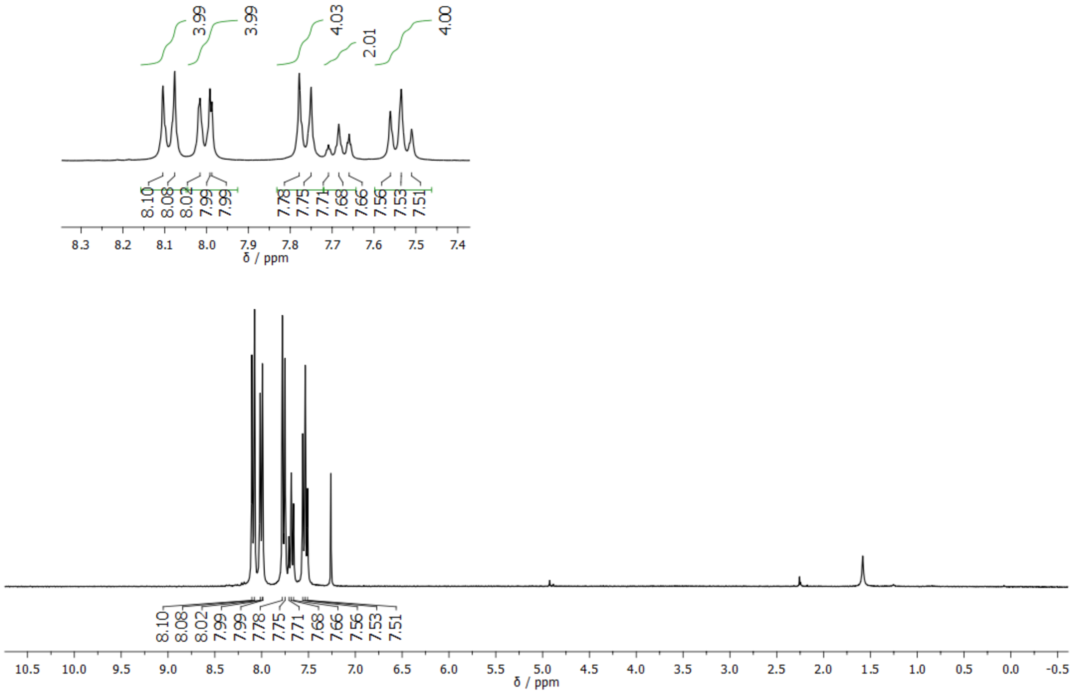

Figure S24. 300 MHz ^{1}H-NMR of **5** in $CDCl_3$.

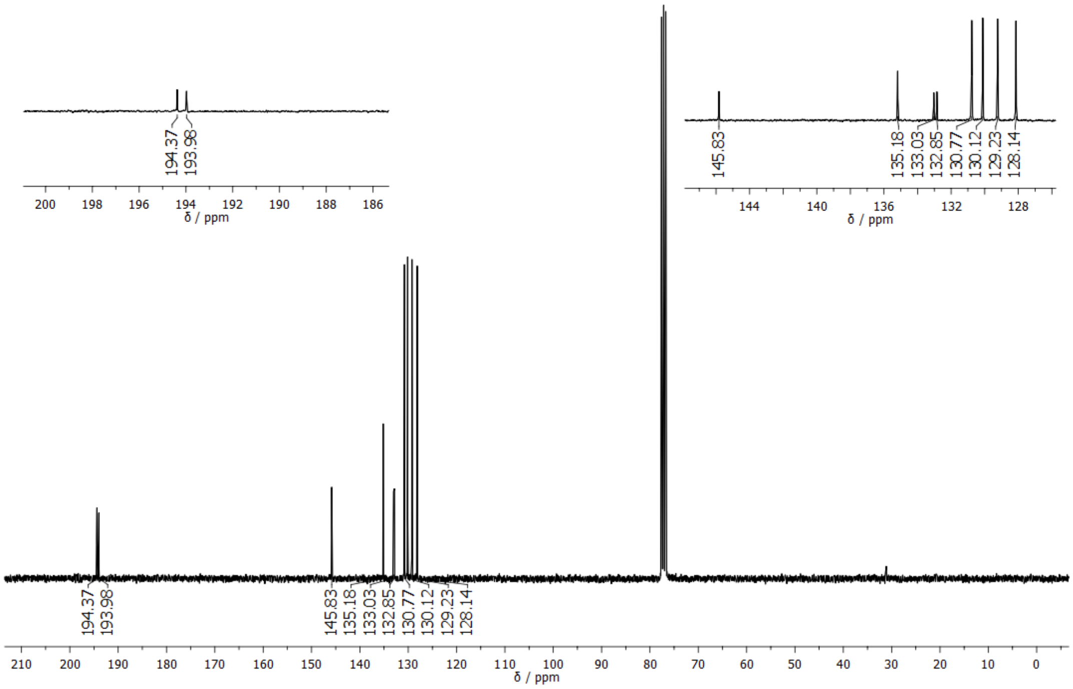

Figure S25. 75 MHz ^{1}H-NMR of **5** in $CDCl_3$.

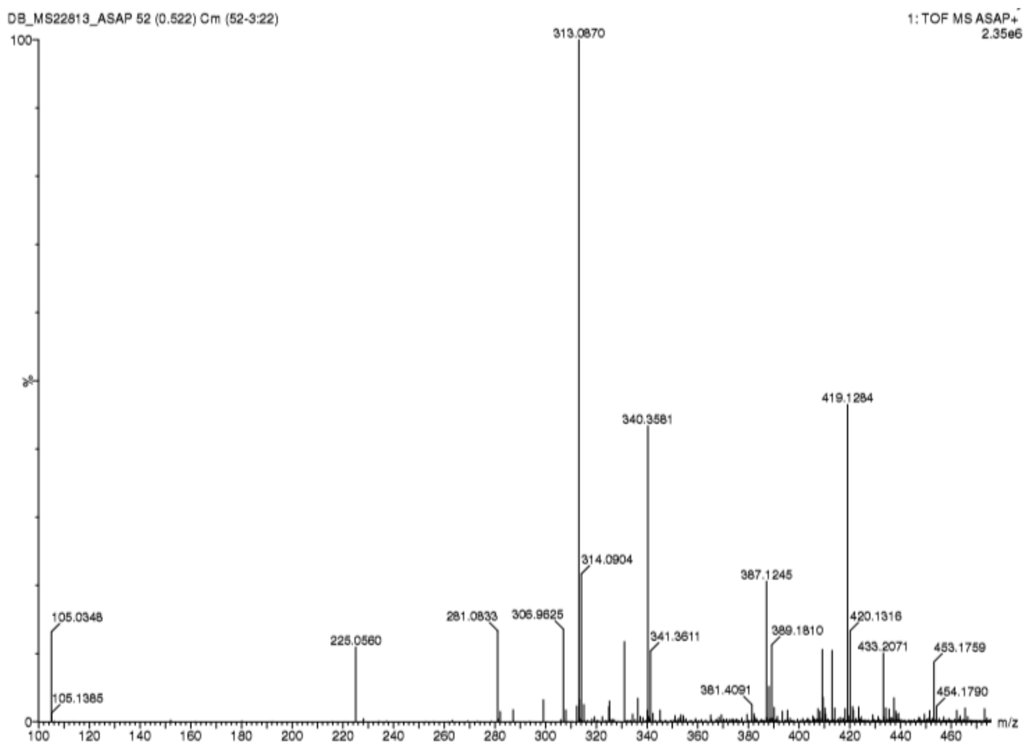

Figure S26. ASAP$^+$ HR-MS of **5**.

4.5 **Characterization of 4,4'-([1,1'-Biphenyl]-4,4'-diyl)bis(2,3,5-triphenylcyclopenta-2,4-dien-1-one) (6)**

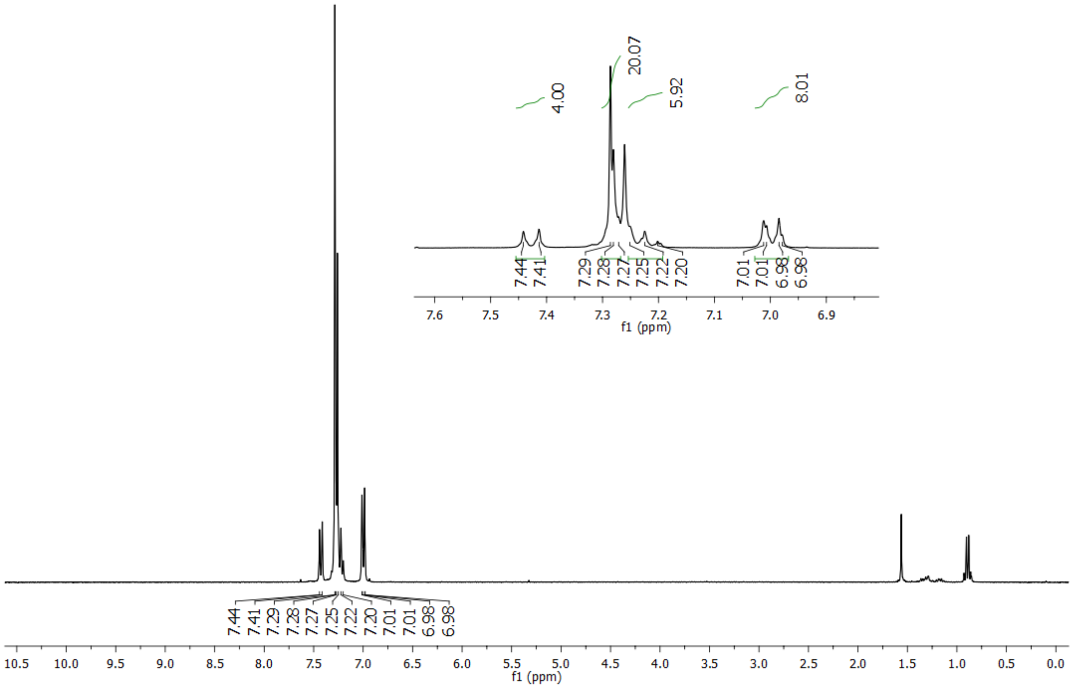

Figure S27. 300 MHz ^{1}H-NMR of **6** in $CDCl_3$.

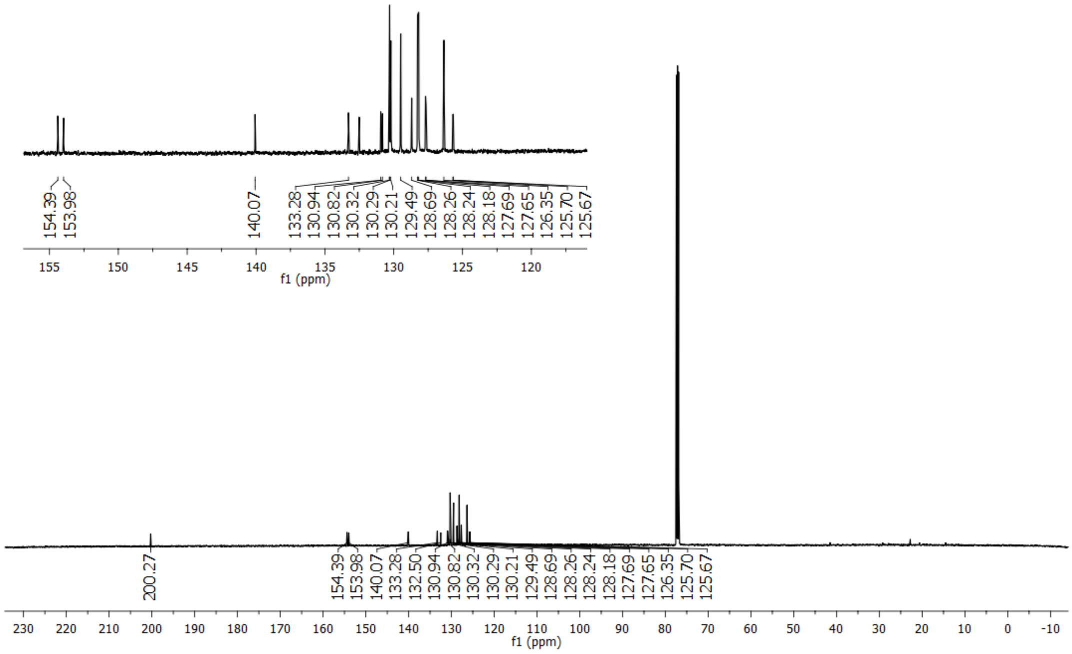

Figure S28. 126 MHz ^{13}C-NMR of **6** in $CDCl_3$.

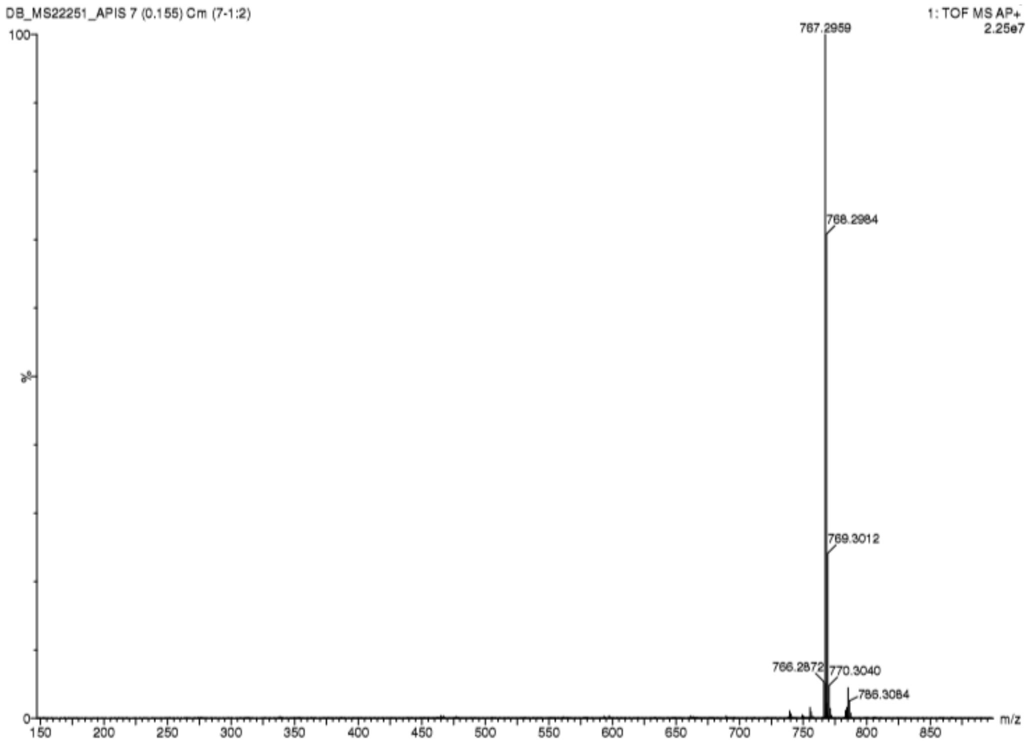

Figure S29. AP^+ HR-MS of **6**.

4.6 **Characterization of BN-Polymer**

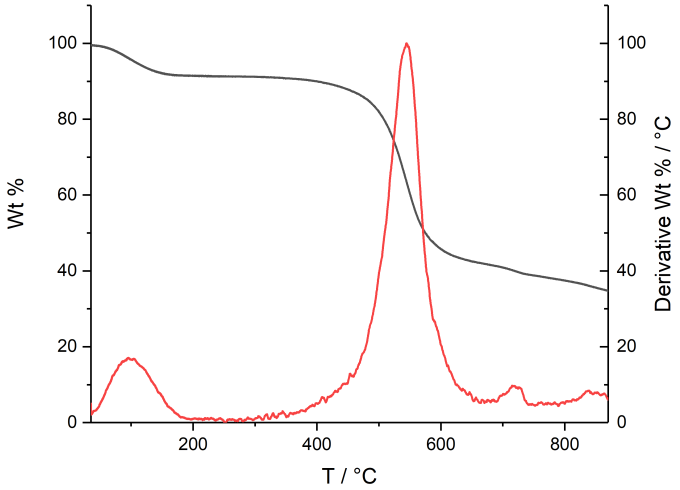

Figure S30. TGA trace of **BN-Polymer** performed under N_2.

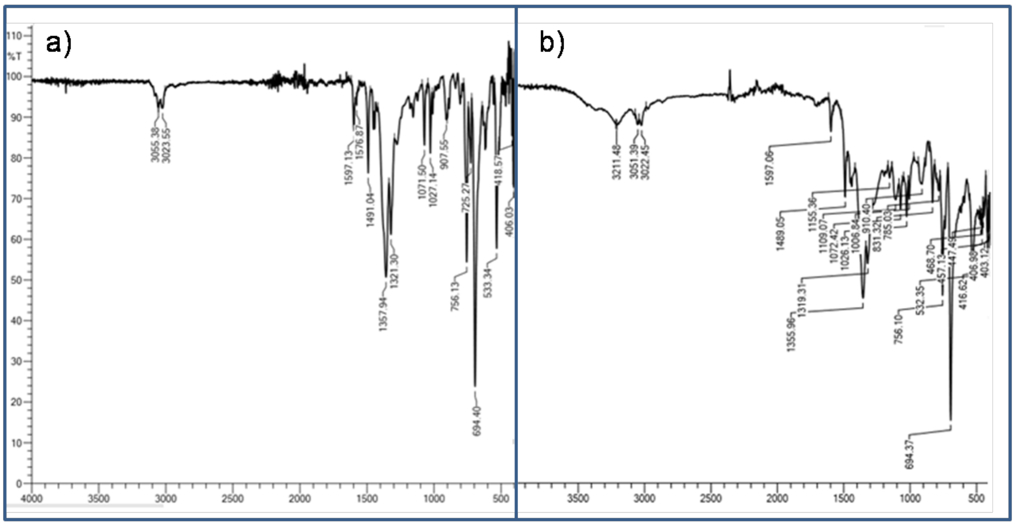

Figure S31. a) ATR-IR of **3** (mixture of isomers). b) ATR-IR of **BN-Polymer**.

4.7 Characterization of *B,B',B''*-tripropynyl-*N,N',N''*-triphenyl-borazine (7)

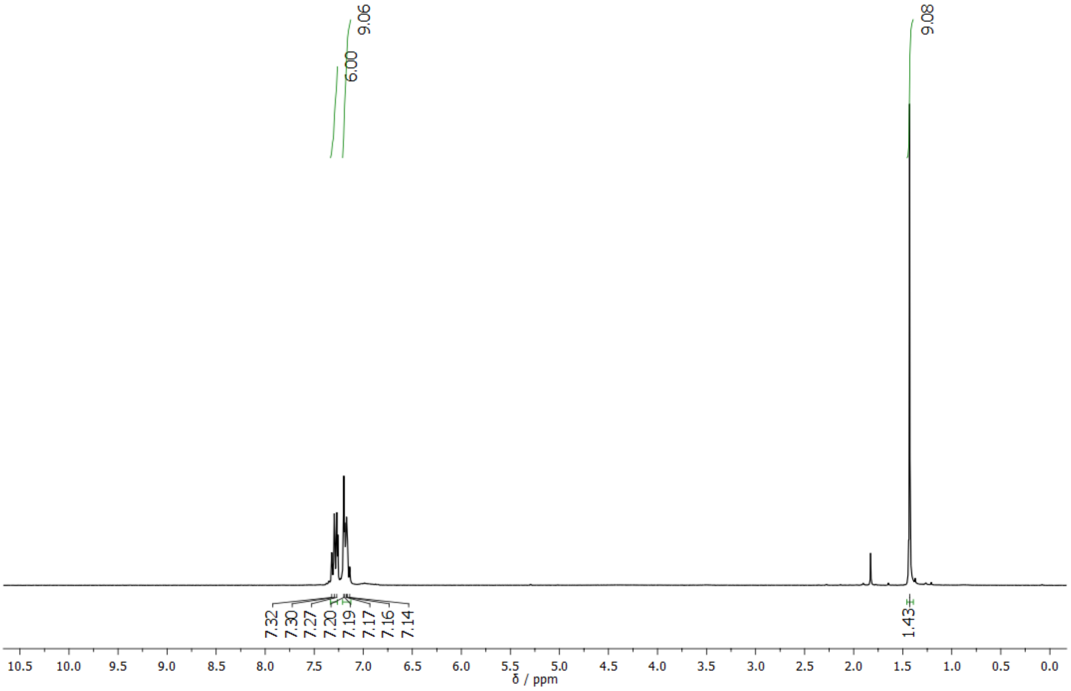

Figure S32. 300 MHz ^{1}H-NMR of **7** in $CDCl_3$.

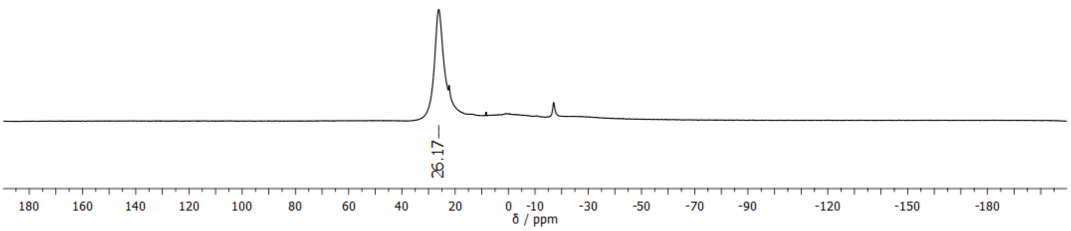

Figure S33. 160 MHz ^{11}B-NMR of **7** in $CDCl_3$.

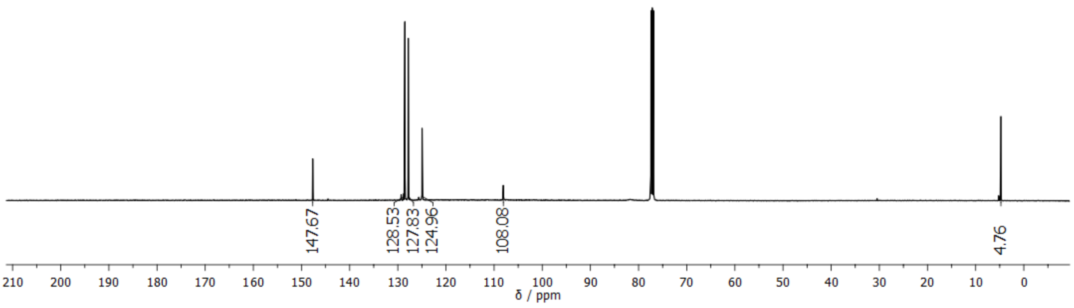

Figure S34. 126 MHz ^{13}C-NMR of **7** in $CDCl_3$.

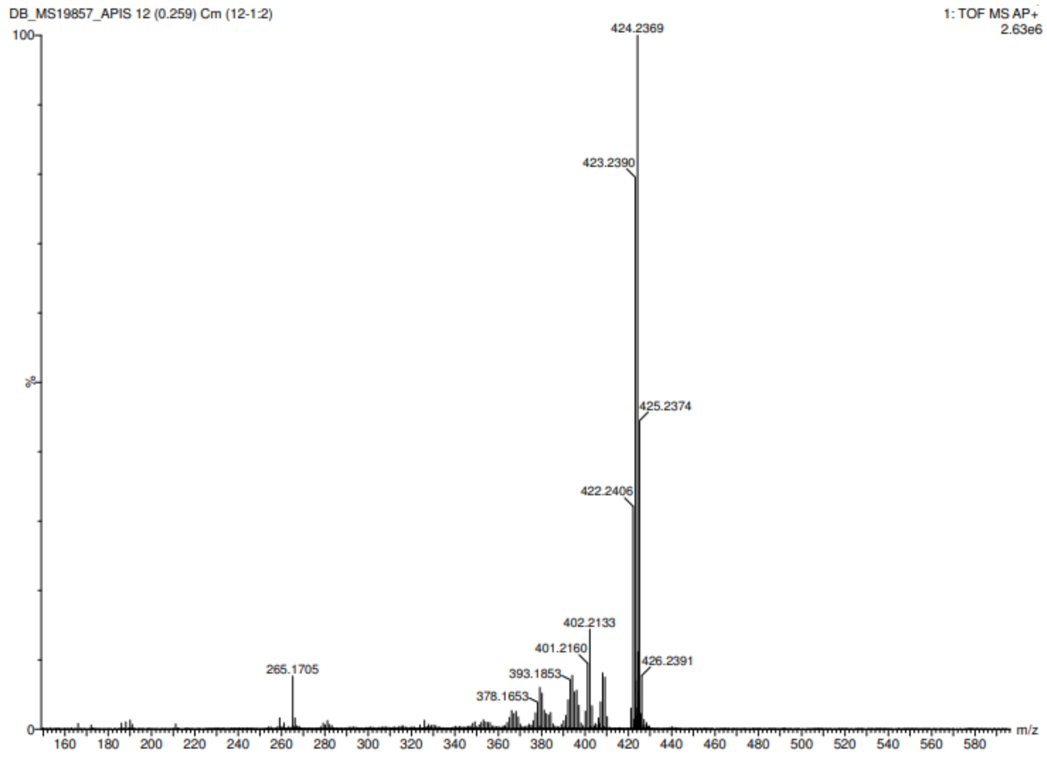

Figure S35. AP$^+$ HR-MS of **7**.

5 Characterization of BN-Polymer

5.1 Solid-State $^1H \rightarrow ^{13}C$ CPMAS NMR and Solid-State ^{11}B-MQMAS NMR

Experimental: Solid-state $^1H \rightarrow ^{13}C$ CPMAS NMR spectra were recorded on a Bruker Avance III HD spectrometer at 9.4 T [Larmor frequencies: 400.2 MHz (^1H), 100.6 MHz (^{13}C)] using ramped CP[14] for the borazine precursor **2**, the reference **3** and the **BN-Polymer** material, with magic angle spinning frequencies of 10 kHz, 12 kHz and 12 kHz, respectively. ^{11}B MQMAS NMR spectra were recorded on a Bruker Avance III HD spectrometer at 9.4 T [Larmor frequency: 128.4 MHz (^{11}B)] using a four-pulse split-t_1 sequence[15] with the indirect dimension scaled to have the same contribution from the isotropic shift as the direct dimension.[16]

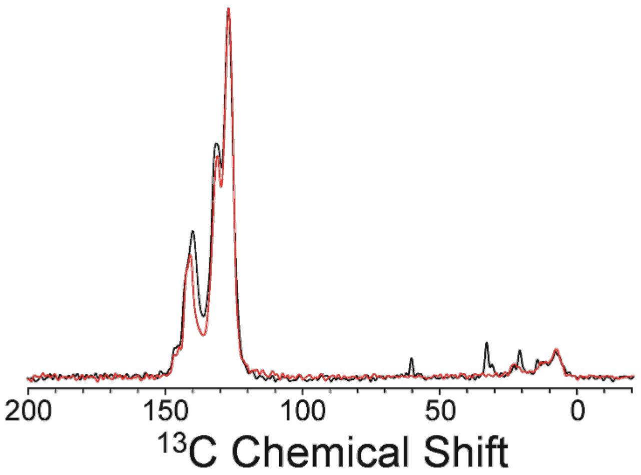

Figure S36. Overlay of the solid-state $^1H \rightarrow ^{13}C$ CPMAS NMR spectra recorded for cycloadduct **3** (red) and the **BN-Polymer** (black).

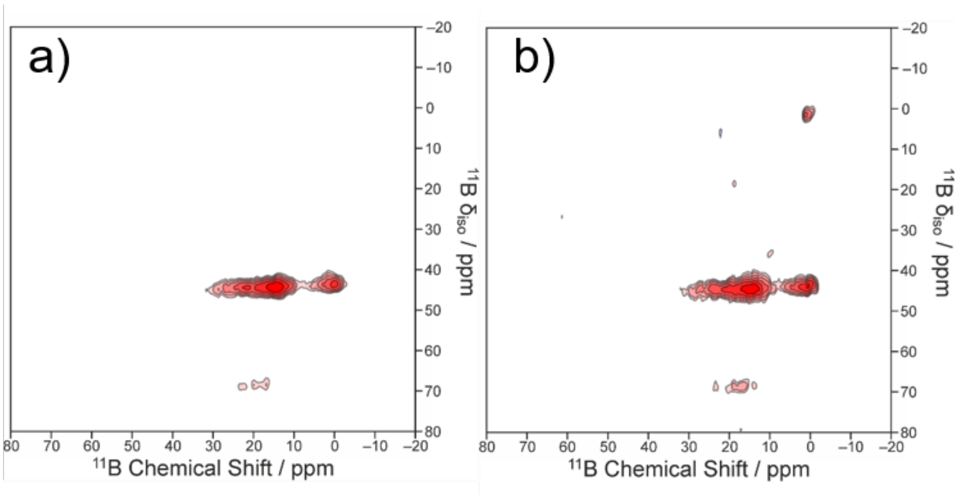

Figure S37. Solid-state ^{11}B MQMAS NMR spectra, shown as intensity contour plots, for a) cycloadduct **3** and b) the **BN-Polymer**. The contours are on a logarithmic scale using a factor of 1.3 between adjacent contours.

Solid-state ^{11}B MQMAS NMR spectra recorded for samples of **3**, and the **BN-polymer** are shown in Figure S37. In each case, the solid-state ^{11}B MQMAS NMR spectrum contains two broad peaks with shifts in the indirect dimension between 40 and 50 ppm (the peak at 0 ppm in both dimensions in the spectrum of the polymer is a diagonal artefact, which may be ignored). One-dimensional projections of these spectra onto the direct dimension (horizontal in Figure S37) are shown in Figure 3d (main paper). These one-dimensional projections were obtained from the ^{11}B MQMAS NMR spectra (Figure S37) by summation of all data between 39 and 49 ppm along the indirect dimension (vertical in Figure S37).

The shapes of the peaks are very similar for the samples of **3** and the **BN-polymer**, as clearly evident both from the two-dimensional contour plots in Figure S37 and from the one-dimensional projections onto the direct dimension shown in Figure 3d, indicating that both **3** and the **BN-polymer** have similar values of the quadrupolar parameters and isotropic chemical shifts for the ^{11}B environments. This observation suggests that the local structure in the vicinity of the central B_3N_3 rings (including the conformations of the substituents bonded to the B atoms) is very similar in both **3** and the **BN-polymer**.

5.2 BN gel studies

Low Field Nuclear Magnetic Resonance (LF NMR) represents a non-destructive, non-invasive and low-cost approach requiring only minimal or no sample preparation.[17] LF NMR detects the response of hydrogen protons (H^+) in magnetic fields. H^+ has a property known as spin that causes the protons to act as small bar magnets. In the presence of an external magnetic field (B_0), H^+ will therefore tend to line up with B_0 field direction; the application of a second magnetic field B_1, orthogonal to B_0, orients all the dipole moments in the B_1 direction (transverse plane). After B_1 removal, the excited protons in B_1 plane give off energy and return to their equilibrium direction. Thus, the free induction decay (FID) intensity in the transverse plane, proportional to the module of the magnetization vector in the B_1 plane, decays according to a characteristic relaxation time (T_2).[18] Interestingly, T_2 not only depends on B_0 intensity and temperature, but it also depends on the presence of solid surfaces such as those competing to polymeric chains of a polymeric solution or gel. Indeed, water hydrogens near the solid surface relax faster than those in the bulk as surface behaves as magnetization absorber.[19] Hence, the higher the concentration of "solid" part, the faster the relaxation process is. This, in turn, reflects in a smaller T_2. In the case of not homogeneous systems such as gels, where meshes of different size can exist, the average T_2 depends on the relaxation time pertaining to each mesh size as, the smaller the mesh size, the faster the hydrogens relaxation is. Accordingly, FID decay ($I(t)$) can be described by a sum of exponential terms where A_i represents the volume fraction of water molecules relaxing with the relaxation time T_{2i}:[17]

$$I(t)=\sum_{i=1}^{m} A_i \exp(-t/T_{2i}) \qquad T_{2m}=\sum_{i=1}^{m} A_i T_{2i} / \sum_{i=1}^{m} A_i \qquad (1)$$

Where t is time and T_{2m} is the average relaxation time of protons. For its more immediate meaning, the % value of A_i is generally ($A_{i\%}$) in place of A_i value:

$$A_{i\%} = 100 * A_i / \sum_{i=1}^{m} A_i \quad (2)$$

The number, m, of the relaxation times (T_{2i}) constituting the relaxation time distribution (A_i, T_{2i}) was determined by minimizing the product $\chi^2 * (2m)$, where χ^2 is the sum of the squared errors and $2m$ represents the number of fitting parameters of eq.(1).[20]

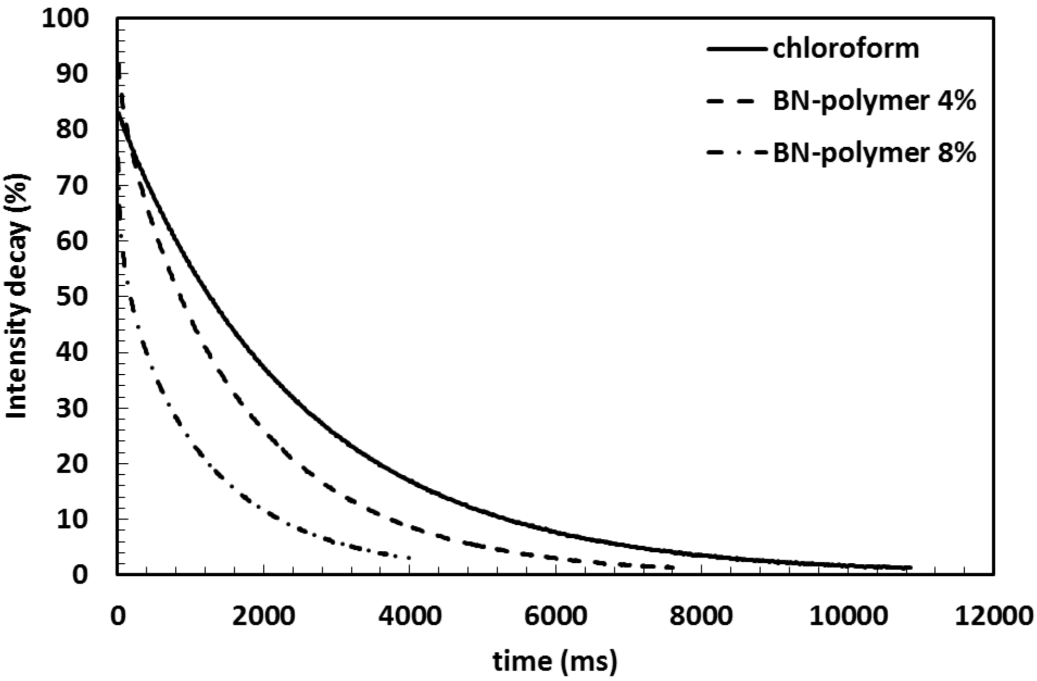

Figure S38. FID intensity decay relative to two different polymer concentrations (solvent $CHCl_3$). Solid line represents the relaxation process of pure $CHCl_3$ (10 °C). **BN-Polymer** 2% data is not shown.

Table S2 shows the average relaxation times (T_{2m}) relative to the eq (1) best fitting to the data (Figure S38).

	T_{2m} (ms)
$CHCl_3$	2531
**BN-Polymer** 2%	2181
**BN-Polymer** 4%	1591
**BN-Polymer** 8%	935

Table S2 Average relaxation time T_{2m} for the investigated samples.

Rheological measurements. To evaluate the extension of the linear viscoelastic range, oscillatory stress sweep tests were performed in the stress (τ) range spanning form 0.33 Pa to 12 Pa at 1 Hz. Then, frequency sweep tests, performed inside the linear viscoelastic region (τ = 1 Pa), allowed the determination of the systems mechanical spectra (dependence of the elastic (*G'*) and viscous (*G''*) moduli on pulsation ω= 2πf) in the frequency range spanning from 0.1 to 10 Hz. The generalized Maxwell model[21] was used to fit the experimental mechanical spectra in order to determine the shear modulus *G* given by the sum of the elastic spring constants (g_i) of all the Maxwell elements:[22]

$$G' = \sum_{i=1}^{n} g_i \frac{(\lambda_{i\omega})^2}{1+(\lambda_{i\omega})^2} \quad (3)$$

$$G'' = \sum_{i=1}^{n} g_i \frac{\lambda_{i\omega}}{1+(\lambda_{i\omega})^2} \quad (4)$$

$$G = \sum_{i=1}^{n} g_i \quad (5)$$

where λ_i is the relaxation time of the ith Maxwell element. The number, *n*, of the Maxwell elements was determined by minimizing the product χ^2*(*n*+1), where χ^2 is the sum of the squared errors and (*n*+1) represents the number of fitting parameters of eq.(3)-(4).[20] Indeed, following a consolidate strategy[21], eq.(3) and (4) fitting to experimental mechanical spectra was performed assuming that relaxation times (λ_i) were scaled by a factor 10 (λ_{i+1}=10λ_i).

The evaluation of the average network mesh size (ξ_{RHEO}) was performed starting from the Flory theory:[23]

$$\rho_x = G/RT \quad (6)$$

where ρ_x is the network crosslink density, defined as the moles of crosslinks between different polymeric chains per gel unit volume, *T* is temperature and *R* is the universal gas constant. The link between ρ_x and ξ_{RHEO} is provided by the equivalent network theory,[24] according to which the following relation holds:

$$\xi = \sqrt[3]{6/\pi\rho_x N_A} \quad (7)$$

where N_A is Avogadro number.

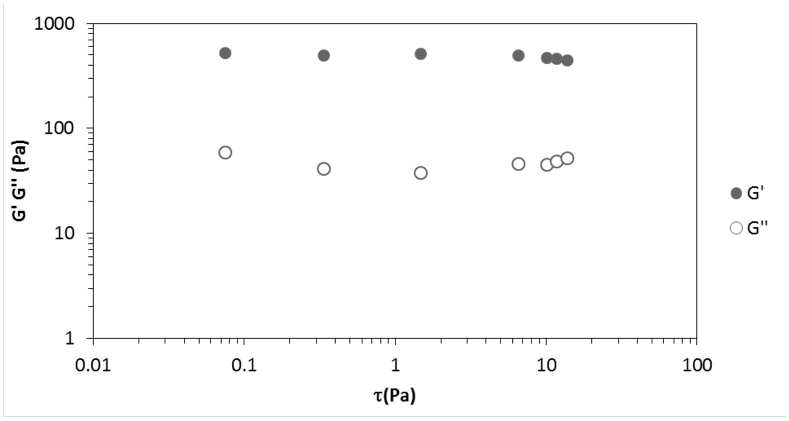

Figure S39. Short stress sweep (SSS) at 1 Hz.

6 Solid-state electrolyte characterization

6.1 Characterization

The thermal stability of the obtained **BN-polymer**/TEGDME/$LiClO_4$ based solid state electrolytes was evaluated by thermogravimetric analysis (TGA) using a TGA/SDTA-851 instrument from METTLER TOLEDO at a heating rate of 10 °C.min^{-1} from room temperature to 1000 °C under N_2 flow. Differential scanning calorimetry (DSC) experiments were performed with a METTLER TOLEDO DSC-1 STAR^e System instrument under a N_2 flow at a ramp rate of 10 °C.min^{-1} in the temperature range of 30-250 °C.

Powder X-ray diffraction (XRD) data were recorded on a STOE StadiP X-ray diffractometer with CuKα_1 radiation (λ = 1.5418 Å) from 2θ = 1.5° up to 60° with 0.02° increment, using an operation voltage and current of 40 kV and 40 mA, respectively.

Scanning electron microscopy (SEM) images were obtained with a FEI XL30 FEG equipped with a Raith laser interferometer-controlled stage operated at 10 kV and the Elphy Plus software.

The ionic conductivity of the optimized films of **BN-polymer**/TEGDME/$LiClO_4$ based SSEs was studied by electrochemical impedance spectroscopy (EIS) using a Biologic Science Instruments VMP-300 potentiostat analyser. The SSEs were sandwiched between two stainless steel electrodes in a symmetric stainless steel (SS)/**BN-Polymer**/TEGDME/$LiClO_4$/SS configuration. The samples were thermally equilibrated at room temperature for 1 h prior to the measurements in the frequency range from 1.0 mHz to 1.0 MHz with the amplitude of 10 mV.

The bulk resistance of the samples was calculated from the Nyquist curves and the ionic conductivity was calculated from the relation: $\sigma = t/(R_b A)$, where σ is the ionic conductivity, R_b the bulk resistance, t and A are the thickness and the area of the SSE samples, respectively.

where σ is the ionic conductivity (S·cm^{-1}), R_b the bulk resistance, t and A are the thickness and the area of the SSE samples, respectively.

The electrochemical stability window of the SSE was tested at r.t. using linear sweep voltammetry (LSV) and cyclic voltammetry (CV). The tests were carried out using Swagelok cells assembled in argon filled glove box (H_2O and O_2< 0.1 ppm) in a stainless steel (SS)/**BN-Polymer**/TEGDME/$LiClO_4$/Li configuration using stainless steel working electrode and Li metal as reference and counter electrode. The potential of the cell was scanned at r.t. from 0.0 to 5.0 V *vs* Li/Li$^+$ at constant sweep rate 1.0 mV.s^{-1} for LSV and at a scan rate of 1.0 m.Vs^{-1} in the range of 0.1-5.0 V for CV measurements. The Li-ion transference number (t_{Li}^+) describes the relative amount of the transporting lithium ion compared to that of the counter anion.[25,26] The t_{Li}^+ measurement was achieved at r.t. by combining AC impedance and DC polarization of the assembled

Li/**BN-Polymer**/TEGDME/$LiClO_4$/Li cell configuration and polarized under a fixed direct current voltage of 10 mV. The $t_{Li}{}^{+}$ was calculated from the Bruce-Vincent-Evans equation[26–28]:

$$t_{Li^+} = \frac{I_S(\Delta V - I_0 R_0)}{I_0(\Delta V - IsRs)} \quad (8)$$

where I_o and I_S are the initial and steady-state currents, R_0 and R_S are the initial and steady-state interfacial resistances and ΔV is the applied polarization voltage.

6.2 XRD analysis and SEM analysis

XRD patterns of $LiClO_4$, **BN-Polymer** and **BN-Polymer**/TEGDME/$LiClO_4$ at different wt.% $LiClO_4$contents (15, 20 and 25 wt.%) electrolytes are shown in Figure S40. Two broad peaks can be observed around 2θ = 3.5° and 7°, which indicate the amorphous character of the pure **BN-Polymer**. The addition of $LiClO_4$ to **BN-Polymer**/TEGDME shows a new crystalline peak around 2θ = 8° that may be due to $LiClO_4$ clusters forming in the **BN-Polymer**/TEGDME matrix or to the formation of crystalline EO-$LiClO_4$ complexes.[29]

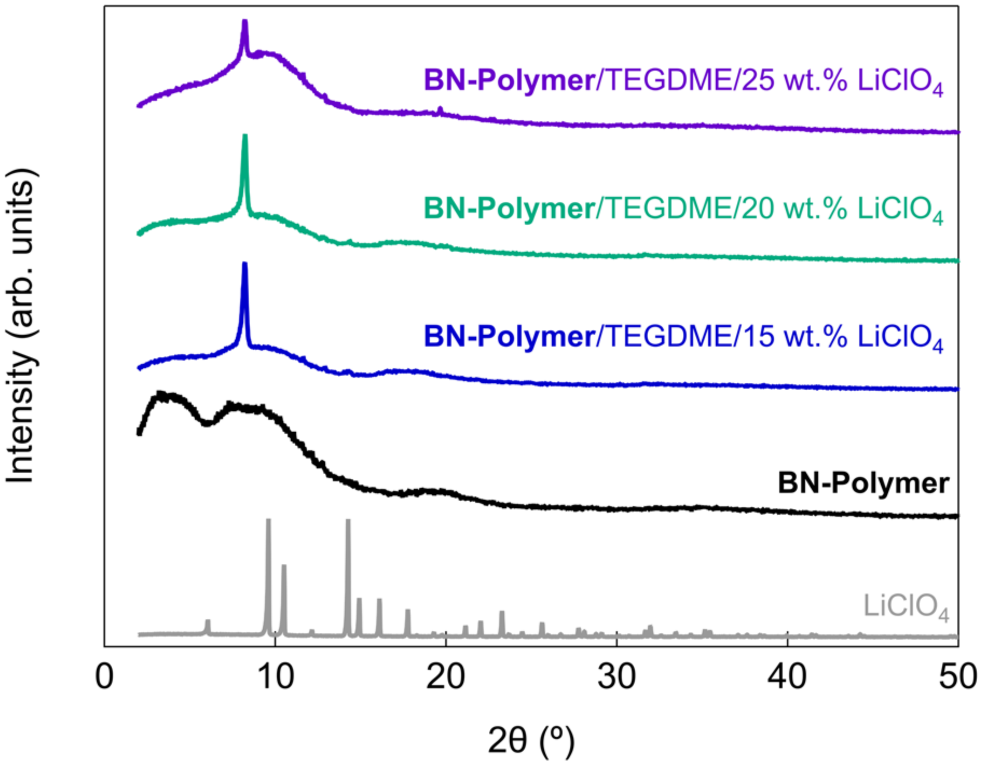

Figure S40. XRD pattern of $LiClO_4$, **BN-Polymer** and **BN-Polymer**/TEGDME/$LiClO_4$ electrolytes at different wt.% $LiClO_4$ contents (15, 20 and 25 wt.%).

Morphology at micrometre scale of the processed **BN-Polymer**/TEGDME/$LiClO_4$ materials is displayed in Figure S41.

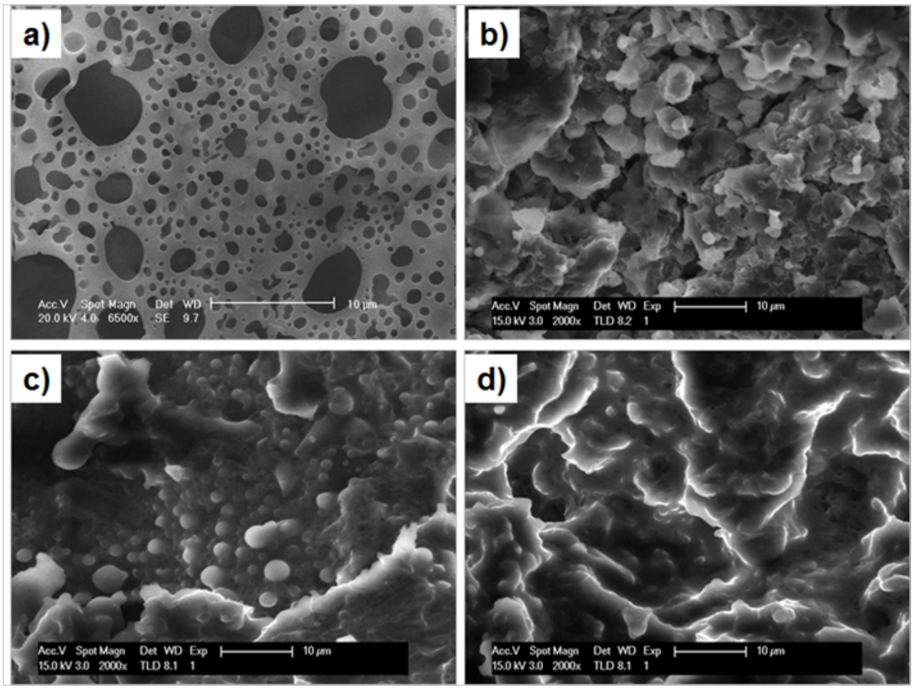

Figure S41. SEM images of a) **BN-Polymer** and **BN-Polymer**/TEGDME/$LiClO_4$ electrolytes at different wt.% $LiClO_4$ contents: b) 15 wt.%, c) 20 wt.%, and d) 25 wt.%.

6.3 Thermal analysis

To investigate the thermal stability of the obtained **BN-Polymer**/TEGDME/$LiClO_4$, thermogravimetric analysis (TGA) at a heating rate of 10 °C.min^{-1} from room temperature to 1000 °C under N_2 atmosphere (Figure S42a) and differential scanning calorimetry (DSC) experiments were performed under nitrogen flow at a ramp rate of 10 °C.min^{-1} (Figure S42b).

Despite its high boiling point of 275 °C, the mass loss of the neat TEGDME starts at around 150 °C due probably to the evaporation initiated by the N_2 flow.[30] $LiClO_4$ decomposes from 500 °C followed by an evaporation of the residues around 600 °C. The pure **BN-Polymer** decomposed in one step beginning at 450 °C with a residue of about 50% after 650 °C. As expected, **BN-Polymer**/TEGDME/$LiClO_4$ samples displayed the same behaviour. They exhibit a gradual weight loss of about 5% during the initial heating up to 100 °C, which can be considered as the result of moisture from the electrolytes at the time of loading the samples. Then the first significant weight loss about 45-55% is due to the TEGDME evaporation/or degradation at 150 °C, followed by a gradual loss of about 5% in the temperature range of 300-450 °C for $LiClO_4$. The complete decomposition of the sample takes place between 500 and 600 °C with the corresponding weight loss of about 75%.

The DSC thermograms obtained for **BN-Polymer**/TEGDME/$LiClO_4$ in the range of the temperature of 30-250 °C showed no endothermic peak associated with a melting temperature in any thermogram. This demonstrates that the polymer and the polymer-salt systems are amorphous. The absence of the temperature

of glass transition T_g in the investigated temperature range suggested the high rigidity of the **BN-Polymer** and the blocking chain segmental motion which could explain the low conductivity.

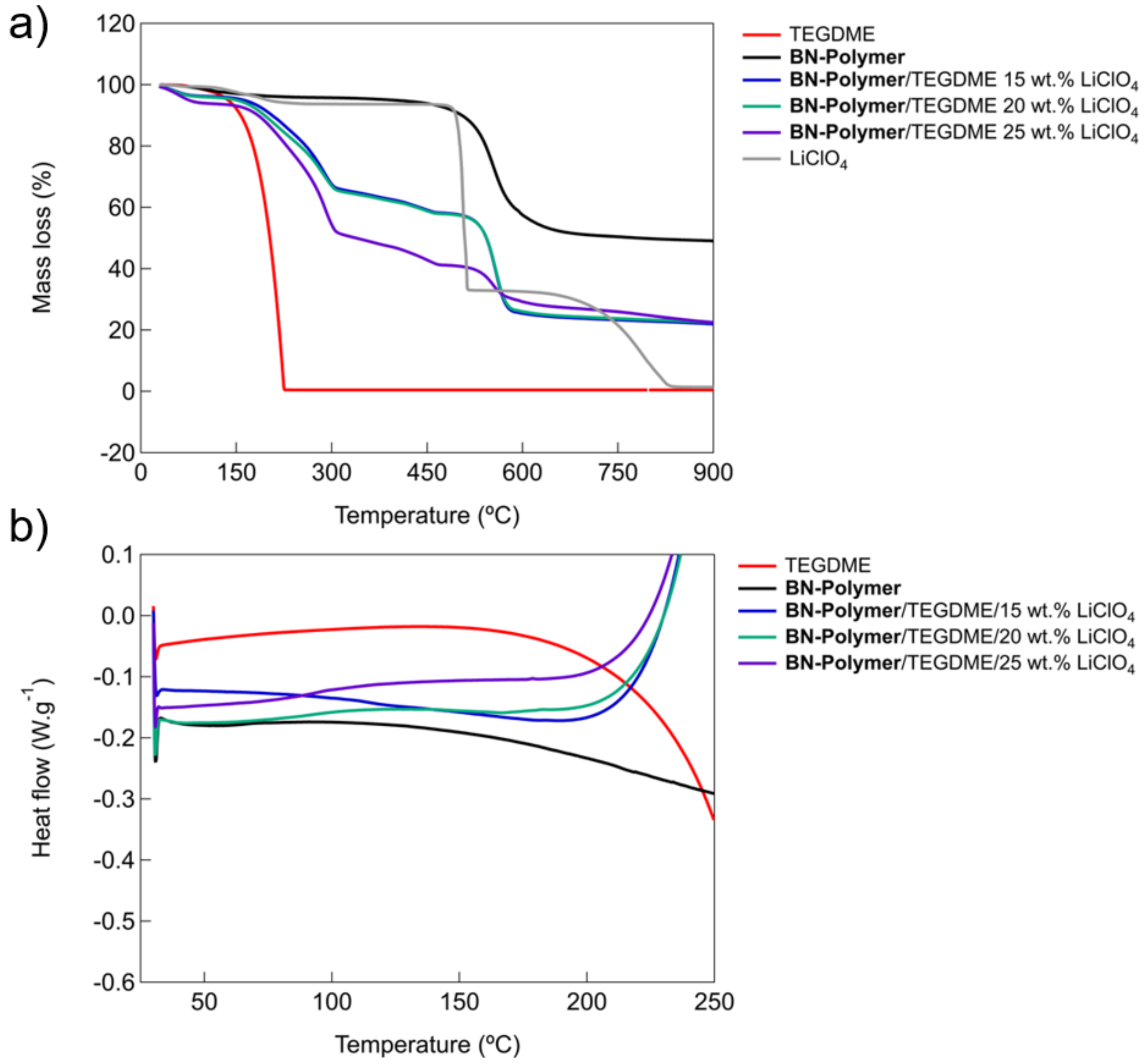

Figure S42. a) Thermogravimetric analysis (TGA). b) Differential scanning calorimetry (DSC) thermograms TEGDME, **BN-Polymer**, and **BN-Polymer**/TEGDME/$LiClO_4$ based electrolytes at different wt.% $LiClO_4$ contents (15, 20 and 25 wt.%).

6.4 Electrochemical study

In order to study the electrochemical stability of the SSE system, linear sweep voltammetry (LSV) and cyclic voltammetry (CV) were performed on the highest conducting **BN-Polymer**/TEGDME/$LiClO_4$ (42.5/42.5/15 wt.%) sample using stainless steel working electrode and Li metal as reference and counter electrode (Figure S43). The sample was assembled in an Argon filled glove box with H_2O and $O_2 < 0.1$ ppm in a symmetric stainless steel (SS)/**BN-Polymer**/TEGDME/$LiClO_4$/Li configuration.

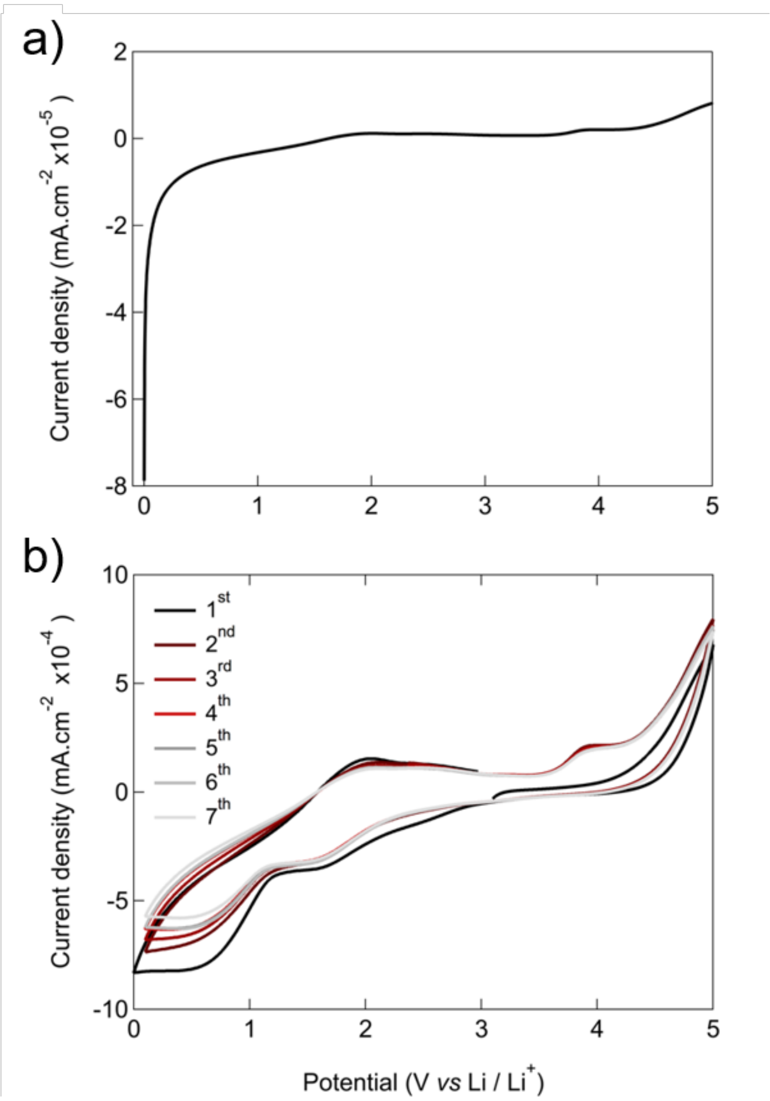

Figure S43. a) Linear sweep voltammetry of the **BN-Polymer**/TEGDME/15 wt.% $LiClO_4$ electrolyte at a scan rate of 1.0 $mV.s^{-1}$ from 0.0 to 5.0 V at r.t.. b) Cyclic voltammetry of the **BN-Polymer**/TEGDME/15 wt.% $LiClO_4$ electrolyte at a scan rate of 1.0 $mV.s^{-1}$ in the voltage range of 0.0 - 5.0 V *vs* Li/Li^+ at r.t..

The ion transport behaviour of the obtained SSEs was investigated by electrochemical impedance spectroscopy at r.t., using TEGDME/15 wt.% $LiClO_4$ liquid electrolyte as reference (Figure S44).

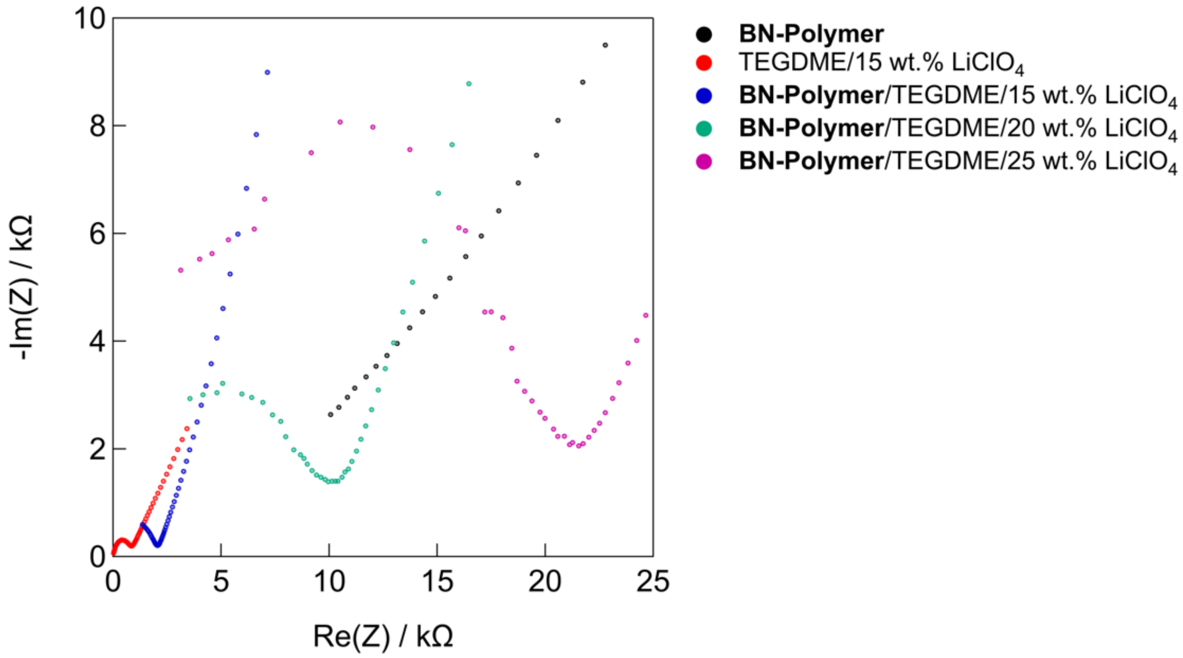

Figure S44. Nyquist plots of pure **BN-Polymer**, TEGDME/15 wt.% $LiClO_4$ and **BN-Polymer**/TEGDME/x $LiClO_4$ (x = 15, 20 or 25 wt.% of $LiClO_4$) at r.t. (zoom of the Figure 6a in the main manuscript).

7 Brunauer-Emmett-Teller measurements on BN-Polymer

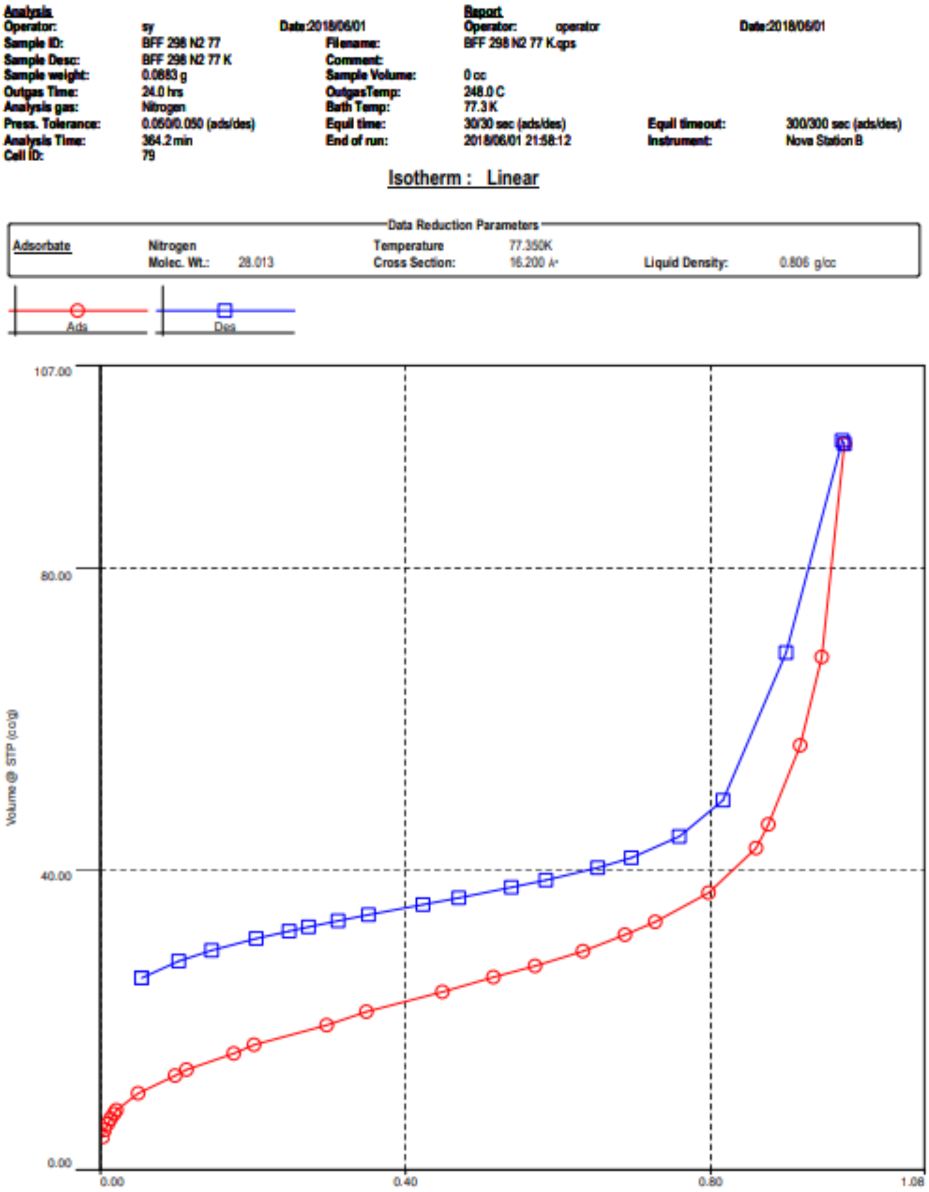

Figure S45. BET N_2 adsorption isotherms measured on **BN-Polymer** at 77 K.

8 References

[1] S. Meiboom, D. Gill, *Rev. Sci. Instrum.* **1958**, *29*, 688–691.

[2] A. Lausi, M. Polentarutti, S. Onesti, J. R. Plaisier, E. Busetto, G. Bais, L. Barba, A. Cassetta, G. Campi, D. Lamba, et al., *Eur. Phys. J. Plus* **2015**, *130*, 2–8.

[3] W. Kabsch, *Acta Crystallogr. Sect. D Biol. Crystallogr.* **2010**, *66*, 125–132.

[4] M. D. Winn, C. C. Ballard, K. D. Cowtan, E. J. Dodson, P. Emsley, P. R. Evans, R. M. Keegan, E. B. Krissinel, A. G. W. Leslie, A. McCoy, et al., *Acta Crystallogr. Sect. D Biol. Crystallogr.* **2011**, *67*, 235–242.

[5] P. R. Evans, G. N. Murshudov, *Acta Crystallogr. Sect. D Biol. Crystallogr.* **2013**, *69*, 1204–1214.

[6] G. M. Sheldrick, *Acta Crystallogr. Sect. A Found. Crystallogr.* **2015**, *71*, 3–8.

[7] G. M. Sheldrick, *Acta Crystallogr. Sect. C Struct. Chem.* **2015**, *71*, 3–8.

[8] P. Emsley, B. Lohkamp, W. G. Scott, K. Cowtan, *Acta Crystallogr. Sect. D Biol. Crystallogr.* **2010**, *66*, 486–501.

[9] A. L. Spek, *Acta Crystallogr. Sect. C Struct. Chem.* **2015**, *71*, 9–18.

[10] L. J. Farrugia, *J. Appl. Crystallogr.* **2012**, *45*, 849–854.

[11] L. L. C. Schröedinger, *PyMOL Molecular Graphic System http//www.pymol.org* **2015**.

[12] A. L. Spek, *Acta Crystallogr. Sect. D Biol. Crystallogr.* **2009**, *65*, 148–155.

[13] A. Le Bail, H. Duroy, J. . Fourquet, *Mat. Res. Bull.* **1988**, *23*, 447–452.

[14] G. Matz, X. Wu, S. O. Smith, *J. Magn. Reson. Ser. A* **1994**, *110*, 219–227.

[15] S. P. Brown, S. J. Heyes, S. Wimperis, *J. Magn. Reson. Ser. A* **1996**, *119*, 280–284.

[16] Y. Millot, P. P. Man, *Solid State Nucl. Magn. Reson.* **2002**, *21*, 21–43.

[17] M. M. Chui, R. J. Phillips, M. J. McCarthy, *J. Colloid Interface Sci.* **1995**, *174*, 336–344.

[18] L. Romero-Zerón, F. Manalo Hum, A. Kantzas, *SPE Reserv. Eval. Eng.* **2008**, *11*, 439–453.

[19] K. R. Brownstein, C. E. Tarr, *Phys. Rev. A* **1979**, *19*, 2446–2453.

[20] N. R. Draper, H. Smith, *Applied Linear Regression*, **1966**.

[21] R. Lapasin, S. Pricl, in *Rheol. Ind. Polysaccharides Theory Appl.*, Springer US, Boston, MA, **1995**, pp. 250–494.

[22] M. Abrami, I. D'Agostino, G. Milcovich, S. Fiorentino, R. Farra, F. Asaro, R. Lapasin, G. Grassi, M. Grassi, *Soft Matter* **2014**, *10*, 729–737.

[23] P. J. Flory, *Principles of Polymer Chemistry*, Cornell University Press, **1953**.

[24] J. Schurz, *Prog. Polym. Sci.* **1991**, *16*, 1–53.

[25] D. Dong, H. Zhang, B. Zhou, Y. Sun, H. Zhang, M. Cao, J. Li, H. Zhou, H. Qian, Z. Lin, et al., *Chem. Commun.* **2019**, *55*, 1458–1461.

[26] P. G. Bruce, J. Evans, C. A. Vincent, *Solid State Ionics* **1988**, *28–30*, 918–922.

[27] W. He, Z. Cui, X. Liu, Y. Cui, J. Chai, X. Zhou, Z. Liu, G. Cui, *Electrochim. Acta* **2017**, *225*, 151–159.

[28] S. Zugmann, M. Fleischmann, M. Amereller, R. M. Gschwind, H. D. Wiemhöfer, H. J. Gores, *Electrochim. Acta* **2011**, *56*, 3926–3933.

[29] S. Ibrahim, M. M. Yassin, R. Ahmad, M. R. Johan, *Ionics* **2011**, *17*, 399–405.

[30] S. Terada, T. Mandai, S. Suzuki, S. Tsuzuki, K. Watanabe, Y. Kamei, K. Ueno, K. Dokko, M. Watanabe,

J. Phys. Chem. C **2016**, *120*, 1353–1365.
